# LUBAC-mediated M1 Ub regulates necroptosis by segregating the cellular distribution of active MLKL

**DOI:** 10.1038/s41419-024-06447-6

**Published:** 2024-01-20

**Authors:** Nadine Weinelt, Kaja Nicole Wächtershäuser, Gulustan Celik, Birte Jeiler, Isabelle Gollin, Laura Zein, Sonja Smith, Geoffroy Andrieux, Tonmoy Das, Jens Roedig, Leonard Feist, Björn Rotter, Melanie Boerries, Francesco Pampaloni, Sjoerd J. L. van Wijk

**Affiliations:** 1 Institute for Experimental Paediatric Haematology and Oncology (EPHO), Goethe University Frankfurt, Komturstrasse 3a, 60528 Frankfurt am Main, Germany; 2 Physical Biology Group, Buchmann Institute for Molecular Life Sciences (BMLS), Biological Sciences (IZN), Goethe University Frankfurt, Max-von-Laue-Strasse 15, 60438 Frankfurt am Main, Germany; 3 Institute of Medical Bioinformatics and Systems Medicine, Medical Center-University of Freiburg, Faculty of Medicine, University of Freiburg, 79110 Freiburg, Germany; 4 GenXPro GmbH, Altenhoeferallee 3, 60438 Frankfurt am Main, Germany; 5 German Cancer Consortium (DKTK) partner site Freiburg and German Cancer Research Center (DKFZ), Heidelberg, Germany; 6 German Cancer Consortium (DKTK) partner site Frankfurt/Mainz and German Cancer Research Center (DKFZ), Heidelberg, Germany; 7 University Cancer Centre Frankfurt (UCT), University Hospital Frankfurt, Goethe-University Frankfurt, Frankfurt, Germany

**Keywords:** Cell biology, Biochemistry

## Abstract

Plasma membrane accumulation of phosphorylated mixed lineage kinase domain-like (MLKL) is a hallmark of necroptosis, leading to membrane rupture and inflammatory cell death. Pro-death functions of MLKL are tightly controlled by several checkpoints, including phosphorylation. Endo- and exocytosis limit MLKL membrane accumulation and counteract necroptosis, but the exact mechanisms remain poorly understood. Here, we identify linear ubiquitin chain assembly complex (LUBAC)-mediated M1 poly-ubiquitination (poly-Ub) as novel checkpoint for necroptosis regulation downstream of activated MLKL in cells of human origin. Loss of LUBAC activity inhibits tumor necrosis factor α (TNFα)-mediated necroptosis, not by affecting necroptotic signaling, but by preventing membrane accumulation of activated MLKL. Finally, we confirm LUBAC-dependent activation of necroptosis in primary human pancreatic organoids. Our findings identify LUBAC as novel regulator of necroptosis which promotes MLKL membrane accumulation in human cells and pioneer primary human organoids to model necroptosis in near-physiological settings.

## Introduction

Necroptosis, characterized by plasma membrane rupture, release of cytokines, chemokines and damage-associated molecular patterns (DAMPs), elicits potent immunogenic responses [1, 2] and underlies a wide variety of inflammatory and infectious diseases and tumor progression [3, 4]. Induction of necroptosis upon tumor necrosis factor (TNF) receptor 1 (TNFR1) activation relies on recruitment of the pro-survival TNFR1 complex I consisting of receptor-interacting protein kinase 1 (RIPK1), TNFR1-associated death domain protein (TRADD), TNFR-associated factor 2 (TRAF2), cellular inhibitor of apoptosis 1/2 (cIAP1/2) and the linear ubiquitin chain assembly complex (LUBAC) [3]. cIAP1/2- and LUBAC-mediated K63 and M1 poly-ubiquitination (poly-Ub) of complex I components, including RIPK1, initiate transforming growth factor β (TGFβ)-activated kinase 1 (TAK1) complex- and inhibitor of κB kinase (IKK) complex-dependent activation of pro-inflammatory mitogen-activated protein kinase (MAPK) and nuclear factor-κB (NF-κB) signaling [5–9]. Complex I destabilization through cIAP1/2 depletion and caspase-8 inhibition or depletion trigger the formation of a cytosolic amyloid-like necrosome complex [3], composed of RIPK1 and RIPK3, leading to their activation by cross- and auto-phosphorylation [10–13]. Eventually, phosphorylated RIPK3 recruits and phosphorylates MLKL at T357 and S358, resulting in the exposure of the MLKL four-helix bundle (4HB) domain, MLKL oligomerization and subsequent translocation to the plasma membrane [14–20]. Activated MLKL accumulates in hotspots to control plasma membrane disruption and ion channel fluxes, ultimately leading to necroptosis [17, 18, 21–23].

Necroptosis is tightly controlled by post-translational modification of RIPK1, RIPK3 and MLKL, including phosphorylation and ubiquitination [24–26]. Ubiquitination regulates protein function, stability and localization by covalent modification of substrates with poly-Ub chains, linked via K6, K11, K27, K29, K33, K48, K63, or M1-linked (linear) Ub [27–29]. M1 poly-Ub is mediated by the LUBAC E3 ligase, consisting of the catalytic subunit HOIP (RNF31), HOIL-1 (RBCK1) and Sharpin [30–33]. The catalytic activity of HOIP can be blocked by HOIPIN-8, a small-molecule inhibitor that covalently binds to the HOIP catalytic C885 and interferes with several residues in the linear ubiquitin chain determining domain (LDD) of HOIP, leading to specific loss of M1 poly-Ub, without affecting other K48 and K63 poly-Ub or total Ub levels [34, 35]. LUBAC controls pro-inflammatory and -survival signaling upon activation of immune receptors, such as TNFR1, via M1 poly-Ub of receptors, receptor-associated proteins or existing K63 poly-Ub chains, resulting in the recruitment of signaling complexes, such as the TAK1 complex and the IKK complex to activate downstream MAPK and NF-κB signaling [6, 8, 9, 36, 37]. Accordingly, LUBAC deficiency in mice, or in patients with genetic mutations in *HOIP*, *HOIL-1* or *Sharpin*, results in inflammatory disorders linked to deregulated NF-κB activity [30–32, 38–42]. Similarly, genetic mouse models lacking functional LUBAC are sensitized towards TNFR1- and RIPK1-dependent cell death [30, 31, 43–47] and HOIPINs mimic LUBAC loss-of-function mutations by inhibiting cytokine-induced canonical NF-κB signaling and by regulating TNFα-induced cell viability [34, 48, 49].

Apart from anti-necroptotic ubiquitination, removal of oligomerized MLKL from the plasma membrane via endosomal sorting complex required for transport (ESCRT)-mediated plasma membrane shedding and exocytic release of MLKL or via endocytosis counteracts necroptosis [22, 50]. The homologous Flotillin-1 and −2 (FLOT1/2) proteins reside in lipid rafts, highly ordered membrane structures in the plasma membrane, the endoplasmic reticulum (ER), the Golgi apparatus and endocytic compartments [51–54]. FLOT1/2 associate with cholesterol-rich membrane domains in the inner leaflet of cell membranes via the N-terminal stomatin, prohibitin, flotillin, HflK/C (SPFH) domain and oligomerize to heterotetramers through the C-terminal flotillin domain to create FLOT platforms [55, 56]. FLOTs mediate the regulation of cytoskeleton-membrane interactions and cell-cell contacts, clathrin-independent endocytosis and the establishment of protein complexes at the plasma membrane [57–65].

Despite recently acquired insights into the mechanisms that control the pro-necroptotic functions of activated MLKL, the question as to how necroptosis is regulated downstream of MLKL activation and oligomerization remains vastly unexplored. Here, we identify LUBAC-mediated M1 poly-Ub as a novel regulatory checkpoint in necroptotic cell death in cells of human origin. Suppression of LUBAC and M1 poly-Ub blocks TNFα-induced necroptosis without affecting necroptotic phosphorylation of RIPK1, RIPK3 or MLKL, necrosome formation and MLKL oligomerization. Loss of M1 poly-Ub suppresses MLKL membrane hotspot accumulation as well as MLKL-dependent release of pro-inflammatory signaling molecules. Finally, we confirm that loss of LUBAC activity prevents necroptosis in primary human pancreatic organoids (hPOs). Taken together, we identify a novel role for LUBAC and M1 poly-Ub in regulating membrane accumulation of activated MLKL and necroptosis. By modelling necroptotic cell death and LUBAC function in primary human organoids, we provide a novel experimental platform to study programmed cell death in intact human multicellular systems.

## Results

### LUBAC-mediated M1 poly-Ub is required for TNFα-induced necroptosis in human cells

**Fig. 1:**
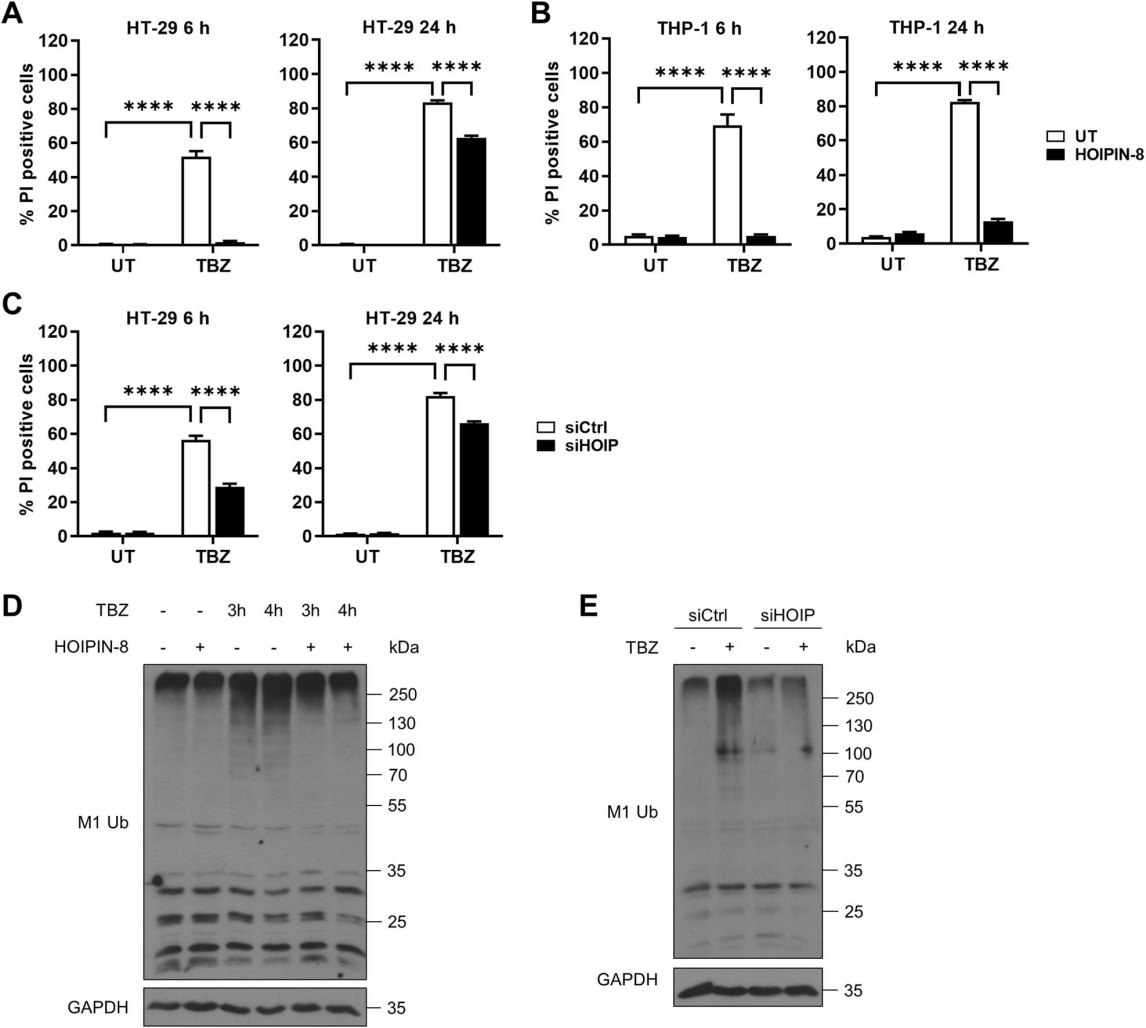
LUBAC-mediated M1 poly-Ub is required for TNFα-induced necroptosis in human cells. **A** Quantification of cell death in untreated (UT) and HOIPIN-8 (30 µM) pre-treated HT-29 cells after treatment with TBZ (10 ng/mL TNFα, 1 µM BV6, 20 µM zVAD.fmk) for the indicated time points. Mean and SEM of *n* = 3 independent experiments are shown. ****P < 0.0001. **B** Quantification of cell death in untreated (UT) and HOIPIN-8 (30 µM)-pre-treated THP-1 cells after treatment with TBZ (10 ng/mL TNFα, 1 µM BV6, 20 µM zVAD.fmk) for the indicated time points. Mean and SEM of *n* = 3 independent experiments are shown. ****P < 0.0001. **C** Quantification of cell death in control (siCtrl) and HOIP knock-down (siHOIP) HT-29 cells treated with TBZ (10 ng/mL TNFα, 1 µM BV6, 20 µM zVAD.fmk) for the indicated time points. Mean and SEM of *n* = 3 independent experiments are shown. ****P < 0.0001. **D** Western blot analysis of total M1 poly-Ub levels in control or HOIPIN-8 (30 µM)-pre-treated HT-29 cells upon treatment with TBZ (10 ng/mL TNFα, 1 µM BV6, 20 µM zVAD.fmk) for the indicated time points. GAPDH was used as loading control. Representative blots of at least two independent experiments are shown. **E**. Western blot analysis of total M1 poly-Ub levels in control (siCtrl) and HOIP knock-down (siHOIP) HT-29 treated with TBZ (10 ng/mL TNFα, 1 µM BV6, 20 µM zVAD.fmk) for 4 h. GAPDH was used as loading control. Representative blots of at least two independent experiments are shown. See also Figure S1. Statistical significance was determined using 2-way ANOVA followed by Tukey’s (**A**, **B**) or Sidak’s multiple comparisons test (**C**).

Necroptotic cell death is tightly regulated by several checkpoints, including compartmentalization of phosphorylated MLKL [22, 24, 50]. To investigate how LUBAC and M1 poly-Ub regulate necroptotic checkpoints, cell death was induced by TNFα (T), the Smac mimetic BV6 (B) and the pan-caspase inhibitor zVAD.fmk (Z). As anticipated, prominent necroptosis could be triggered by TBZ in human colon carcinoma HT-29 cells, as determined by quantification of propidium iodide (PI)-positive cells (Fig. 1A). Surprisingly, chemical inhibition of HOIP, the catalytic subunit of LUBAC, with the small-molecular weight inhibitor HOIPIN-8 [35], rescued TBZ-induced necroptosis in a time-dependent manner (Fig. 1A). Since LUBAC subunit levels are differentially regulated in myeloid cells as compared to other cell types [66, 67], the effect of HOIP inhibition was investigated in the human monocytic cell line THP-1. TBZ-induced necroptotic cell death in THP-1 cells could also be potently delayed by HOIPIN-8 (Fig. 1B), confirming a broader relevance of HOIPIN-8-mediated inhibition in necroptosis. Inhibition of LUBAC activity with the mycotoxin gliotoxin [68] also effectively blocked necroptotic cell death, without inducing additional cytotoxicity (Fig. S1A, B). Importantly, the HOIP inhibitor-based effects on necroptosis could be confirmed by siRNA-mediated knock-down of HOIP expression that rescued necroptosis to a similar extent (Fig. 1C and S1C). Total cellular levels of M1 poly-Ub increased upon induction of necroptosis, which could be efficiently reduced by HOIPIN-8 and siRNA-mediated knock-down of HOIP in HT-29 cells (Figs. 1D, E) and THP-1 cells (Figure S1D). Of note, M1 poly-Ub levels even further increased upon incubation with TBZ as compared to M1 poly-Ub levels induced by TNFα or by combined treatment with TNFα and BV6 (TB), indicating that M1 poly-Ub during TNFR1-mediated necroptosis is not solely the consequence of TNFα-induced NF-κB signaling (Figure S1E).

The role of M1 poly-Ub on necroptotic checkpoints was analyzed in further detail. Importantly, expression levels of the LUBAC subunits HOIP, HOIL-1 and Sharpin were not affected during necroptosis induction or HOIPIN-8-mediated inhibition (Figures S1F, G). Loss of either HOIP, HOIL-1 or Sharpin in mice interferes with TNFR1-mediated signaling, leading to impaired NF-κB activation and increased sensitivity towards TNFR1-triggered programmed cell death [30, 43–47, 69, 70]. Of note, HOIPIN-8 acts by interfering with the active site residue C885 as well as residues in the HOIP LDD [34], which are conserved in humans and mice (Figure S1H), and efficiently abrogated TNFα-induced NF-κB activation and stabilized phosphorylated IκBα in both HT-29 and mouse embryonic fibroblasts (MEFs) (Figures S1I, J), consistent with the described pro-inflammatory roles of LUBAC and M1 poly-Ub. However, to our surprise, HOIPIN-8-mediated LUBAC inhibition did not rescue TBZ-induced cell death in MEFs (Figure S1K), and even enhanced TBZ-induced cell death in L-929 cells and RAW 264.7 cells, without inducing cytotoxicity (Figures S1L, M), which could be effectively blocked by inhibition of RIPK3 with GSK’872 (Figures S1L, M). This is in accordance with previous findings demonstrating that RNAi-mediated silencing of HOIP or HOIL-1 sensitizes L-929 cells to necroptotic cell death [70]. Of note, the cellular levels of M1 poly-Ub only slighly increased upon necroptosis induction in MEFs (Figure S1N). Taken together, these observations, confirmed by a detailed analysis of cell death regulation in genetic mouse models of LUBAC deficiency [30, 43, 44, 69], suggest that LUBAC acts as a novel regulatory switch in the control of TNFα-induced necroptosis in cell lines of human origin.

### Loss of LUBAC-catalyzed M1 poly-Ub does not affect necroptotic signaling and necrosome formation

**Fig. 2:**
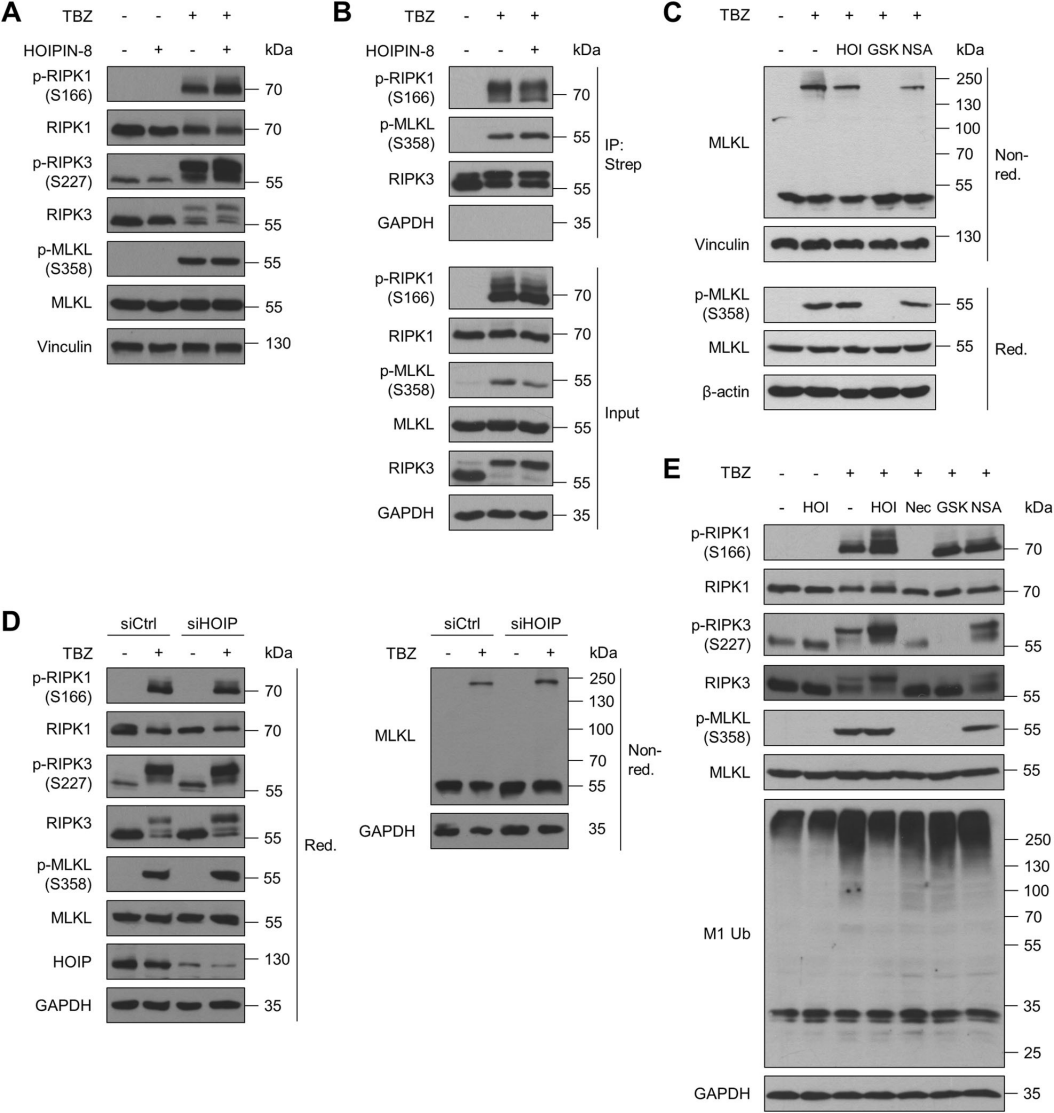
Loss of LUBAC-catalyzed M1 poly-Ub does not affect necroptotic signaling and necrosome formation. **A** Western blot analysis of phosphorylated and total expression levels of RIPK1, RIPK3 and MLKL in control *versus* HOIPIN-8 (30 µM)-pre-treated HT-29 cells upon treatment with TBZ (10 ng/mL TNFα, 1 µM BV6, 20 µM zVAD.fmk) for 4 h. Vinculin was used as loading control. Representative blots of at least two independent experiments are shown. **B** Immunoprecipitation of Strep-RIPK3 from control and HOIPIN-8 (30 µM)-pre-treated RIPK3 knock-out (KO) HT-29 cells re-expressing PAM-mutated Dox-inducible Strep-tagged RIPK3 wild-type (WT) was performed in order to analyze necrosome formation after treatment with TBZ (10 ng/mL TNFα, 1 µM BV6, 20 µM zVAD.fmk) for 3 h. Cells were incubated overnight with 1 μg/mL Dox prior to treatment to induce expression of Strep-RIPK3. GAPDH was used as loading control. Representative blots of at least two independent experiments are shown. **C** Western blot analysis of MLKL oligomerization upon treatment with TBZ (10 ng/mL TNFα, 1 µM BV6, 20 µM zVAD.fmk) for 4 h by non-reducing Western blotting of control, HOIPIN-8 (30 µM), GSK’872 (20 µM) or NSA (10 µM)-pre-treated HT-29 cells. Vinculin or β-actin were used as loading controls. Representative blots of at least two independent experiments are shown. **D** Western blot analysis of expression levels of phosphorylated and total RIPK1, RIPK3 and MLKL and MLKL oligomerization (by non-reducing Western blotting) upon 4 h TBZ (10 ng/mL TNFα, 1 µM BV6, 20 µM zVAD.fmk) treatment of control (siCtrl) and HOIP knock-down (siHOIP) HT-29 cells. GAPDH was used as loading control. Representative blots of at least two independent experiments are shown. **E**. Western blot analysis of expression levels of phosphorylated and total RIPK1, RIPK3, MLKL and M1 Ub upon 4 h TBZ (10 ng/mL TNFα, 1 µM BV6, 20 µM zVAD.fmk) treatment of HT-29 cells pre-treated with HOIPIN-8 (30 µM) or Nec-1s (30 µM), GSK‘872 (20 µM) and NSA (10 µM). GAPDH was used as loading control. Representative blots of at least two independent experiments are shown. See also Figure S2.

To mechanistically understand how LUBAC controls necroptosis, necroptosis signaling was investigated in detail. Surprisingly, HOIPIN-8 did not attenuate TBZ-induced phosphorylation of RIPK1 at S166, RIPK3 at S227 and MLKL at S358, major checkpoints in necroptotic cell death signaling (Fig. 2A and Figures S2A, B).

A central hallmark of necroptosis is the formation of the necrosome, composed of activated RIPK1 and RIPK3, that eventually phosphorylates MLKL to drive necroptotic cell death [10, 11, 14, 17]. Importantly, inhibition of LUBAC did not interfere with necrosome formation, as determined by co-immunoprecipitation of phosphorylated RIPK1 S166 and MLKL S358 with RIPK3 from untreated and HOIPIN-8-treated necroptotic cells (Fig. 2B).

Prior to membrane accumulation, activated MLKL oligomerizes into larger structures that are required for efficient necroptosis progression [17–20]. Upon induction of necroptosis, prominent MLKL oligomerization could be observed that, in contrast to GSK’872-mediated RIPK3 inhibition, could not be blocked by HOIPIN-8 (Fig. 2C). Furthermore, inhibiting MLKL with necrosulfonamide (NSA) did not affect MLKL phosphorylation and only slightly decreased MLKL oligomerization (Fig. 2C), as NSA only prevents membrane accumulation of activated MLKL [17]. Similarly, siRNA-mediated loss of HOIP also did not have any effect on necroptosis-induced RIPK1 S166, RIPK3 S227 and MLKL S358 phosphorylation and did not attenuate MLKL oligomerization (Fig. 2D). Inhibition of RIPK1 with necrostatin-1 (Nec-1s) or RIPK3 with GSK’872 influenced necroptosis-induced phosphorylation of RIPK1 S166, RIPK3 S227 and MLKL S358, respectively, while blocking MLKL with NSA did not affect phosphorylation levels (Fig. 2E). Of note, in contrast to HOIPIN-8, Nec-1s and GSK’872, as well as deletion of RIPK3 or MLKL, did not influence total M1 poly-Ub levels upon induction of necroptosis, whereas NSA slightly reduced M1 poly-Ub levels, suggesting that regulation of M1 poly-Ub occurs, at least partially, in parallel with necroptosis initiation and progression (Fig. 2E and S2B).

### LUBAC-mediated M1 poly-Ub controls MLKL-dependent production of inflammatory signaling molecules during necroptosis

**Fig. 3:**
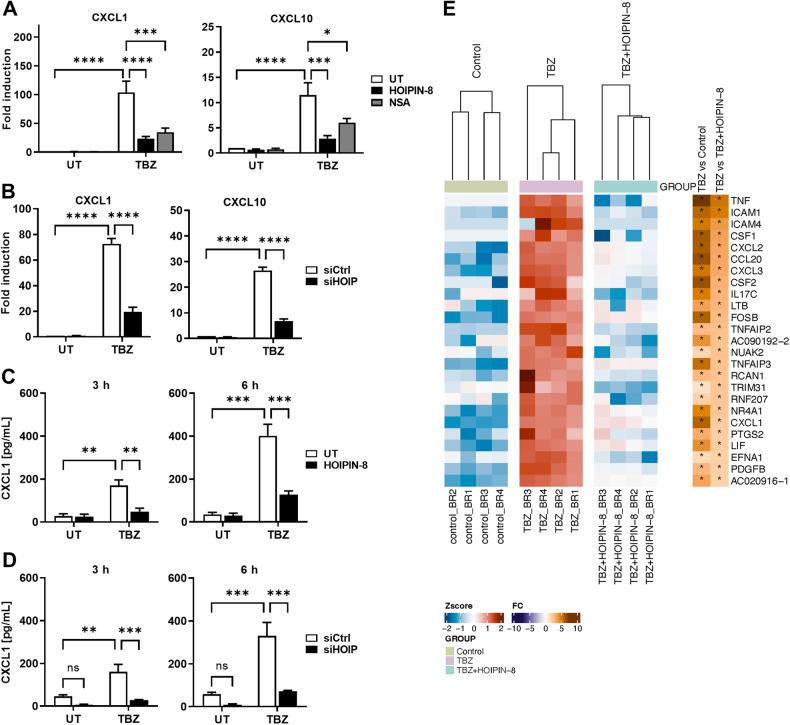
LUBAC-mediated M1 poly-Ub controls MLKL-dependent production of inflammatory signaling molecules during necroptosis. **A** mRNA expression levels of CXCL1 and CXCL10 of control (UT), HOIPIN-8 (30 µM) or NSA (10 µM)-pre-treated HT-29 cells upon treatment with TBZ (10 ng/mL TNFα, 1 µM BV6, 20 µM zVAD.fmk) for 3 h. Gene expression was normalized against 18 S and RPII mRNA expression and is presented as x-fold mRNA expression compared to the untreated (UT) control. Mean and SEM of *n* = 4 independent experiments are shown. *P < 0.05; ***P < 0.001; ****P < 0.0001. **B**. mRNA expression levels of CXCL1 and CXCL10 of control (siCtrl) or HOIP knock-down (siHOIP) HT-29 cells treated with TBZ (10 ng/mL TNFα, 1 µM BV6, 20 µM zVAD.fmk) for 4 h. Gene expression was normalized against 18 S and RPII mRNA expression and is presented as x-fold mRNA expression compared to the untreated (UT) control cells. Mean and SEM of *n* = 4 independent experiments are shown. ****P < 0.0001. **C**. ELISA-based quantification of CXCL1 secretion in supernatants of control (UT) or HOIPIN-8 (30 µM)-pre-treated HT-29 cells after treatment with TBZ (10 ng/mL TNFα, 1 µM BV6, 20 µM zVAD.fmk) for the indicated time points. Mean and SEM of *n* = 3 independent experiments are shown. **P < 0.01; ***P < 0.001. **D**. ELISA-based quantification of CXCL1 secretion in supernatants of control (siCtrl) or HOIP knock-down (siHOIP) HT-29 cells treated with TBZ (10 ng/mL TNFα, 1 µM BV6, 20 µM zVAD.fmk) for the indicated time points. Mean and SEM of *n* = 4 independent experiments are shown. **P < 0.01; ***P < 0.001. **E**. MACE-seq-determined heatmap showing the row-wise scaled intensity (left panel) of the top-25 upregulated genes in HT-29 cells treated with TBZ (10 ng/mL TNFα, 1 µM BV6, 20 µM zVAD.fmk) for 3 h in comparison to HOIPIN-8 (30 µM)-pre-treated and TBZ-treated HT-29 cells. Genes are sorted according to their log2 fold change values (right panel). *P < 0.05. See also Figure S3. Statistical significance was determined using 2-way ANOVA followed by Tukey’s multiple comparisons test (**A**–**D**).

During necroptosis, activated MLKL induces the transcriptional upregulation and secretion of a variety of inflammatory signaling molecules, including cytokines and chemokines, like CXCL1 and CXCL10 [1, 22]. Consistently with these findings, TBZ-triggered necroptosis strongly induced expression of CXCL1 and CXCL10 mRNA (Fig. 3A) in an MLKL-dependent manner, since inhibition of MLKL with NSA and CRISPR/Cas9-mediated knock-out (KO) of MLKL expression largely prevented necroptosis-induced upregulation of CXCL1 and CXCL10 mRNA (Fig. 3A and S3A). Interestingly, LUBAC inhibition or loss of HOIP expression in necroptotic cells reduced CXCL1 and CXCL10 mRNA expression (Figs. 3A, B), as well as CXCL1 secretion (Figs. 3C, D). In agreement with previous observations [1], necroptosis prominently triggered the transcriptional upregulation of a highly pro-inflammatory gene expression profile (Fig. 3E). Importantly, loss of LUBAC activity strongly attenuated the expression of necroptosis-upregulated cytokines and chemokines, such as TNFα, CSF1, CXCL2 and CCL20, as well as the adhesion molecule ICAM1 (Fig. 3E, Figures S3B, C). Of note, necroptosis-induced expression of TNFα and ICAM1 could be largely prevented by inhibiton of MLKL with NSA (Figures S3B, C), confirming that MLKL is required for necroptosis-induced upregulation of regulatory and inflammatory gene expression signatures. To exclude the possibility that HOIPIN-8-mediated decreases in cytokine production are due to the role of LUBAC in the regulation of TNFα-induced signaling, HT-29 cells were treated with TNFα and TBZ. In line with previous findings [1], necroptosis-mediated induction of CXCL1 mRNA expression was much stronger, as compared to cells treated with TNFα (Figure S3D). LUBAC inhibition efficiently blocked CXCL1 mRNA expression in TNFα- and TBZ-treated cells, while NSA only inhibited TBZ-induced CXCL1 levels (Figure S3D), further confirming the role of MLKL in necroptosis-induced cytokine expression. Together, these data suggest that M1 poly-Ub is required for the activated MLKL-dependent secretion of signaling molecules, cytokines and chemokines, such as CXCL1 and CXCL10, upon induction of necroptosis.

### LUBAC and M1 poly-Ub segregate the subcellular distribution of activated MLKL

**Fig. 4:**
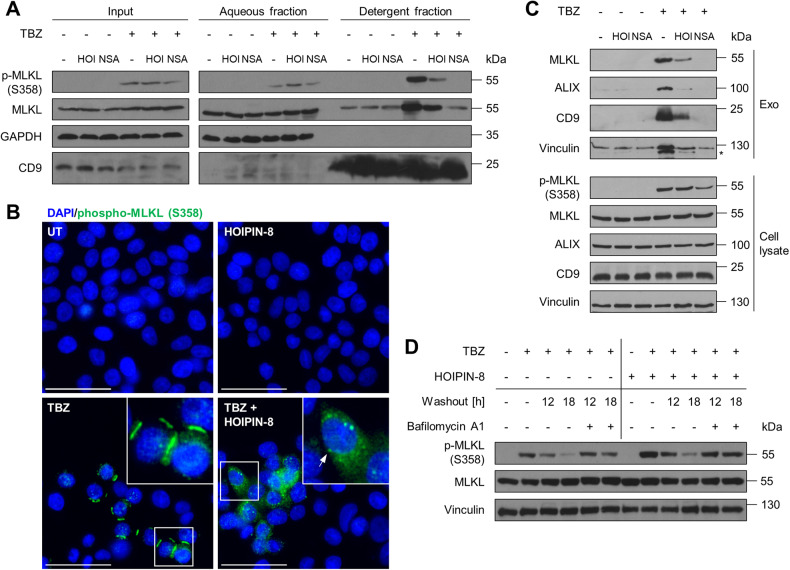
LUBAC and M1 poly-Ub segregate the subcellular distribution of activated MLKL. **A** Fractionation of phosphorylated and total MLKL in the micelle-poor (aqueous) and micelle-rich (detergent) fractions upon phase separation using Triton X-114 lysis buffer in untreated (UT) and HOIPIN-8 (30 µM) pre-treated HT-29 cells upon treatment with TBZ (10 ng/mL TNFα, 1 µM BV6, 20 µM zVAD.fmk) for 4 h. GAPDH was used as loading control for soluble proteins and CD9 as loading control for membrane proteins. Representative blots of at least two independent experiments are shown. **B** Representative fluorescence microscopy demonstrating the cellular distribution of phosphorylated MLKL (green) in untreated (UT) and HOIPIN-8 (30 µM) pre-treated HT-29 cells and after treatment with TBZ (10 ng/mL TNFα, 1 µM BV6, 20 µM zVAD.fmk) for 3 h. Nuclei were stained with DAPI (blue). Arrows indicate cytoplasmic clusters of phosphorylated MLKL. Representative images of at least two independent experiments are shown. Scale bars 50 µm. **C** Western blot analysis of TBZ-induced release of MLKL in exosomes, isolated from the supernatants of control, HOIPIN-8 (30 µM) or NSA (10 µM)-pre-treated HT-29 cells after treatment with TBZ (10 ng/mL TNFα, 1 µM BV6, 20 µM zVAD.fmk) for 3 h. Exosome isolation was confirmed with antibodies against ALIX, CD9 and Vinculin. Asterisk marks unspecific band. Expression of phosphorylated and total MLKL was determined by Western blotting of cell lysates from the same experiment. Vinculin was used as loading control. Representative blots of at least two independent experiments are shown. **D** Western blot analysis of lysosomal degradation of phosphorylated MLKL upon TBZ (10 ng/mL TNFα, 0.1 µM BV6, 20 µM zVAD.fmk) treatment of control, HOIPIN-8 (30 µM) or NSA (10 µM)-pre-treated HT-29 cells. HT-29 cells were treated with TBZ for 3 h and either harvested or the medium was exchanged with fresh medium with or without BafA1 (100 nM) after a washing step with PBS. Washed-out cells were harvested 12 h or 18 h after medium exchange. Vinculin was used as loading control. Representative blots of at least two independent experiments are shown. See also Figure S4.

During the effector phase of necroptosis, activated MLKL oligomers grow to form cytoplasmic clusters, which translocate to the cell membrane to accumulate into hotspots, preferentially at intercellular junctions [21]. Fractionation of detergent-enriched and aqueous fractions in lysates of necroptotic cells revealed extensive accumulation of phosphorylated MLKL in the detergent-enriched fraction, consistent with previous findings [17], which could be efficiently blocked by NSA and reduced by LUBAC inhibition (Fig. 4A). These data suggest that LUBAC-mediated M1 poly-Ub is a prerequisite for the translocation of activated MLKL from the cytosol to membrane compartments during necroptosis induction. Of note, necroptosis also triggered the accumulation of M1 poly-Ub chains in both aqueous and detergent-enriched fractions that could be counteracted by LUBAC inhibition (Figure S4A).

To further understand how LUBAC and M1 poly-Ub regulate the membrane localization of phosphorylated MLKL during necroptosis, MLKL distribution was assessed by immunofluorescence. Upon necroptosis induction, a fraction of S358-phosphorylated MLKL localized to membranes in large hotspots where neigbouring cells are in close contact (Fig. 4B, S4B, C), corresponding with previous findings [21]. As anticipated, MLKL inhibition with NSA blocked membrane accumulation of activated MLKL in necroptotic cells (Figure S4B). Intriguingly, HOIPIN-8 abrogated the formation of phosphorylated MLKL hotspots, leading to the appearance of diffuse cytoplasmic punctate clusters of S358-phosphorylated MLKL (Fig. 4B and S4C). No accumulation of M1 poly-Ub could be observed at intercellular membrane junctions during necroptosis (Figure S4D).

The cellular fate of phosphorylated MLKL determines the functional outcome of necroptotic signaling and is tightly controlled by endocytosis [50] and exocytosis of activated MLKL [22, 50, 71, 72]. TBZ-induced necroptosis triggered the formation of ALIX-, CD9- and vinculin-positive extracellular exosomes that contained high levels of MLKL, which could be strongly reduced by HOIPIN-8 and completely blocked by NSA (Fig. 4C). In line with previous data (Figure S2B), HOIPIN-8 induced a TBZ-dependent increase in MLKL S358 phosphorylation, and only partially affected lysosomal degradation of phosphorylated MLKL upon necroptosis induction, as determined after 18 h after washout of the necroptotic stimulus in the absence or presence of the lysosomal inhibitor bafilomycin A1 (BafA1) (Fig. 4D). Taken together, these data demonstrate that LUBAC-mediated M1 poly-Ub regulates necroptosis downstream of MLKL phosphorylation and oligomerization by controlling the availability and translocation of activated MLKL to membranes and MLKL hotspot formation at the plasma membrane.

### FLOT1/2 are co-purifying with M1 poly-Ub during necroptosis progression

**Fig. 5:**
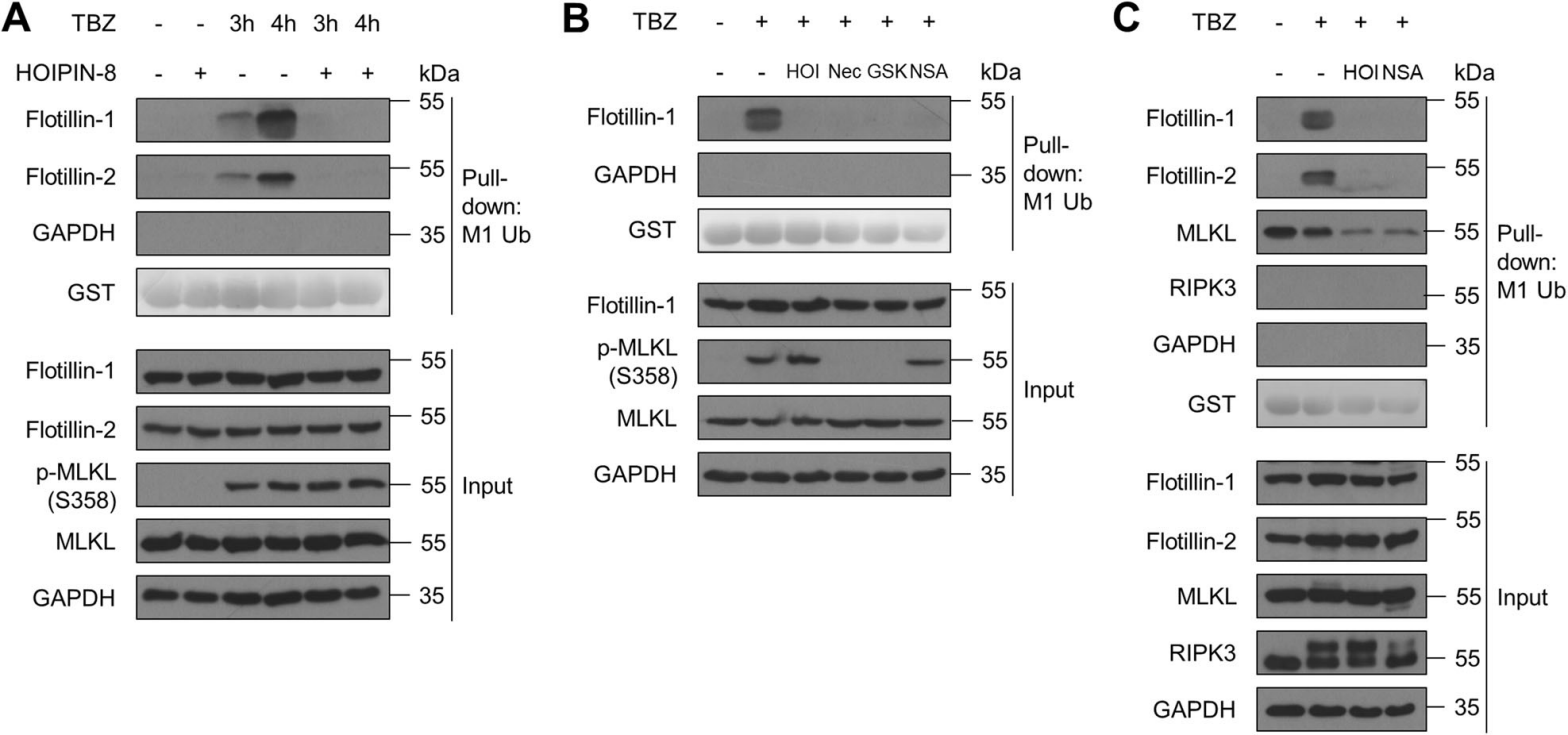
FLOT1/2 are co-purifying with M1 poly-Ub during necroptosis progression. **A** UBAN-mediated pulldown of M1 ubiquitinated proteins in control or HOIPIN-8 (30 µM)-pre-treated HT-29 cells treated with TBZ (10 ng/mL TNFα, 1 µM BV6, 20 µM zVAD.fmk) for the indicated time points. GST and GAPDH were used as loading controls. Representative blots of at least two independent experiments are shown. **B** UBAN-mediated pulldown of M1 ubiquitinated proteins in control, HOIPIN-8 (30 µM), Nec-1s (30 µM), GSK‘872 (20 µM) or NSA (10 µM)-pre-treated HT-29 cells treated with TBZ (10 ng/mL TNFα, 1 µM BV6, 20 µM zVAD.fmk) for 4 h. GST and GAPDH were used as loading controls. Representative blots of at least two independent experiments are shown. **C** GST-UBAN-mediated pulldown of M1 ubiquitinated proteins in control, HOIPIN-8 (30 µM) or NSA (10 µM)-pre-treated HT-29 cells treated with TBZ (10 ng/mL TNFα, 1 µM BV6, 20 µM zVAD.fmk) for 4 h. GST and GAPDH were used as loading controls. Representative blots of at least two independent experiments are shown. See also Figure S5.

FLOT1/2 colocalize with MLKL during necroptosis [50, 72]. Interestingly, high levels of FLOT1/2 could be enriched by capture of M1 poly-Ub via ubiquitin binding in ABIN and NEMO (UBAN)-based pulldowns from necroptotic HT-29 and THP-1 cells (Fig. 5A and S5A), but not by GST only (Figure S5B). RIPK1, a known M1 poly-Ub target, could be enriched by UBAN pulldown during necroptosis and M1 poly-Ub-modification of RIPK1 was reduced by HOIPIN-8 (Figure S5B). HOIPIN-8-mediated LUBAC inhibition completely prevented M1 poly-Ub-associated enrichment of FLOT1/2, without affecting total FLOT1/2 cellular expression levels (Fig. 5A and S5A). Enrichment of FLOT1 could also be prevented by inhibition of RIPK1 with Nec-1s, RIPK3 with GSK’872 and MLKL with NSA, indicating that activation and membrane translocation of MLKL is a prerequisite for the association of FLOT1 with M1 poly-Ub (Fig. 5B). Of note, MLKL could be detected in UBAN-based pulldowns under basal conditions, while RIPK3 was completely absent (Fig. 5C). To address the question as to whether FLOT2 or MLKL are directly modified with M1 poly-Ub, denaturing UBAN pulldowns were performed, but these did not show enrichment of M1 poly-Ub, suggesting loss of ubiquitin-binding during denaturing conditions (Figure S5B). Denaturing immunoprecipitations of FLOT2 and MLKL did not show covalent modification with M1 poly-Ub, indicating that direct M1 poly-Ub-based modification of MLKL and FLOT1/2 during necroptosis is unlikely (Figure S5C, D).

### LUBAC-mediated M1 poly-Ub regulates necroptotic cell death in primary hPOs

**Fig. 6:**
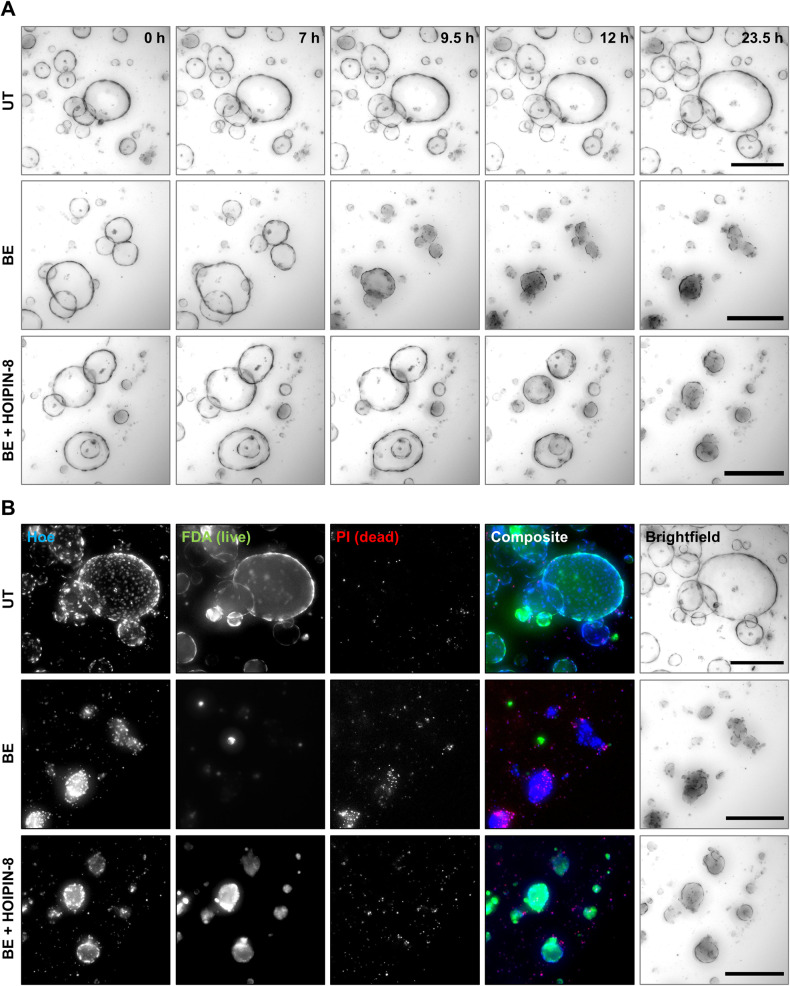
LUBAC-mediated M1 poly-Ub regulates necroptotic cell death in primary hPOs. **A** Representative images of time-lapse videos of untreated (UT), BV6 (1 µM) and Emricasan (10 µM) (BE)-treated or HOIPIN-8 (30 µM)-pre-treated and BE (BE + HOIPIN-8)-treated primary hPOs for a total period of 23.5 h. Scalebars: 250 µm. **B** *Idem* as **A**., but treated primary hPOs were stained with Hoechst33342 (Hoe; blue), FDA (live; green) and PI (dead; red) after 24 h treatment with BE or BE + HOIPIN-8 prior to imaging. Scalebars: 250 µm. Representative images of at least two independent experiments are shown (**A,** **B**). See also Figures S6 and S7 and Movies 1 and 2.

Our data reveal that LUBAC and M1 poly-Ub act as novel checkpoints that control the membrane hotspot accumulation of activated MLKL and thereby regulate necroptotic cell death in human cell lines. To further confirm this concept and to exclude artefacts in necroptotic signaling related to the use of human cancer cell lines, LUBAC- and M1 poly-Ub-mediated regulation of necroptosis was studied in adult stem cell-derived primary hPOs [73–76]. hPOs were maintained in matrix for 3 to 5 days to allow the formation of organoid spheres composed of a cellular monolayer surrounding a liquid-filled lumen, that expanded over time (Fig. 6A and S6A, B), in line with previous findings [77]. For necroptosis induction, hPOs were incubated with BV6 (B) and the clinically applicable pan-caspase inhibitor IDN-6556 (Emricasan; E), followed by time-lapse imaging and subsequent fluorescein diacetate (FDA)- and PI-based live/dead imaging. BE induced a prominent loss of luminal hPO morphology, characterized by organoid collapse and compaction, with individual cells migrating away from the collapsed organoids (Fig. 6A, Movie 1). Live/dead staining of BE-incubated organoids revealed high levels of PI-positive cells and low levels of FDA-positive cells, confirming prominent hPO cell death (Fig. 6B and S6A). Similar organoid collapse and loss of luminal morphology was observed in hPOs incubated with the Smac mimetic Birinapant (Bi) and Emricasan (Figure S6B, Movie 2) and upon incubation with BV6 and zVAD.fmk (BZ) (Figure S7). Importantly, HOIPIN-8-mediated inhibition of LUBAC delayed BE-induced organoid collapse, prevented the loss of luminal hPO morphology and extended hPO viability up to 24 h (Figs. 6A, B, Movie 1). HOIPIN-8 also blocked organoid collapse and cell death induced by BiE and BZ (Figures S6B, C, Figure S7, Movie 2). Of note, organoid collapse and compaction, loss of hPO morphology and cell death triggered by BiE could be blocked by NSA, indicating the occurrence of MLKL-dependent necroptotic cell death (Figure S6, Movie 2).

In conclusion, we identify LUBAC-mediated M1 poly-Ub as a novel checkpoint that regulates necroptosis by suppressing MLKL plasma membrane localization in human cell lines and primary pancreatic organoids.

## Discussion

M1-linked poly-Ub, catalyzed by the LUBAC E3 ligase, balances pro- and anti-survival measures. In contrast to genetic models of LUBAC deficiency in mice, in which LUBAC-mediated M1 poly-Ub represses cell death [30, 31, 43–47, 69], we found that pharmacological inhibition of LUBAC and siRNA-mediated loss of LUBAC prevents TNFα-induced necroptosis in human cell lines. Necroptosis triggered M1 poly-Ub that was not dependent on the activity or the physical presence of RIPK1, RIPK3 and MLKL, suggesting that increases in M1 poly-Ub during necroptosis are not regulated by the core necroptotic machinery. M1 poly-Ub levels prominently increased upon TBZ-induced necroptosis and to a lesser extent by TNFα alone or TB, suggesting that the necroptotic increase in M1 poly-Ub is not only dependent on TNFα. Loss of LUBAC activity reverts the cellular levels of necroptosis-induced M1 poly-Ub in human cell lines, although to a lesser degree in cell lines of murine origin. LUBAC inhibition abrogated TNFα-induced NF-κB signaling to the same extent in cell lines from both species, suggesting the specificity of LUBAC regulation during necroptosis. Importantly, species-specific coevolution and divergence of RIPK3 and MLKL resulted in important mechanistic and functional differences between human and murine necroptotic signaling pathways [78–81]. Human and murine MLKL cannot complement necroptotic signaling when expressed in cells of the opposing species [78–80]. Mouse RIPK3 and MLKL interact transiently, as compared to the activation of human MLKL that requires recruitment and binding to RIPK3 [78], which indicates additional roles for T357/S358 phosphorylation in human MLKL [78].

Loss of LUBAC activity did not alter necroptotic phosphorylation of RIPK1, RIPK3 and MLKL, necrosome formation or the oligomerization of MLKL, suggesting that LUBAC-dependent signaling events might become activated in parallel that involve M1 poly-Ub to eventually affect necroptosis. TNFα-induced necroptosis has been linked to the cell-autonomous upregulation and increased secretion of signaling molecules, including cytokines and chemokines [1, 22]. This involves two phases, the first of which depends on TNFα-induced NF-κB-mediated upregulation of inflammatory signaling molecules, whereas the second phase relies on necroptotic activation of RIPK1 and RIPK3, as well as MLKL [1, 22]. Our experiments reveal that LUBAC-dependent regulation affects both, necroptotic NF-κB- and MLKL-dependent production of inflammatory cytokines. In addition, the expression of CXCL1, a prototypic chemokine whose expression is linked to necroptosis and MLKL activation [1, 22], strongly increased upon TBZ-induced necroptosis, compared to TNFα alone, demonstrating that the increases in chemo- and cytokines are not solely a consequence of TNFα and NF-κB, but also rely on the activated necroptosis machinery. Taken together, these findings confirm parallel and independent LUBAC-dependent signaling events that converge downstream of activated MLKL to allow efficient membrane localization and necroptosis.

LUBAC inhibition prevents hotspot accumulation of phosphorylated MLKL as well as exocytosis in extracellular vesicles, suggesting that LUBAC-mediated M1 poly-Ub supports MLKL membrane tethering or clustering of activated MLKL. Intriguingly, the M1 poly-Ub-dependent effects on MLKL mimic MLKL-specific monobodies that target the 4HB of human MLKL to inhibit necroptosis, but without affecting necrosome formation, MLKL activation and MLKL oligomerization [82]. Our findings suggest that M1 poly-Ub might affect MLKL clustering and translocation via the MLKL 4HB to inhibit necroptosis. Interestingly, pharmacological interference of MLKL trafficking with inhibitors of Golgi, microtubule and actin networks (e.g. with Brefeldin A, Nocodazole and Cytochalasin A, respectively) also prevented membrane hotspot formation of activated MLKL [21]. Loss of LUBAC triggered a similar phenotype, suggesting that LUBAC-mediated M1 poly-Ub might be involved in the regulation of MLKL trafficking. Based on these findings, we propose that M1 poly-Ub is important for MLKL-mediated intracellular transport, membrane accumulation and exosome formation, and is involved in lysosomal degradation of activated MLKL.

Both endo- and exocytic events balance MLKL membrane accumulation and involve the lipid raft-resided proteins FLOT1/2 [50]. Our findings point towards a M1 poly-Ub-dependent partitioning of MLKL in exosomes during necroptosis and suggest that membrane accumulation of activated MLKL is required for incorporation of MLKL in necroptotic exosomes. Mislocalized, phosphorylated MLKL accumulates in the cytosol and a fraction of activated MLKL is targeted for lysosomal degradation in a M1 poly-Ub-dependent manner. Intruigingly, FLOT1 is implicated in the transfer of ubiquitinated cargo between different ESCRT complexes by modulating the Ub-binding of hepatocyte growth factor-regulated tyrosine kinase substrate (Hrs) upon epidermal growth factor (EGF) stimulation and release of Ub-mediated autoinhibition of the Hrs ubiquitin-interacting motif (UIM) domain [83]. In line with this notion, we detected a strong necroptosis-induced and LUBAC-dependent enrichment of FLOT1/2 in M1 poly-Ub-based pulldowns that was dependent on the activation of RIPK1, RIPK3 and MLKL. Although the necroptosis-induced increase in cellular M1 poly-Ub levels does not depend on MLKL, inhibition of MLKL membrane translocation reduced co-enrichment of FLOT1, suggesting a link for FLOT1/2 in M1 poly-Ub and MLKL membrane localization and further demonstrating independent LUBAC signaling events during necroptosis. Of note, our findings could not confirm direct modification of FLOT2 or MLKL with M1 poly-Ub and further studies are warranted to identify the mechanisms of M1 poly-Ub-mediated necroptosis regulation.

MLKL undergoes mono-Ub upon oligomerization at membranes that might direct MLKL for proteasomal and lysosomal degradation [84]. Mutation of the identified mono-Ub sites in the MLKL 4HB did not prevent necroptosis, whereas fusion of the non-specific DUB USP21 to MLKL did sensitize towards necroptosis [84]. In addition, K63 poly-Ub of MLKL at K219 increased sensitivity towards necroptosis [85] and the homologue residue in human MLKL, K230, is also ubiquitinated [86]. Importantly, the Ub chain-specificity of the Ub-binding UBAN matrix excludes binding to K63-linked Ub chains [87]. Although MLKL could be detected in UBAN-based pulldowns of M1 ubiquitinated proteins in basal and necroptotic cells and HOIPIN-8 and NSA reduced the amount of UBAN-enriched MLKL, it remains unclear whether MLKL itself is directly modified with M1 poly-Ub or associates with M1 poly-Ub-modified proteins.

Taken together, we identified LUBAC-mediated M1 poly Ub as novel regulatory checkpoint that controls necroptosis in multicellular, primary human organoids ex vivo. The unique and first-time use of human primary organoids to systematically model necroptosis and the influence of LUBAC-mediated M1 poly-Ub in primary human organoids provides novel and relevant experimental models to study necroptosis in intact multicellular, near-physiological settings. Apart from necroptosis-proficient cell lines, experimental models to study necroptosis in human cells are extremely limited and so far no multicellular human models have been described. Since necroptosis underlies a wide variety of disease states, modeling necroptotic cell death in primary organoids will significantly contribute to gain new fundamental insights and to develop novel therapeutic options for emerging human diseases.

## Methods

### Cell lines, reagents and chemicals

The human HT-29 adenocarcinoma cell line was obtained from DMSZ (Braunschweig, Germany) and the human acute monocytic leukemia cell line THP-1 was kindly provided by Thomas Oellerich (Frankfurt am Main, Germany). MEFs were kindly provided by Joanne Hildebrand (Melbourne, Australia). The L-929 mouse fibroblast cell line was obtained from ATCC (Manassas, VA, USA). The RAW 264.7 mouse macrophage cell line was provided by Stefan Müller (Goethe University Frankfurt). HT-29 cell lines were cultured in McCoy’s 5 A Medium +GlutaMAX^TM^-I (Thermo Fisher Scientific, Waltham, MA, USA) supplemented with 1% penicillin/streptomycin (Thermo Fisher Scientific) and 10% fetal bovine serum (FBS) (Thermo Fisher Scientific). THP-1 cells were maintained in RPMI1640 Medium +GlutaMAX^TM^-I (Thermo Fisher Scientific) supplemented with 1% sodium pyruvate (Thermo Fisher Scientific), 1% penicillin/streptomycin and 10% FBS. HEK293T cells and MEFs were cultured in DMEM +GlutaMAX^TM^-I (Thermo Fisher Scientific) supplemented with 1% sodium pyruvate, 1% penicillin/streptomycin and 10% FBS. L-929 cells were cultured in DMEM +GlutaMAX^TM^-I (Thermo Fisher Scientific) supplemented with 1% sodium pyruvate, 1% penicillin/streptomycin, 1% MEM Non-Essential Amino Acids (NEAA) Solution (Thermo Fisher Scientific) and 10% FBS. RAW 264.7 cells were maintained in DMEM +GlutaMAX^TM^-I (Thermo Fisher Scientific) supplemented with 10% FBS. HT-29 RIPK3 KO and HT-29 RIPK3 KO cells re-expressing PAM-mutated doxycycline (Dox)-inducible Strep-tagged wild-type (WT) RIPK3 have been described previously [88]. All cell lines were cultured at 37 °C with 5% carbon dioxide in a humidified atmosphere and regularly monitored for mycoplasma infection. Cell line authentication of HT-29, THP-1, HEK293T and L-929 cells was performed by the Leibniz-Institute DSMZ GmbH (Braunschweig, Germany). HT-29, THP-1 and HEK293T were authenticated by DNA profiling using 8 different highly polymorphic short tandem repeat (STR) loci. L-929 were subjected to DNA barcoding of Cytochrome oxidase subunit 1 (COI) to confirm *Mus musculus* origin.

BV6 was kindly provided by Genentech Inc. (San Francisco, CA, USA), recombinant human TNFα was purchased from Biochrom Ltd. (Waterbeach, UK), Birinapant from Selleckchem (Houston, TX, USA), the pan-caspase inhibitors zVAD.fmk and Emricasan from Bachem AG (Bubendorf, Switzerland) and Selleckchem, Nec-1s, GSK’872 and NSA from Merck KGaA (Darmstadt, Germany), HOIPIN-8 from Axon Medchem LLC (Reston, VA, USA), Gliotoxin from Tocris Biosciences (Bristol, UK), Doxycycline hydrochloride from Merck KGgA (Darmstadt, Germany) and BafA1 from Selleckchem. All other chemicals were purchased from Carl Roth (Karlsruhe, Germany) or Merck KGaA, unless stated otherwise.

### Maintenance of primary hPOs

Primary hPOs are derived from the pancreatic duct of a male patient (age: 60, BMI: 26.3) and were provided by Lorenza Lazzari (Policlinico di Milano, Milan, Italy). The use of these hPOs is approved by the Institutional Review Board and the Pancreatic Islet Processing Unit, a National Transplant Center accredited facility (IT000679, https://webgate.ec.europa.eu/eucoding/reports/te/index.xhtml). hPOs were kept in an incubator at 37 °C in a humidified atmosphere with 5% CO_2_, embedded in Cultrex Reduced Growth Factor BME, Type 2 (BME-2) droplets (R&D Systems, Minneapolis, MN, USA) and covered under medium. Medium was fully changed every 2 to 3 days and hPOs were passaged every 7 to 14 days depending on the proliferation rate and organoid size.

The organoid culture protocol was adapted from described procedures [74]. Briefly, hPOs were maintained in basal medium, composed of Advanced DMEM/F-12 (Thermo Fisher Scientific) with 1x GlutaMAX™ Supplement (Thermo Fisher Scientific), 100 U/mL penicillin/streptomycin (Thermo Fisher Scientific) and 10 mM HEPES (Thermo Fisher Scientific), supplemented with 1x B-27™ (minus vitamin A, Thermo Fisher Scientific), 1x N-2 (Thermo Fisher Scientific), 2,5 mM *N*-acetylcysteine (MilliporeSigma, St. Louis, US), 1 µg/mL recombinant human R-Spondin1 (PeproTech, Rocky Hill, US), 10 mM nicotinamide (MilliporeSigma), 50 ng/mL recombinant human EGF (PeproTech), 100 ng/mL recombinant human FGF-10 (PeproTech), 25 ng/mL Noggin (PeproTech), 10 nM [Leu^15^]-Gastrin I (MilliporeSigma), 5 µM A83-01 (Tocris Bioscience, Minneapolis, US), 10 µM Forskolin (Tocris Bioscience) and 3 µM Prostaglandin E_2_ (Tocris Bioscience) (hPO medium).

For passaging of the primary hPOs, BME droplets were disrupted by pipetting and organoids were retrieved by centrifugation in ice-cold hPO medium. Organoids were sheared into fragments prior to resuspension in ice-cold liquified BME-2 and seeded as droplets in pre-warmed non-treated CELLSTAR® 48-well suspension culture plates (Greiner Bio-One GmbH, Kremsmünster, Austria). Organoid-containing BME-2 droplets were polymerized for 15 min at 37 °C after which the droplets were overlaid with hPO medium.

### Generation of HT-29 MLKL CRISPR/Cas9 KO cells

Generation of HT-29 MLKL CRISPR/Cas9 KO cells has been performed as described previously [88]. In brief, guide RNAs for human MLKL (#1 ATGACAATGGAGAATTGAGG; #2 CCTGTTTCACCCATAAGCCA; #3 TTCCCTTAGCAGAATCCACG) [89] were cloned into the LentiCRISPR v2 plasmid (Addgene plasmid #52961) [90] using standard cloning techniques and verified by Sanger DNA seqencing. For the production of lentiviral particals, HEK293T cells were co-transfected with MLKL guide RNAs, pMD2.G (Addgene plasmid #12259) and psPAX2 (Addgene plasmid #12260) using FuGENE HD Transfection Reagent (Promega) (DNA/FuGENE ratio 1:3) according to the manufacturer’s protocol in DMEM without penicillin/streptomycin. The medium was changed to full medium 24 h post-transfection and the viral supernatant was collected 48 h and 72 h post-transfection, pooled and sterile filtered through a 0.45 µm filter (MerckMillipore, Darmstadt, Germany). For target cell transduction, 0.5 mL of viral supernatant was added to the cells and the medium was supplemented with 8 µg/mL polybrene (Merck Sigma). 48 h post-transduction, the medium was exchanged with selection medium containing 12.5 µg/mL puromycin. Single-cell selection was performed in 96-well plates and MLKL KO colonies were expanded and verified by Western blotting.

### Induction and quantification of necroptotic cell death

The indicated cells were seeded in CELLSTAR® 96-well tissue culture plates (Greiner Bio-One GmbH) at appropriate densities the day before treatment. For necroptosis induction, cells were pretreated with 1 µM BV6 and 20 µM zVAD.fmk for 30 min (if not stated otherwise), followed by stimulation with 10 ng/mL recombinant human TNFα for the indicated time (TBZ). The inhibitors HOIPIN-8 (30 µM), Gliotoxin (1 µM), Nec-1s (30 µM), GSK’872 (20 µM) and NSA (10 µM) were added 1 h prior to TBZ treatment. Cell death was determined by PI and Hoechst33342 (Hoe) staining and fluorescence-based quantification of dead (PI-positive) cells compared to the total cell number (Hoe-positive cells) using an ImageXpress® Micro XLS Widefield High-Content Analysis System and MetaXpress® Software (Molecular Devices Sunnyvale, CA, USA).

### Induction and imaging of necroptotic cell death in primary hPOs

hPOs were seeded in 5 µL BME-2 droplets in sterile CELLSTAR® 96-well suspension culture plates (Greiner Bio-One GmbH) and overlaid with 100 µL hPO medium. hPOs were cultured for 3-5 days prior to experiments to assure organoid formation and growth. For pre-treatment with HOIPIN-8, hPO medium was replaced by hPO medium with the indicated concentrations of HOIPIN-8 and hPOs were placed back in the incubator. After 30 min, hPO medium was again replaced with medium supplemented with the indicated treatments and the plates were transferred to the Zeiss AxioObserver Z1 microscope. Incubation was done at 37 °C and 5% CO_2_ during which 24 h time lapse imaging in 30 min intervals was performed. Each well was imaged as tiles of two x two with eleven Z-slices of 60 µm thickness.

For live/dead assays, primary hPOs were stained using 10 µg/mL PI (Millipore Sigma), 0.5 µg/mL FDA (Millipore Sigma) and 200 µg/mL Hoe (ThermoFisher Scientific) for 30 min. Dyes were removed and the wells were washed with pre-warmed PBS prior to imaging in PBS, using the same microscope settings as described above.

Images were exported using the ZEN 2.6 lite software (Carl Zeiss Microscopy GmbH, Oberkochen, Germany) and subsequently processed using the FiJi/ImageJ software.

### Quantification of lumen volume in primary hPOs

The acquired brightfield data sets were integrated in a segmentation pipeline that has been published previously [91]. For segmentation, the images are processed using the MorphoLibJ plug-in in FiJi. Images from the Zeiss CellObserver Widefield microscope were exported as tiles and stitched and minimum projections were created prior to segmentation. The pixel color range was set to 8-bit and the background was subtracted using a rolling ball radius of 500.0 pixels with a light background. Images were inversed and the contrast was enhanced by 0.35% of saturated pixel. A median filter with a radius of two pixels was applied subsequently and the morphological segmentation was performed for border images with a tolerance value between 6 and 8 by MorphoLibJ. Using the Label Editor by MorphoLibJ, the labels are corrected and merged, if required. The labels were closed and a size opening of 500 pixels was performed, followed by analysis by Region Morphometry. Each label corresponded to one organoid detected in the image. Organoids which were not segmented in more than a quarter of all time points were excluded from the analysis. The analysed data was imported into Excel and the resulted organoid area measurements were further processed. The organoid areas ( = lumen volume) were normalized to the starting value at time point 0 and the average of all organoids in one condition, including the three replicates, were calculated and presented as standard errors of the mean.

### RNAi-mediated silencing

Transient genetic silencing of HOIP was performed by reverse transfection of HT-29 cells with the following siRNAs: human siHOIP (RNF31) (Silencer® Select siRNA #1 s30108, #2 s30109, #3 s30110, 40 nM) (Thermo Fisher Scientific, Waltham, MA, USA), non-targeting control siRNA (#4390843, 40 nM) (Thermo Fisher Scientific). In brief, the indicated cell lines were prepared in penicillin/streptomycin-free medium and combined with the siRNA-lipid complexes, prepared in OptiMEM (Thermo Fisher Scientific) using Lipofectamine™ RNAiMAX Transfection Reagent (Thermo Fisher Scientific) according to the manufacturer’s protocol and incubated for 5 min at room temperature. The medium was changed to full medium after 6 h and cells were reseeded for experiments 48 h post-transfection.

### Western blotting

For general cell lysis, RIPA lysis buffer (50 mM Tris-HCl pH 8, 150 mM NaCl, 1% Nonidet P-40 (NP-40), 2 mM MgCl_2_, 0.5% sodium deoxycholate) supplemented with protease inhibitor complex (PIC) (Roche, Grenzach, Germany), 0.1% SDS, 1 mM sodium orthovanadate, 5 mM sodium fluoride, 1 mM β-glycerophosphate, 1 mM phenylmethylsulfonyl fluoride and Pierce Universal Nuclease (Thermo Fisher Scientific) were used. Indicated cell lines were incubated with lysis buffer for 30 min on ice. The lysates were cleared by centrifugation at 18,000x *g* and 4 °C for 20 min and protein concentration was determined using the BCA Protein Assay Kit from Pierce™ (Thermo Fisher Scientific) according to the manufacturer’s protocol. Approximately 30-50 µg protein were boiled in 6x SDS loading buffer (350 mM Tris Base pH 6.8, 38% glycerol, 10% SDS, 93 mg/ml dithiothreitol (DTT), 120 mg/ml bromophenol blue) followed by Western blot analysis. For Western blot analysis of MLKL oligomers, indicated cell lines were lysed as described above in 1% NP-40 lysis buffer (20 mM Tris-HCl pH 7.5, 50 mM NaCl, 5 mM EDTA, 10% glycerol, 1% NP-40) supplemented with PIC, 1 mM sodium orthovanadate, 5 mM sodium fluoride, 1 mM β-glycerophosphate and Pierce Universal Nuclease. The cleared lysates were boiled in 6x SDS loading buffer or non-reducing 6x SDS loading buffer (350 mM Tris Base pH 6.8, 38% glycerol, 10% SDS, 120 mg/ml bromophenol blue), followed by Western blot analysis. The following antibodies were used in this study: human anti-M1 Ub (Genentech, San Francisco, CA, USA), rabbit anti-RNF31/HOIP (ab46322, Abcam, Cambridge, UK), mouse anti-HOIL-1 (MABC576, Merck KGaA, Darmstadt, Germany), rabbit anti-Sharpin (12541 S, Cell Signaling Technology, Danvers, MA, USA), mouse anti-phospho-IκBα (S32/S36) (9246 L, Cell Signaling Technology), rabbit anti-IκBα (9242 S, Cell Signaling Technology), rabbit anti-phospho-RIPK1 (S166) (65746 S, Cell Signaling Technology), rabbit anti-phospho-RIPK1 (S166) (31122 S, Cell Signaling Technology), mouse anti-RIPK1 (551041, BD Biosciences, San Jose, CA, USA), mouse anti-RIPK1 (610459, BD Biosciences), rabbit anti-phospho-RIPK3 (S277) (ab209384, Abcam), rabbit anti-RIPK3 (13526 S, Cell Signaling Technology), rabbit anti-phospho-MLKL (S358) (91689 S, Cell Signaling Technology), rabbit anti-phospho-MLKL (S345) (ab196436, Abcam), rabbit anti-MLKL (14993 S, Cell Signaling Technology), rabbit anti-MLKL (orb32399, Biorbyt Ltd., Cambridge, UK), mouse anti-ALIX (634502, BioLegend, San Diego, CA, USA), mouse anti-CD9 (sc-13118, Santa Cruz Biotechnologies, Santa Cruz, CA, USA), mouse anti-Flotillin-1 (610820, BD Biosciences), mouse anti-Flotillin-2 (610383, BD Biosciences), rabbit anti-GFP (632592, Clontech), mouse anti-GAPDH (5G4cc, HyTest Ltd, Turku, Finland), mouse anti-Vinculin (V9131, Merck KGaA), mouse anti-β-actin (A5441, Merck KGaA). The following horseradish peroxidase (HRP)-coupled secondary antibodies were used for detection using Pierce™ ECL Western Blotting-Substrate (Thermo Fisher Scientific): HRP-conjugated goat anti-mouse IgG (ab6789, Abcam), HRP-conjugated goat anti-rabbit IgG (ab6721, Abcam), HRP-conjugated goat anti-human IgG (AP309P, Merck KGaA). For stripping and re-probing of Western blot membranes, membranes were incubated with 0.4 M NaOH solution followed by blocking for 1 h. Representative blots of at least two independent experiments are shown. If the samples of one experiment are detected on multiple Western blotting membranes, only one representative loading control is shown for clarity. Original Western blots for all relevant figures are shown in “Supplementary Material - Original Blots”.

### RIPK3 immunoprecipitation

For RIPK3 immunoprecipitations, HT-29 RIPK3 KO cells re-expressing PAM-mutated Dox-inducible Strep-tagged RIPK3 WT [88] were seeded in full medium and RIPK3 expression was induced by adding 1 µg/mL Dox 6 h after seeding. Prior to treatment, the medium was changed to fresh medium and the cells were lysed in 1% NP-40 lysis buffer after treatment (as described above). For Strep-RIPK3 immunoprecipitations, MagStrep “type3” XT beads (IBA Lifesciences GmbH, Göttingen, Germany) were pre-washed twice in 1% NP-40 lysis buffer and incubated with 1,500-1,800 µg protein overnight at 4 °C on a rotating wheel. On the next day, the beads were washed five times with TBS-T (20 mM Tris, 150 mM NaCl, 0.1%Tween 20, pH 8.0) using a magnetic separator and then boiled in 2x SDS loading buffer for 5 min at 96 °C followed by Western blotting.

### Anti-GFP immunoprecipitations

Indicated cells were transfected with GFP only (pEGFP-N3, Clontech) or GFP-tagged FLOT2 (GFP-Reggie-1, kind gift of Ritva Tikkanen, JLU Gießen, Germany) using Lipofectamine 2000 (11668019, Thermo Fisher Scientific) according to the manufacturer’s recommendation. 24 h post-transfection, cells were reseeded, treated and lysed in 0.5% NP-40 Buffer (10 mM Tris/Cl pH 7.5, 150 mM NaCl, 1% Nonidet P40), supplemented with protease inhibitor cocktail, 1 mM PMSF and 1% SDS. Lysates were sonificated twice for 10 s, incubated on ice for 30 min and cleared by centrifugation for 10 min at 14,000 rpm. GFP-Trap magnetic beads (M-270, Chromotek) were rinsed 3 times with wash buffer (10 mM Tris/Cl pH 7.5, 150 mM NaCl, 0.05% Nonidet P40) and dilution buffer (10 mM Tris/Cl pH 7.5, 150 mM NaCl). Lysates were diluted to 0.1% SDS and 1500-1800 µg total protein were incubated with the beads for 1 h on a rotating wheel at 4 °C. The beads were washed five times with wash buffer and boiled in 2x SDS loading buffer supplemented with 10% β-mercaptoethanol at 96 °C for 5 min, followed by SDS-PAGE and Western blotting.

### MLKL-Strep immunoprecipitation

HT-29 MLKL CRISPR/Cas9 KO cells were stably reconstituted with Doxycycline (Dox)-inducible C-terminally Strep-tagged MLKL using Sleeping Beauty transposon-mediated expression using standard procedures [92]. To induce MLKL-Strep expression, cells were incubated for 3 h with 0.1 µg/mL Dox, followed by the indicated treatments and cell lysis in 1% NP-40 lysis buffer (20 mM Tris-HCl pH 7.5, 50 mM NaCl, 5 mM EDTA, 10% glycerol, 1% NP-40), supplemented with cOmplete™ PIC and phosphatase inhibitors (1 mM sodium orthovanadate, 2 mM β-glycerophosphate, 5 mM sodium fluoride) and Pierce™ Universal Nuclease and 1% SDS. MLKL-Strep was enriched using MagStrep “type3” XT beads (IBA Lifesciences GmbH, Göttingen, Germany) and analyzed by Western blotting.

### UBAN pulldowns

M1 ubiquitinated proteins were enriched using GST only or GST-tagged UBAN-coupled Glutathione MagBeads (Genscript, Piscataway NJ, SA), as described previously [88]. Briefly, cell lysates were prepared in 1% NP-40 lysis buffer as described above. For denaturing samples, lysates were boiled at 96 °C for 15 min. 1800–2000 µg protein were incubated with NP-40 lysis buffer pre-washed GST and GST-UBAN beads overnight on a rotating wheel at 4 °C. On the following day, the beads were washed five times with TBS-T using a magnetic separator, then boiled in 2x SDS loading buffer for 5 min at 96 °C and analyzed by Western blotting. Equal loading of the GST or GST-UBAN beads was confirmed using ponceau S (Merck KGaA) staining.

### Isolation of exosomes

For the enrichment of exosomes, the indicated HT-29 cell lines were seeded in full medium the day before treatment. Prior to the addition of compounds, the medium was removed, cells were washed with PBS and fresh FBS-free medium was added. After treatment, the cell culture supernatant was collected for exosome isolation and cells were lysed in RIPA lysis buffer as described above. The supernatant was cleared by centrifugation (2000x *g*, 30 min 4 °C) in order to remove cell debris. Exosome isolation was performed using the Total Exosome Isolation Reagent (from cell culture media) (Thermo Fisher Scientific) according to the manufacturer’s instructions. The cleared lysates were mixed with 0.5 volumes of the Total Exosome Isolation reagent and incubated overnight at 4 °C. The next day, the samples were centrifuged for 1 h at 10,000x *g* at 4 °C. The supernatants were discarded and the pelleted exosomes were lysed in RIPA lysis buffer, followed by incubation in an appropriate amount of 6x SDS loading buffer at 96 °C for 5 min. Exosome lysates and cell lysates were analyzed by Western blotting.

### Cell fractionation

Cell fractionation by phase separation using Triton X-114 lysis buffer was performed as described before [17]. In brief, indicated cell lines were treated and lysed in Triton-X114 (MilliporeSigma) lysis buffer (150 mM NaCl, 20 mM HEPES pH 7.4, 2% Triton X-114), supplemented with PIC, 1 mM sodium orthovanadate, 5 mM sodium fluoride and 1 mM β-glycerophosphate, for 30 min on ice, followed by 10 min centrifugation at 15,000 x *g* at 4 °C. 40-50 µg of the cleared lysate was incubated with 6x SDS loading dye and boiled for 5 min at 96 °C (input). The remaining lysate was warmed at 30 °C for 4 min and centrifuged at 1,500 x *g* for 5 min. The aqueous layer of the two-phase solution was collected and re-centrifuged at 1500 x *g* for 5 min to remove contaminations from the detergent-enriched phase. The detergent-enriched fraction was washed twice with basal buffer (150 mM NaCl, 20 mM HEPES pH 7.4) by centrifugation at 1500 x *g* for 5 min and then diluted in basal buffer. The protein concentrations of both the aqueous and detergent-enriched fractions were determined and 40-50 µg of protein were incubated in an appropriate amount of 6x SDS loading buffer at 96 °C for 5 min, followed by Western blotting.

### RNA isolation, cDNA synthesis and quantitative real-time PCR

Total RNA was prepared using the peqGOLD total RNA isolation kit (VWR, Radnor, PA, USA) according to manufacturer’s protocol. In brief, cell lysates were prepared using RNA lysis buffer, transferred to peqGOLD RNA Homogenizer Columns and centrifuged at 13,000 x *g* for 1 min. The filtrates were mixed with an equal volume of 70% ethanol and loaded to a peqGOLD RNA Mini Column followed by centrifugation at 10,000 x*g* for 1 min. After a washing step with RNA Wash Buffer I and two washing steps with 80% ethanol (centrifugation at 10,000 x *g*), the columns were dried by centrifugation at 12,000 x *g* for 2 min. The RNA was eluted in nuclease-free water by centrifugation at 12,000 x *g* for 2 min. For cDNA synthesis, 1 µg of RNA was transcribed using the RevertAid H Minus First Strand Kit (ThermoFisher Scientific) according to the manufacturer’s protocol. CXCL1, CXCL10, TNFα and ICAM1 gene expression levels were determined using SYBR green-based quantitative real-time PCR (Applied Biosystems, Darmstadt, Germany) using the 7900GR Fast Real-time PCR system (Applied Biosystems). Data were normalized against 18S-rRNA and RPII expression and relative gene expression levels were calculated using the ΔΔCt-method. The primers used in this study are listed below and were obtained from Eurofins (Hamburg, Germany). CXCL1: forward AACCGAAGTCATAGCCACAC, reverse GTTGGATTTGTCACTGTTCAGC. CXCL10: forward CTGAGCCTACAGCAGAGGAAC, reverse GATGCAGGTACAGCGTACAGT. TNFα: forward ACAACCCTCAGACGCCACAT, reverse TCCTTTCCAGGGGAGAGAGG. ICAM1: forward CTTCCTCACCGTGTACTGGAC, reverse GGCAGCGTAGGGTAAGGTTC. 18S-rRNA: forward CGCAAATTACCCACTCCCG, reverse TTCCAATTACAGGGCCTCGAA, RPII: forward GCACCACGTCCAATGACAT, reverse GTGCGGCTGCTTCCATAA.

### Quantification of CXCL1 cytokine secretion

To determine CXCL1 secretion from necroptotic cells, the indicated cell lines were seeded in 96-well dishes and treated as described above. After treatment, supernatants were collected and cleared by centrifugation (300 x *g*, 5 min, 4 °C). CXCL1 levels were measured after 3 h and 6 h using the Human CXCL1/GROα DuoSet® ELISA kit (R&D Systems, Minneapolis, MN, USA) according to the manufacturer’s instructions. Absorbance at 450 nm was determined using the Infinite M200 microplate reader (Tecan, Crailsheim, Germany).

### Immunofluorescence staining

For immunofluorescence staining of phosphorylated MLKL, indicated cell lines were seeded in CELLSTAR® 96-well tissue culture plates (Greiner Bio-One GmbH) at an appropriate density the day before treatment. After treatment, the supernatant was removed and the cells were fixed by incubation in 4% paraformaldehyde solution for 20 min at room temperature. Before permeabilization, cells were washed once with PBS and incubated with 2 μL of biotinylated wheat germ agglutinin (WGA) (Sigma, L5142) in PBS for 10 min. WGA was removed and the cells were washed once with PBS, followed by permeabilization for 15 min with 0.1% Triton X-100. After three washing steps with PBS, the cells were blocked in antibody dilution buffer (ADB) (0.9% NaCl, 10 mM Tris/HCl pH 7.5, 5 mM EDTA, 1 mg/mL BSA) for 45 min, followed by incubation with rabbit antiphospho-MLKL (S358) (1:100, ab187091, Abcam) or human anti-M1 Ub (Genentech, San Francisco, CA, USA) (diluted in ADB) overnight at 4 °C. On the next day, the cells were washed five times with PBS and incubated with Cy3 AffiniPure Donkey Anti-Rabbit IgG (H + L) (1:800, 711-165-152, Jackson Immuno Research, West Grove, PA, USA), goat anti-human AlexaFluor 647 (A-21445, ThermoFisher Scientific) and 0.1 µg/mL DAPI (diluted in ADB) for 90 min at room temperature. WGA was detected using 1 μg/mL DyLight650-conjugated streptavidin (ThermoFisher Scientific, 84547), diluted in ADB. After five washing steps with PBS, the cells were analyzed using the ImageXpress® Micro XLS Widefield High-Content Analysis System and MetaXpress® Software (Molecular Devices).

### Massive analysis of cDNA Ends (MACE-seq)

Massive Analyses of cDNA Ends (MACE-Seq) was performed by GenXPro GmbH in Frankfurt am Main using the MACE-Seq kit according to the manual of the manufacturers. Briefly, cDNA was generated from fragmented RNA with barcoded poly-A primers during reverse transcription. After second-strand synthesis and 5′ adapter integration, a PCR with the minimum number of cycles was used to produce a library that was sequenced on an Illumina NextSeq500 machine with 1×75 bps [93].

### MACE-seq analysis

Raw MACE-seq data were preprocessed using Cutadapt [94] to eliminate poly-A-tails as well as bad-quality base reads. FastQC was used to assess the quality of sequencing after trimming. Cleaned reads were mapped and quantified with a reference genome using STAR [95]. ENSEMBL-GTF data were used to provide genomic locations for quantification as well as additional data for annotation (such as gene name, gene description, Gene Ontology (GO)-Terms etc). Differential expression analysis was performed using DESeq2 [96], which is based on negative binomial generalized linear models. Results were compiled into a final table including significance parameters (*p* value, FDR) and log2FoldChanges. BigWig track files were generated by deepTools [97]. Final data visualization of the significantly expressed and the differentially regulated genes was performed by custom R-Scripts.

### Statistical analysis

Statistical significance was determined with the GraphPad Prism 9 Software (GraphPad Software, La Jolla CA, USA) using 2-way ANOVA followed by Tukey’s or Sidak’s multiple comparisons test. *P* values < 0.05 are considered significant and depicted as follows: *P* ≤ 0.05: *, *P* ≤ 0.01: **, *P* ≤ 0.001: ***, *P* ≤ 0.0001: ****, ns: not significant.

### Supplementary information

Supplementary Figures

Original Data File

Movie 1

Movie 2

AJ Checklist

## Data Availability

All data generated or analyzed during this study are included in this published article and its supplementary information files. MACE-seq data have been deposited at GEO and will be publicly available as of the date of publication. All primary data will be shared by the lead contact upon request.

## References

[1] Zhu K, Liang W, Ma Z, Xu D, Cao S, Lu X (2018). Necroptosis promotes cell-autonomous activation of proinflammatory cytokine gene expression. Cell Death Dis.

[2] Kaczmarek A, Vandenabeele P, Krysko DV (2013). Necroptosis: the release of damage-associated molecular patterns and its physiological relevance. Immunity.

[3] Weinlich R, Oberst A, Beere HM, Green DR (2017). Necroptosis in development, inflammation and disease. Nat Rev Mol Cell Biol.

[4] Pasparakis M, Vandenabeele P (2015). Necroptosis and its role in inflammation. Nature.

[5] Emmerich CH, Bakshi S, Kelsall IR, Ortiz-Guerrero J, Shpiro N, Cohen P (2016). Lys63/Met1-hybrid ubiquitin chains are commonly formed during the activation of innate immune signalling. Biochem Biophys Res Commun.

[6] Emmerich CH, Ordureau A, Strickson S, Arthur JS, Pedrioli PG, Komander D (2013). Activation of the canonical IKK complex by K63/M1-linked hybrid ubiquitin chains. Proc Natl Acad Sci USA.

[7] Bertrand MJ, Milutinovic S, Dickson KM, Ho WC, Boudreault A, Durkin J (2008). cIAP1 and cIAP2 facilitate cancer cell survival by functioning as E3 ligases that promote RIP1 ubiquitination. Mol Cell.

[8] Draber P, Kupka S, Reichert M, Draberova H, Lafont E, de Miguel D (2015). LUBAC-Recruited CYLD and A20 Regulate Gene Activation and Cell Death by Exerting Opposing Effects on Linear Ubiquitin in Signaling Complexes. Cell Rep.

[9] Wertz IE, Newton K, Seshasayee D, Kusam S, Lam C, Zhang J (2015). Phosphorylation and linear ubiquitin direct A20 inhibition of inflammation. Nature.

[10] Mompean M, Li W, Li J, Laage S, Siemer AB, Bozkurt G (2018). The Structure of the Necrosome RIPK1-RIPK3 Core, a Human Hetero-Amyloid Signaling Complex. Cell.

[11] Li J, McQuade T, Siemer AB, Napetschnig J, Moriwaki K, Hsiao YS (2012). The RIP1/RIP3 necrosome forms a functional amyloid signaling complex required for programmed necrosis. Cell.

[12] Cho YS, Challa S, Moquin D, Genga R, Ray TD, Guildford M (2009). Phosphorylation-driven assembly of the RIP1-RIP3 complex regulates programmed necrosis and virus-induced inflammation. Cell.

[13] He S, Wang L, Miao L, Wang T, Du F, Zhao L (2009). Receptor interacting protein kinase-3 determines cellular necrotic response to TNF-alpha. Cell.

[14] Sun L, Wang H, Wang Z, He S, Chen S, Liao D (2012). Mixed lineage kinase domain-like protein mediates necrosis signaling downstream of RIP3 kinase. Cell.

[15] Murphy JM, Czabotar PE, Hildebrand JM, Lucet IS, Zhang JG, Alvarez-Diaz S (2013). The pseudokinase MLKL mediates necroptosis via a molecular switch mechanism. Immunity.

[16] Su L, Quade B, Wang H, Sun L, Wang X, Rizo J (2014). A plug release mechanism for membrane permeation by MLKL. Structure.

[17] Wang H, Sun L, Su L, Rizo J, Liu L, Wang LF (2014). Mixed lineage kinase domain-like protein MLKL causes necrotic membrane disruption upon phosphorylation by RIP3. Mol Cell.

[18] Chen X, Li W, Ren J, Huang D, He WT, Song Y (2014). Translocation of mixed lineage kinase domain-like protein to plasma membrane leads to necrotic cell death. Cell Res.

[19] Hildebrand JM, Tanzer MC, Lucet IS, Young SN, Spall SK, Sharma P (2014). Activation of the pseudokinase MLKL unleashes the four-helix bundle domain to induce membrane localization and necroptotic cell death. Proc Natl Acad Sci USA.

[20] Dondelinger Y, Declercq W, Montessuit S, Roelandt R, Goncalves A, Bruggeman I (2014). MLKL compromises plasma membrane integrity by binding to phosphatidylinositol phosphates. Cell Rep.

[21] Samson AL, Zhang Y, Geoghegan ND, Gavin XJ, Davies KA, Mlodzianoski MJ (2020). MLKL trafficking and accumulation at the plasma membrane control the kinetics and threshold for necroptosis. Nat Commun.

[22] Gong YN, Guy C, Olauson H, Becker JU, Yang M, Fitzgerald P (2017). ESCRT-III Acts Downstream of MLKL to Regulate Necroptotic Cell Death and Its Consequences. Cell.

[23] Cai Z, Jitkaew S, Zhao J, Chiang HC, Choksi S, Liu J (2014). Plasma membrane translocation of trimerized MLKL protein is required for TNF-induced necroptosis. Nat Cell Biol.

[24] Seo J, Nam YW, Kim S, Oh DB, Song J (2021). Necroptosis molecular mechanisms: Recent findings regarding novel necroptosis regulators. Exp Mol Med.

[25] Karlowitz R, van Wijk SJL Surviving death: emerging concepts of RIPK3 and MLKL ubiquitination in the regulation of necroptosis. FEBS J. (2021). 10.1111/febs.16255 34710282

[26] Samson AL, Garnish SE, Hildebrand JM, Murphy JM (2021). Location, location, location: A compartmentalized view of TNF-induced necroptotic signaling. Sci Signal.

[27] Akutsu M, Dikic I, Bremm A (2016). Ubiquitin chain diversity at a glance. J Cell Sci.

[28] Komander D, Rape M (2012). The ubiquitin code. Annu Rev Biochem.

[29] Swatek KN, Komander D (2016). Ubiquitin modifications. Cell Res.

[30] Gerlach B, Cordier SM, Schmukle AC, Emmerich CH, Rieser E, Haas TL (2011). Linear ubiquitination prevents inflammation and regulates immune signalling. Nature.

[31] Ikeda F, Deribe YL, Skanland SS, Stieglitz B, Grabbe C, Franz-Wachtel M (2011). SHARPIN forms a linear ubiquitin ligase complex regulating NF-kappaB activity and apoptosis. Nature.

[32] Tokunaga F, Nakagawa T, Nakahara M, Saeki Y, Taniguchi M, Sakata S (2011). SHARPIN is a component of the NF-kappaB-activating linear ubiquitin chain assembly complex. Nature.

[33] Kirisako T, Kamei K, Murata S, Kato M, Fukumoto H, Kanie M (2006). A ubiquitin ligase complex assembles linear polyubiquitin chains. EMBO J.

[34] Oikawa D, Sato Y, Ohtake F, Komakura K, Hanada K, Sugawara K (2020). Molecular bases for HOIPINs-mediated inhibition of LUBAC and innate immune responses. Commun Biol.

[35] Katsuya K, Oikawa D, Iio K, Obika S, Hori Y, Urashima T (2019). Small-molecule inhibitors of linear ubiquitin chain assembly complex (LUBAC), HOIPINs, suppress NF-kappaB signaling. Biochem Biophys Res Commun.

[36] Hrdinka M, Gyrd-Hansen M (2017). The Met1-Linked Ubiquitin Machinery: Emerging Themes of (De)regulation. Mol Cell.

[37] Hrdinka M, Fiil BK, Zucca M, Leske D, Bagola K, Yabal M (2016). CYLD Limits Lys63- and Met1-Linked Ubiquitin at Receptor Complexes to Regulate Innate Immune Signaling. Cell Rep.

[38] Boisson B, Laplantine E, Dobbs K, Cobat A, Tarantino N, Hazen M (2015). Human HOIP and LUBAC deficiency underlies autoinflammation, immunodeficiency, amylopectinosis, and lymphangiectasia. J Exp Med.

[39] Boisson B, Laplantine E, Prando C, Giliani S, Israelsson E, Xu Z (2012). Immunodeficiency, autoinflammation and amylopectinosis in humans with inherited HOIL-1 and LUBAC deficiency. Nat Immunol.

[40] Oda H, Manthiram K, Chavan PP, Nakabo S, Kuehn HS, Beck DB, et al. Human LUBAC deficiency leads to autoinflammation and immunodeficiency by dysregulation in TNF-mediated cell death. medRxiv. 2022.11.09.22281431 (2022).

[41] Tokunaga F, Sakata S, Saeki Y, Satomi Y, Kirisako T, Kamei K (2009). Involvement of linear polyubiquitylation of NEMO in NF-kappaB activation. Nat Cell Biol.

[42] Seymour RE, Hasham MG, Cox GA, Shultz LD, Hogenesch H, Roopenian DC (2007). Spontaneous mutations in the mouse Sharpin gene result in multiorgan inflammation, immune system dysregulation and dermatitis. Genes Immun.

[43] Rickard JA, Anderton H, Etemadi N, Nachbur U, Darding M, Peltzer N (2014). TNFR1-dependent cell death drives inflammation in Sharpin-deficient mice. Elife..

[44] Kumari S, Redouane Y, Lopez-Mosqueda J, Shiraishi R, Romanowska M, Lutzmayer S (2014). Sharpin prevents skin inflammation by inhibiting TNFR1-induced keratinocyte apoptosis. Elife..

[45] Peltzer N, Rieser E, Taraborrelli L, Draber P, Darding M, Pernaute B (2014). HOIP deficiency causes embryonic lethality by aberrant TNFR1-mediated endothelial cell death. Cell Rep.

[46] Shimizu Y, Peltzer N, Sevko A, Lafont E, Sarr A, Draberova H (2017). The Linear ubiquitin chain assembly complex acts as a liver tumor suppressor and inhibits hepatocyte apoptosis and hepatitis. Hepatology.

[47] Taraborrelli L, Peltzer N, Montinaro A, Kupka S, Rieser E, Hartwig T (2018). LUBAC prevents lethal dermatitis by inhibiting cell death induced by TNF, TRAIL and CD95L. Nat Commun.

[48] Katsuya K, Hori Y, Oikawa D, Yamamoto T, Umetani K, Urashima T (2018). High-Throughput Screening for Linear Ubiquitin Chain Assembly Complex (LUBAC) Selective Inhibitors Using Homogenous Time-Resolved Fluorescence (HTRF)-Based Assay System. SLAS Discov.

[49] Shimizu Y, Taraborrelli L, Walczak H (2015). Linear ubiquitination in immunity. Immunol Rev.

[50] Fan W, Guo J, Gao B, Zhang W, Ling L, Xu T (2019). Flotillin-mediated endocytosis and ALIX-syntenin-1-mediated exocytosis protect the cell membrane from damage caused by necroptosis. Sci Signal.

[51] Bickel PE, Scherer PE, Schnitzer JE, Oh P, Lisanti MP, Lodish HF (1997). Flotillin and epidermal surface antigen define a new family of caveolae-associated integral membrane proteins. J Biol Chem.

[52] Lang DM, Lommel S, Jung M, Ankerhold R, Petrausch B, Laessing U (1998). Identification of reggie-1 and reggie-2 as plasmamembrane-associated proteins which cocluster with activated GPI-anchored cell adhesion molecules in non-caveolar micropatches in neurons. J Neurobiol.

[53] Lingwood D, Simons K (2010). Lipid rafts as a membrane-organizing principle. Science.

[54] Sezgin E, Levental I, Mayor S, Eggeling C (2017). The mystery of membrane organization: composition, regulation and roles of lipid rafts. Nat Rev Mol Cell Biol.

[55] Morrow IC, Rea S, Martin S, Prior IA, Prohaska R, Hancock JF (2002). Flotillin-1/reggie-2 traffics to surface raft domains via a novel golgi-independent pathway. Identification of a novel membrane targeting domain and a role for palmitoylation. J Biol Chem.

[56] Solis GP, Hoegg M, Munderloh C, Schrock Y, Malaga-Trillo E, Rivera-Milla E (2007). Reggie/flotillin proteins are organized into stable tetramers in membrane microdomains. Biochem J.

[57] Frick M, Bright NA, Riento K, Bray A, Merrified C, Nichols BJ (2007). Coassembly of flotillins induces formation of membrane microdomains, membrane curvature, and vesicle budding. Curr Biol.

[58] Glebov OO, Bright NA, Nichols BJ (2006). Flotillin-1 defines a clathrin-independent endocytic pathway in mammalian cells. Nat Cell Biol.

[59] Langhorst MF, Solis GP, Hannbeck S, Plattner H, Stuermer CA (2007). Linking membrane microdomains to the cytoskeleton: regulation of the lateral mobility of reggie-1/flotillin-2 by interaction with actin. FEBS Lett.

[60] Rossy J, Schlicht D, Engelhardt B, Niggli V (2009). Flotillins interact with PSGL-1 in neutrophils and, upon stimulation, rapidly organize into membrane domains subsequently accumulating in the uropod. PLoS One.

[61] Affentranger S, Martinelli S, Hahn J, Rossy J, Niggli V (2011). Dynamic reorganization of flotillins in chemokine-stimulated human T-lymphocytes. BMC Cell Biol.

[62] Solis GP, Schrock Y, Hulsbusch N, Wiechers M, Plattner H, Stuermer CA (2012). Reggies/flotillins regulate E-cadherin-mediated cell contact formation by affecting EGFR trafficking. Mol Biol Cell.

[63] Guillaume E, Comunale F, Do Khoa N, Planchon D, Bodin S, Gauthier-Rouviere C (2013). Flotillin microdomains stabilize cadherins at cell-cell junctions. J Cell Sci.

[64] Amaddii M, Meister M, Banning A, Tomasovic A, Mooz J, Rajalingam K (2012). Flotillin-1/reggie-2 protein plays dual role in activation of receptor-tyrosine kinase/mitogen-activated protein kinase signaling. J Biol Chem.

[65] Pust S, Klokk TI, Musa N, Jenstad M, Risberg B, Erikstein B (2013). Flotillins as regulators of ErbB2 levels in breast cancer. Oncogene.

[66] Damgaard RB, Elliott PR, Swatek KN, Maher ER, Stepensky P, Elpeleg O (2019). OTULIN deficiency in ORAS causes cell type-specific LUBAC degradation, dysregulated TNF signalling and cell death. EMBO Mol Med.

[67] Damgaard RB, Walker JA, Marco-Casanova P, Morgan NV, Titheradge HL, Elliott PR (2016). The Deubiquitinase OTULIN Is an Essential Negative Regulator of Inflammation and Autoimmunity. Cell.

[68] Sakamoto H, Egashira S, Saito N, Kirisako T, Miller S, Sasaki Y (2015). Gliotoxin suppresses NF-kappaB activation by selectively inhibiting linear ubiquitin chain assembly complex (LUBAC). ACS Chem Biol.

[69] Peltzer N, Darding M, Montinaro A, Draber P, Draberova H, Kupka S (2018). LUBAC is essential for embryogenesis by preventing cell death and enabling haematopoiesis. Nature.

[70] Vanlangenakker N, Bertrand MJ, Bogaert P, Vandenabeele P, Vanden Berghe T (2011). TNF-induced necroptosis in L929 cells is tightly regulated by multiple TNFR1 complex I and II members. Cell Death Dis.

[71] Zargarian S, Shlomovitz I, Erlich Z, Hourizadeh A, Ofir-Birin Y, Croker BA (2017). Phosphatidylserine externalization, “necroptotic bodies” release, and phagocytosis during necroptosis. PLoS Biol.

[72] Yoon S, Kovalenko A, Bogdanov K, Wallach D (2017). MLKL, the Protein that Mediates Necroptosis, Also Regulates Endosomal Trafficking and Extracellular Vesicle Generation. Immunity.

[73] Boj SF, Hwang CI, Baker LA, Chio II, Engle DD, Corbo V (2015). Organoid models of human and mouse ductal pancreatic cancer. Cell.

[74] Broutier L, Andersson-Rolf A, Hindley CJ, Boj SF, Clevers H, Koo BK (2016). Culture and establishment of self-renewing human and mouse adult liver and pancreas 3D organoids and their genetic manipulation. Nat Protoc.

[75] Dossena M, Piras R, Cherubini A, Barilani M, Dugnani E, Salanitro F (2020). Standardized GMP-compliant scalable production of human pancreas organoids. Stem Cell Res Ther.

[76] Georgakopoulos N, Prior N, Angres B, Mastrogiovanni G, Cagan A, Harrison D (2020). Long-term expansion, genomic stability and in vivo safety of adult human pancreas organoids. BMC Dev Biol.

[77] Jung N, Moreth T, Stelzer EHK, Pampaloni F, Windbergs M (2021). Non-invasive analysis of pancreas organoids in synthetic hydrogels defines material-cell interactions and luminal composition. Biomater Sci.

[78] Petrie EJ, Sandow JJ, Jacobsen AV, Smith BJ, Griffin MDW, Lucet IS (2018). Conformational switching of the pseudokinase domain promotes human MLKL tetramerization and cell death by necroptosis. Nat Commun.

[79] Davies KA, Tanzer MC, Griffin MDW, Mok YF, Young SN, Qin R (2018). The brace helices of MLKL mediate interdomain communication and oligomerisation to regulate cell death by necroptosis. Cell Death Differ.

[80] Tanzer MC, Matti I, Hildebrand JM, Young SN, Wardak A, Tripaydonis A (2016). Evolutionary divergence of the necroptosis effector MLKL. Cell Death Differ.

[81] Petrie EJ, Czabotar PE, Murphy JM (2019). The Structural Basis of Necroptotic Cell Death Signaling. Trends Biochem Sci.

[82] Petrie EJ, Birkinshaw RW, Koide A, Denbaum E, Hildebrand JM, Garnish SE (2020). Identification of MLKL membrane translocation as a checkpoint in necroptotic cell death using Monobodies. Proc Natl Acad Sci USA.

[83] Meister M, Banfer S, Gartner U, Koskimies J, Amaddii M, Jacob R (2017). Regulation of cargo transfer between ESCRT-0 and ESCRT-I complexes by flotillin-1 during endosomal sorting of ubiquitinated cargo. Oncogenesis.

[84] Liu Z, Dagley LF, Shield-Artin K, Young SN, Bankovacki A, Wang X, et al. Oligomerization-driven MLKL ubiquitylation antagonises necroptosis. bioRxiv. 2021.05.01.442209 (2021). 10.15252/embj.2019103718 PMC8634140 34698396

[85] Garcia LR, Tenev T, Newman R, Haich RO, Liccardi G, John SW (2021). Ubiquitylation of MLKL at lysine 219 positively regulates necroptosis-induced tissue injury and pathogen clearance. Nat Commun.

[86] Akimov V, Barrio-Hernandez I, Hansen SVF, Hallenborg P, Pedersen AK, Bekker-Jensen DB (2018). UbiSite approach for comprehensive mapping of lysine and N-terminal ubiquitination sites. Nat Struct Mol Biol.

[87] van Wijk SJ, Fiskin E, Putyrski M, Pampaloni F, Hou J, Wild P (2012). Fluorescence-based sensors to monitor localization and functions of linear and K63-linked ubiquitin chains in cells. Mol Cell.

[88] Roedig J, Kowald L, Juretschke T, Karlowitz R, Ahangarian Abhari B, Roedig H (2021). USP22 controls necroptosis by regulating receptor-interacting protein kinase 3 ubiquitination. EMBO Rep.

[89] Doench JG, Fusi N, Sullender M, Hegde M, Vaimberg EW, Donovan KF (2016). Optimized sgRNA design to maximize activity and minimize off-target effects of CRISPR-Cas9. Nat Biotechnol.

[90] Sanjana NE, Shalem O, Zhang F (2014). Improved vectors and genome-wide libraries for CRISPR screening. Nat Methods.

[91] Hof L, Moreth T, Koch M, Liebisch T, Kurtz M, Tarnick J (2021). Long-term live imaging and multiscale analysis identify heterogeneity and core principles of epithelial organoid morphogenesis. BMC Biol.

[92] Kowarz E, Löscher D, Marschalek R (2015). Optimized Sleeping Beauty transposons rapidly generate stable transgenic cell lines. Biotechnology Journal.

[93] Muller S, Rycak L, Afonso-Grunz F, Winter P, Zawada AM, Damrath E (2014). APADB: a database for alternative polyadenylation and microRNA regulation events. Database (Oxford).

[94] Martin M Cutadapt removes adapter sequences from high-throughput sequencing reads. 2011;17:3.

[95] Dobin A, Davis CA, Schlesinger F, Drenkow J, Zaleski C, Jha S (2013). STAR: ultrafast universal RNA-seq aligner. Bioinformatics.

[96] Love MI, Huber W, Anders S (2014). Moderated estimation of fold change and dispersion for RNA-seq data with DESeq2. Genome Biol.

[97] Ramirez F, Ryan DP, Gruning B, Bhardwaj V, Kilpert F, Richter AS (2016). deepTools2: a next generation web server for deep-sequencing data analysis. Nucleic Acids Res.

