# Supplementary material for: LUBAC-mediated M1 Ub regulates necroptosis by segregating the cellular distribution of active MLKL
Source: Cell Death Dis. 2024 Jan 20;15(1):77. doi: 10.1038/s41419-024-06447-6 (PMC10799905; doi:10.1038/s41419-024-06447-6)
Supplement: Supplementary file 1 — Supplementary Figures [file 41419_2024_6447_MOESM1_ESM.pdf]

LUBAC-mediated M1 Ub regulates necroptosis by segregating the cellular distribution of active MLKL

Nadine Weinelt, Kaja Nicole Wächtershäuser, Gulustan Celik, Birte Jeiler, Isabelle Gollin, Laura Zein, Sonja Smith, Geoffroy Andrieux, Tonmoy Das, Jens Roedig, Leonard Feist, Björn Rotter, Melanie Boerries, Francesco Pampaloni, Sjoerd J. L. van Wijk

Supplementary Figures 1-7 and legends

Movie 1-2 legends

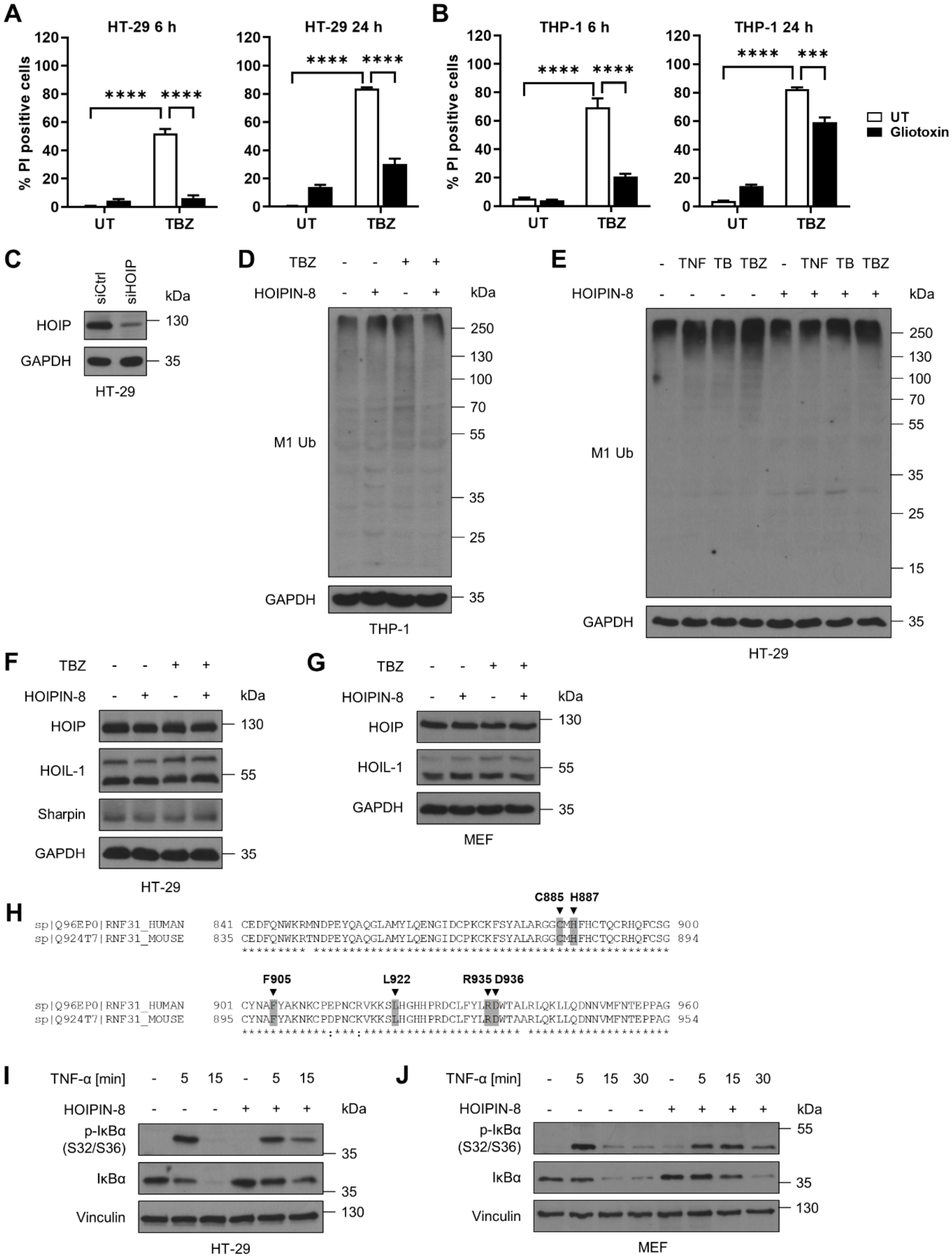
A
HT-29 6 h
HT-29 24 h
% PI positive cells
UT
TBZ
****
***
B
THP-1 6 h
THP-1 24 h
UT
Gliotoxin
C
siCtrl
siHOIP
kDa
HOIP
GAPDH
HT-29
D
TBZ
HOIPIN-8
M1 Ub
GAPDH
THP-1
E
TNF
TB
TBZ
HT-29
F
HOIP
HOIL-1
Sharpin
GAPDH
HT-29
G
MEF
H
C885 H887
sp|Q96EP0|RNF31_HUMAN 841 CEDFQNWKRMNDPEYQAQGLAMYLQENGIDCPKCKFSYALARGGCMHFHCTQCRHQFCSG 900
sp|Q924T7|RNF31_MOUSE 835 CEDFQNWKRTNDPEYQAQGLAMYLQENGIDCPKCKFSYALARGGCMHFHCTQCRHQFCSG 894
F905 L922 R935 D936
sp|Q96EP0|RNF31_HUMAN 901 CYNAFYAKNKCPEPNCRVKKSLHGHHPRDCLFYLRDWTALRLQKLLQDNNVMFNTEPPAG 960
sp|Q924T7|RNF31_MOUSE 895 CYNAFYAKNKCPDPNCKVKKSLHGHHPRDCLFYLRDWTAARLQKLLQDNNVMFNTEPPAG 954
I
TNF-α [min]
HOIPIN-8
p-IκBα (S32/S36)
IκBα
Vinculin
HT-29
J
MEF

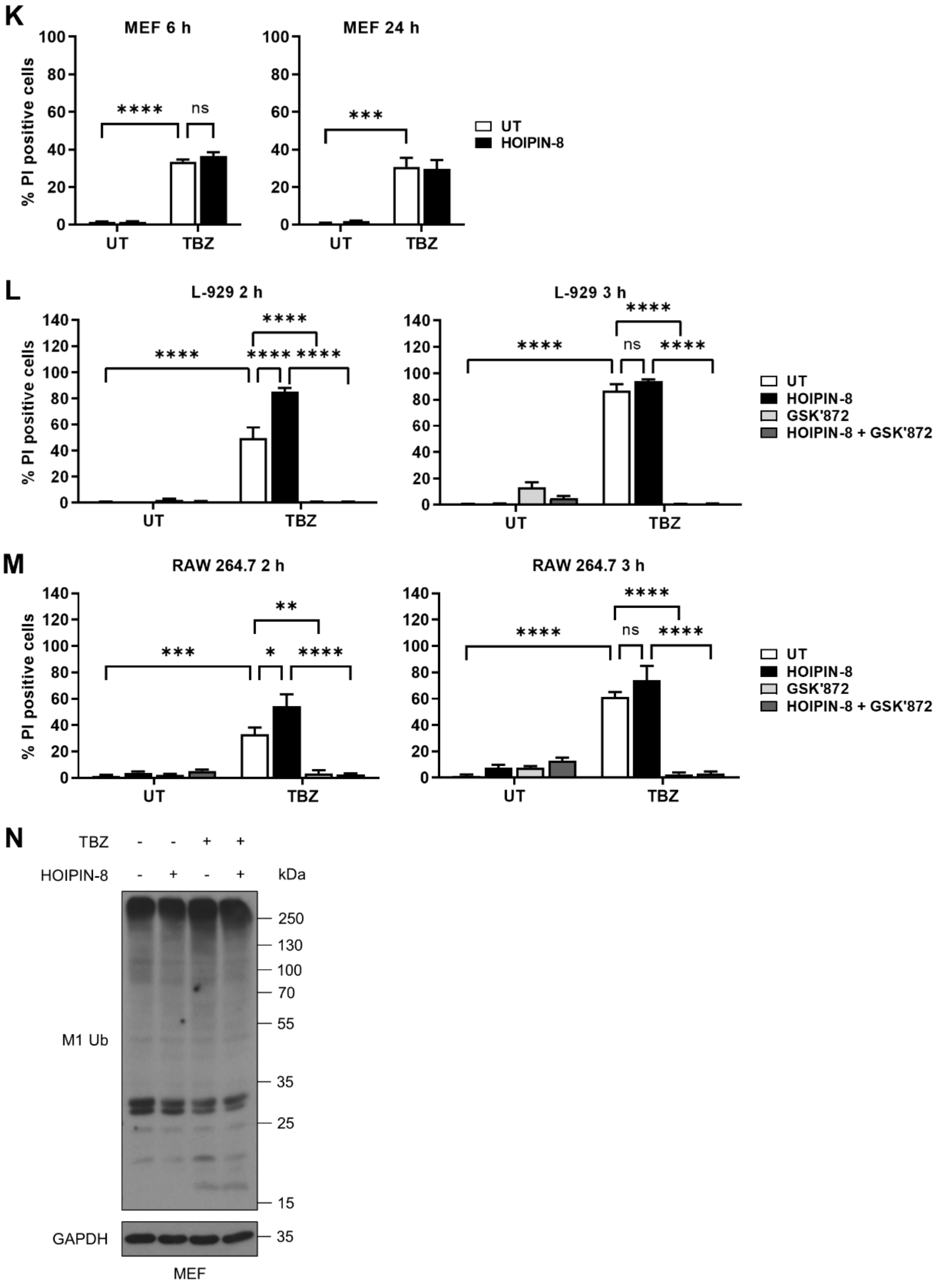

Figure S1. Related to Figure 1. A. Quantification of cell death in untreated (UT) and Gliotoxin (1 μM)-pre-treated HT-29 cells after treatment with TBZ (10 ng/mL TNFα, 1 μM BV6, 20 μM zVAD.fmk) for the indicated time points. Mean and SEM of *n*=3 independent experiments are shown. ****P<0.0001. **B.** Quantification of cell death in untreated (UT) and Gliotoxin (1 μM)-pre-treated THP-1 cells after treatment with TBZ (10 ng/mL TNFα, 1 μM BV6, 20 μM zVAD.fmk) for the indicated time points. Mean and

SEM of *n*=3 independent experiments are shown. ****P*<0.001; *****P*<0.0001. **C.** Western blot analysis of HOIP levels in control (siCtrl) and HOIP knockdown (siHOIP) HT-29 cells. GAPDH was used as loading control. Representative blots of at least two independent experiments are shown. Related to Figure 1C. **D.** Western blot analysis of total M1 poly-Ub levels in control or HOIPIN-8 (30 µM)-pre-treated THP-1 cells treated with TBZ (10 ng/mL TNFα, 1 µM BV6, 20 µM zVAD.fmk) for 4 h. GAPDH was used as loading control. Representative blots of at least two independent experiments are shown. **E.** Western blot analysis of total M1 poly-Ub levels in control or HOIPIN-8 (30 µM)-pre-treated HT-29 cells treated with TNFα (10 ng/mL), TB (10 ng/mL TNFα, 1 µM BV6) or TBZ (10 ng/mL TNFα, 1 µM BV6, 20 µM zVAD.fmk) for 4 h. GAPDH was used as loading control. Representative blots of at least two independent experiments are shown. **F.** Western blot analysis of LUBAC subunit (HOIP, HOIL-1 and Sharpin) expression levels in control *versus* HOIPIN-8 (30 µM)-pre-treated HT-29 cells upon treatment with TBZ (10 ng/mL TNFα, 1 µM BV6, 20 µM zVAD.fmk) for 4 h. GAPDH was used as loading control. Representative blots of at least two independent experiments are shown. **G.** Western blot analysis of LUBAC subunits HOIP and HOIL-1 expression levels in control *versus* HOIPIN-8 (30 µM)-pre-treated MEFs upon treatment with TBZ (10 ng/mL TNFα, 1 µM BV6, 20 µM zVAD.fmk) for 3 h. GAPDH was used as loading control. Representative blots of at least two independent experiments are shown. **H.** Amino acid sequence alignment showing conservation of human (aa841-aa960) and murine HOIP (RNF31) (aa835-aa954). Conserved residues interacting with HOIPIN-8 are highlighted and indicated by arrowheads. Sequence alignment was performed using the CLUSTAL O (1.2.4) algorithm. **I.** Western blot analysis of phosphorylated and total IκBα levels in control or HOIPIN-8 (30 µM)-pre-treated HT-29 cells upon treatment with 10 ng/mL TNFα for the indicated time points. Vinculin was used as loading control. Representative blots of at least two independent experiments are shown. **J.** Western blot analysis of phosphorylated and total IκBα levels in control or HOIPIN-8 (30 µM)-pre-treated MEFs upon treatment with 10 ng/mL TNFα for the indicated time. Vinculin was used as loading control. Representative blots of at least two independent experiments are shown. **K.** Quantification of cell death in untreated (UT) and HOIPIN-8 (30 µM) pre-treated MEFs after treatment with TBZ (10 ng/mL TNFα, 1 µM BV6, 20 µM zVAD.fmk) for the indicated time points. Mean and SEM of *n*=4 independent experiments are shown. ****P*<0.001; *****P*<0.0001; ns not significant. **L.** Quantification of cell death in untreated (UT) and HOIPIN-8 (30 µM)-pre-

treated, GSK’872 (20 µM)-pre-treated or HOIPIN-8 and GSK’872-pre-treated L-929 cells after treatment with TBZ (10 ng/mL TNFα, 1 µM BV6, 20 µM zVAD.fmk) for the indicated time points. Mean and SEM of *n*=3 independent experiments are shown. ****P<0.0001; ns not significant. **M.** Quantification of cell death in untreated (UT) and HOIPIN-8 (30 µM)-pre-treated, GSK’872 (20 µM)-pre-treated or HOIPIN-8 and GSK’872-pre-treated RAW 264.7 cells after treatment with TBZ (10 ng/mL TNFα, 1 µM BV6, 20 µM zVAD.fmk) for the indicated time points. Mean and SEM of *n*=3 independent experiments are shown. *P<0.05; **P<0.01; ***P<0.001; ****P<0.0001; ns not significant. **N.** Western blot analysis of total M1 poly-Ub levels in control or HOIPIN-8 (30 µM)-pre-treated MEFs treated with TBZ (10 ng/mL TNFα, 1 µM BV6, 20 µM zVAD.fmk) for 3 h. GAPDH was used as loading control. Representative blots of at least two independent experiments are shown. Statistical significance was determined using 2-way ANOVA followed by Tukey’s multiple comparisons test (**A**, **B**, **K**, **L**, **M**).

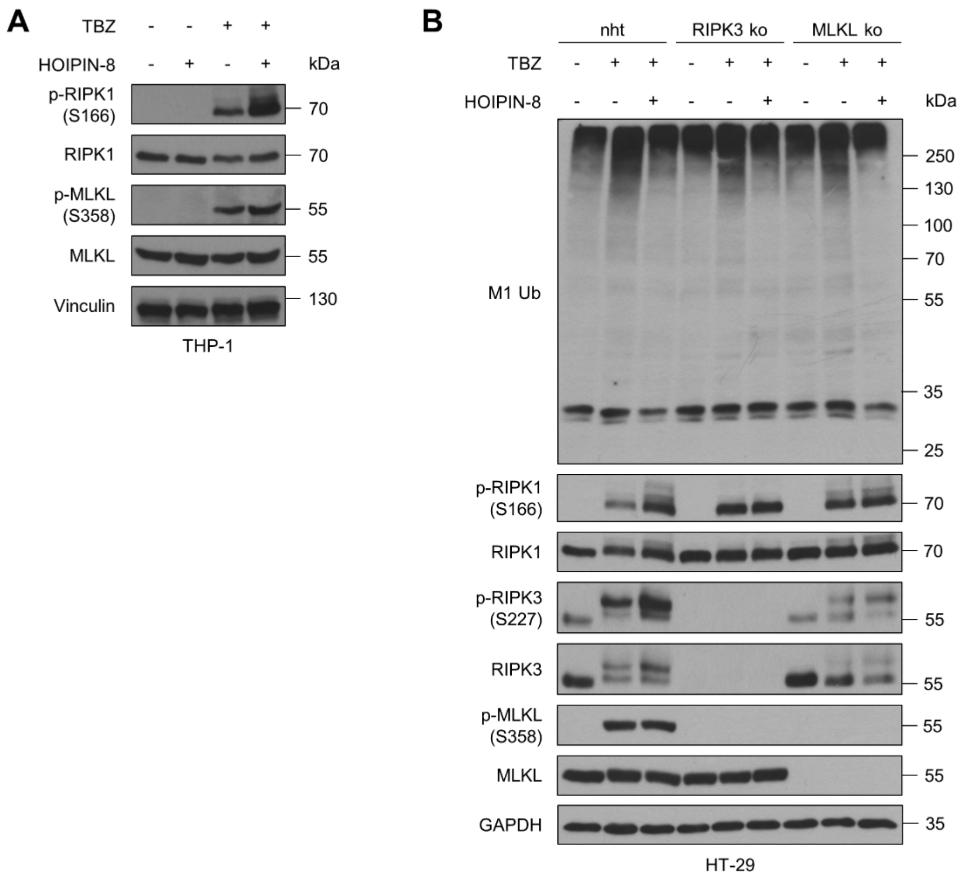

Figure S2. Related to Figure 2. A. Western blot analysis of expression levels of phosphorylated and total RIPK1 and MLKL upon 4 h TBZ (10 ng/mL TNFα, 1 μM BV6, 20 μM zVAD.fmk) treatment of THP-1 cells pre-treated with HOIPIN-8 (30 μM). Vinculin was used as loading control. Representative blots of at least two independent experiments are shown. **B.** Western blot analysis of expression levels of phosphorylated and total RIPK1, RIPK3 and MLKL, and of M1 poly-Ub in TBZ-treated (10 ng/mL TNFα, 1 μM BV6, 20 μM zVAD.fmk) control (nht), RIPK3 KO and MLKL KO HT-29 cells pre-treated with HOIPIN-8 (30 μM). GAPDH was used as loading control. Representative blots of at least two independent experiments are shown.

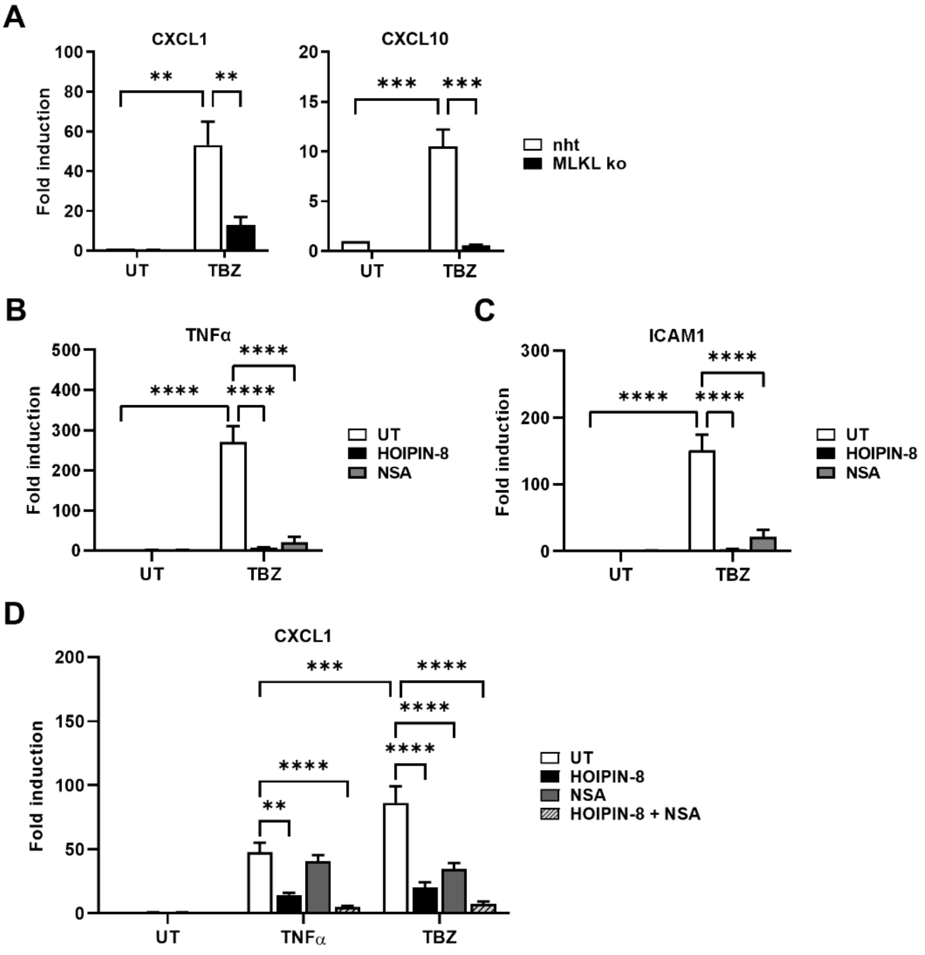

Figure S3. Related to Figure 3. A. mRNA expression levels of CXCL1 and CXCL10 of control (nht) or MLKL KO HT-29 cells treated with TBZ (10 ng/mL TNFα, 1 µM BV6, 20 µM zVAD.fmk) for 3 h. Gene expression was normalized against 18S and RPII mRNA expression and is presented as x-fold mRNA expression compared to the untreated (UT) control cells. Mean and SEM of *n*=3 independent experiments are shown. ***P*<0.01; ****P*<0.001. **B.** mRNA expression levels of TNFα of control (UT), HOIPIN-8 (30 µM) or NSA (10 µM)-pre-treated HT-29 cells upon treatment with TBZ (10 ng/mL TNFα, 1 µM BV6, 20 µM zVAD.fmk) for 3 h. Gene expression was normalized against 18S and RPII mRNA expression and is presented as x-fold mRNA expression compared to the untreated (UT) control. Mean and SEM of *n*=3 independent experiments are shown. *****P*<0.0001. **C.** mRNA expression levels of ICAM1 of control (UT), HOIPIN-8 (30 µM) or NSA (10 µM)-pre-treated HT-29 cells upon treatment with TBZ (10 ng/mL TNFα, 1 µM BV6, 20 µM zVAD.fmk) for 3 h. Gene expression was normalized against 18S and RPII mRNA expression and is presented as x-fold mRNA expression compared to the untreated (UT) control. Mean and SEM

of *n*=3 independent experiments are shown. ****$P<0.0001$. **D.** mRNA expression levels of CXCL1 of control (UT), HOIPIN-8 (30 µM), NSA (10 µM) or combined HOIPIN-8 (30 µM) and NSA (10 µM)-pre-treated HT-29 cells upon treatment with TNFα (10 ng/mL) or TBZ (10 ng/mL TNFα, 1 µM BV6, 20 µM zVAD.fmk) for 3 h. Gene expression was normalized against 18S and RPII mRNA expression and is presented as x-fold mRNA expression compared to the untreated (UT) control. Mean and SEM of *n*=4 independent experiments are shown. **$P<0.01$; ***$P<0.001$; ****$P<0.0001$. Statistical significance was determined using 2-way ANOVA followed by Tukey's multiple comparisons test.

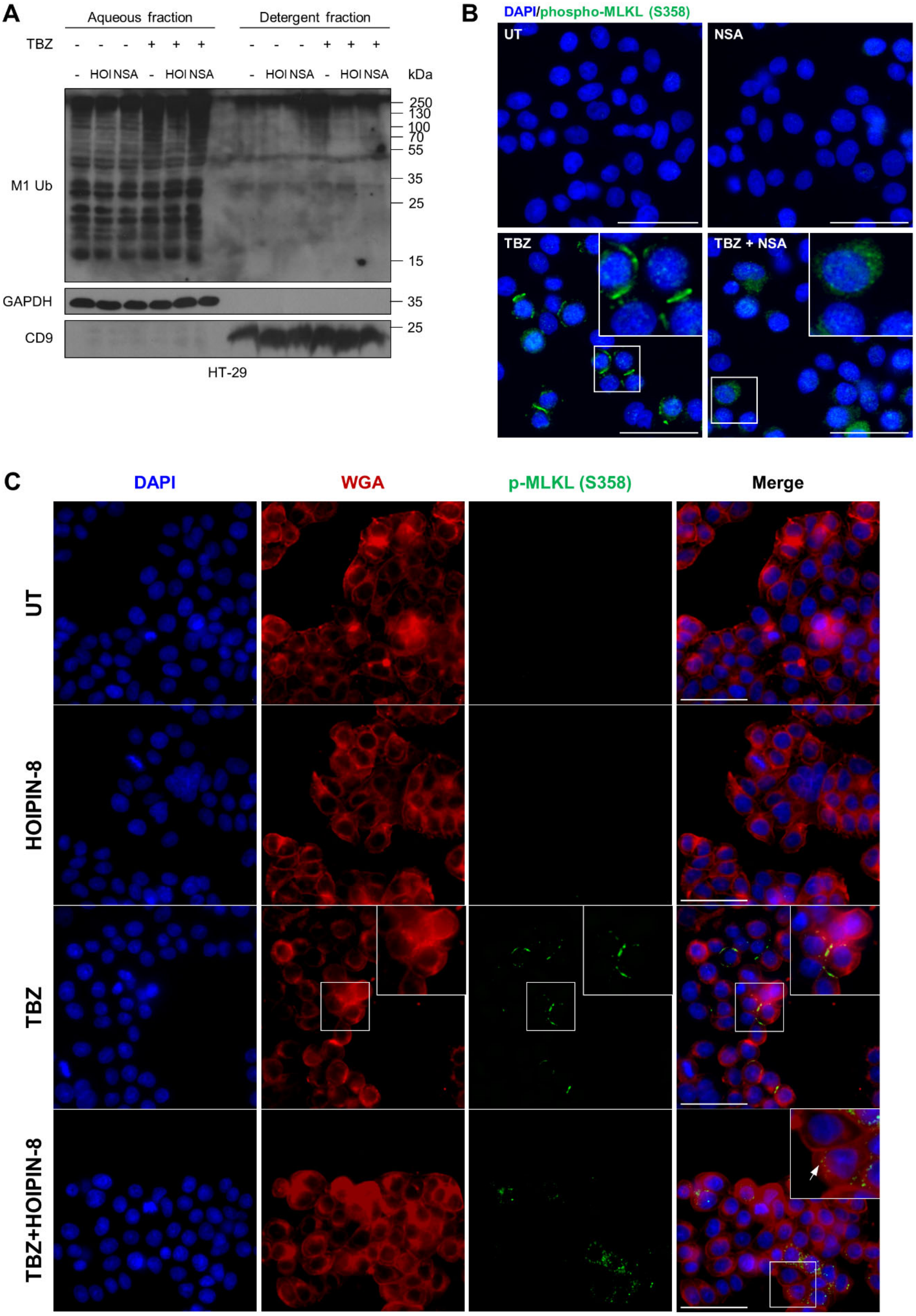
A
Aqueous fraction
Detergent fraction
TBZ
- - - + + + - - - + + +
- HOI NSA - HOI NSA - HOI NSA - HOI NSA
kDa
250
130
100
70
55
35
25
15
M1 Ub
GAPDH
35
CD9
25
HT-29
B
DAPI/phospho-MLKL (S358)
UT
NSA
TBZ
TBZ + NSA
C
DAPI
WGA
p-MLKL (S358)
Merge
UT
HOIPIN-8
TBZ
TBZ+HOIPIN-8

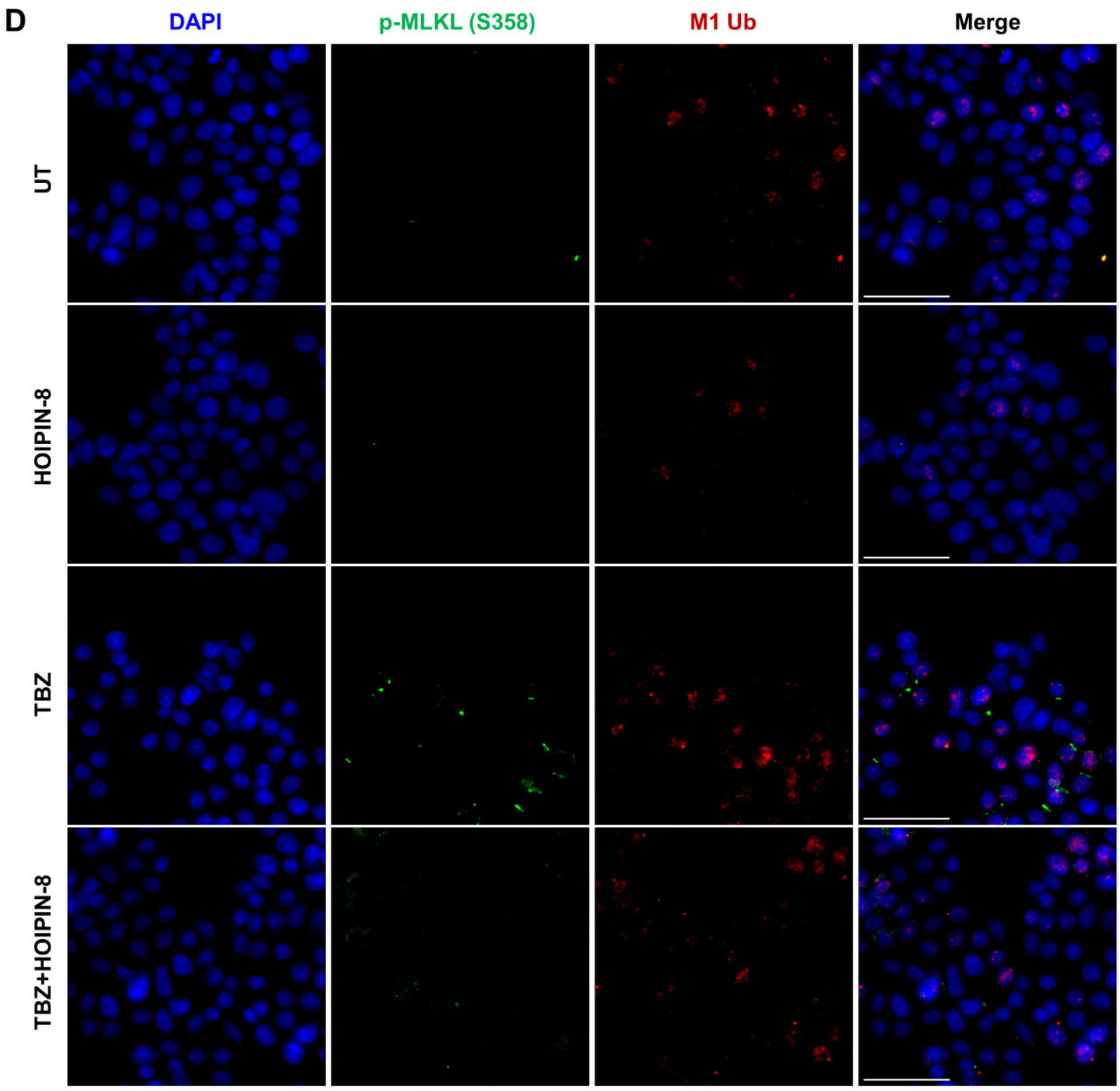

Figure S4. Related to Figure 4. A. Distribution of M1 poly-Ub in the micelle-poor (aqueous) and micelle-rich (detergent) fraction after phase separation using Triton X-114 lysis buffer in untreated (UT) and NSA (10 µM) or HOIPIN-8 (30 µM)-pre-treated HT-29 cells upon treatment with TBZ (10 ng/mL TNFα, 1 µM BV6, 20 µM zVAD.fmk) for 4 h. GAPDH was used as loading control for soluble proteins and CD9 as loading control for membrane proteins. Representative blots of at least two independent experiments are shown. **B.** Fluorescence microscopy images show localization of phosphorylated MLKL (green) in untreated (UT) and NSA (10 µM)-pre-treated HT-29 cells and after treatment with TBZ (10 ng/mL TNFα, 1 µM BV6, 20 µM zVAD.fmk) for 3 h. Nuclei were stained with DAPI (blue). Representative images of at least two independent experiments are shown. Scale bar 50 µm. **C.** Fluorescence microscopy images show localization of phosphorylated MLKL (green) and WGA-labelled plasma

membrane (red) in untreated (UT) and HOIPIN-8 (30 μM)-pre-treated HT-29 cells and after treatment with TBZ (10 ng/mL TNFα, 1 μM BV6, 20 μM zVAD.fmk) for 3 h. Nuclei were stained with DAPI (blue). Representative images of at least two independent experiments are shown. Scale bar 50 μm. **D.** Fluorescence microscopy images show localization of phosphorylated MLKL (green) and M1 poly-Ub (red) in untreated (UT) and HOIPIN-8 (30 μM)-pre-treated HT-29 cells and after treatment with TBZ (10 ng/mL TNFα, 1 μM BV6, 20 μM zVAD.fmk) for 3 h. Nuclei were stained with DAPI (blue). Representative images of at least two independent experiments are shown. Scale bar 50 μm.

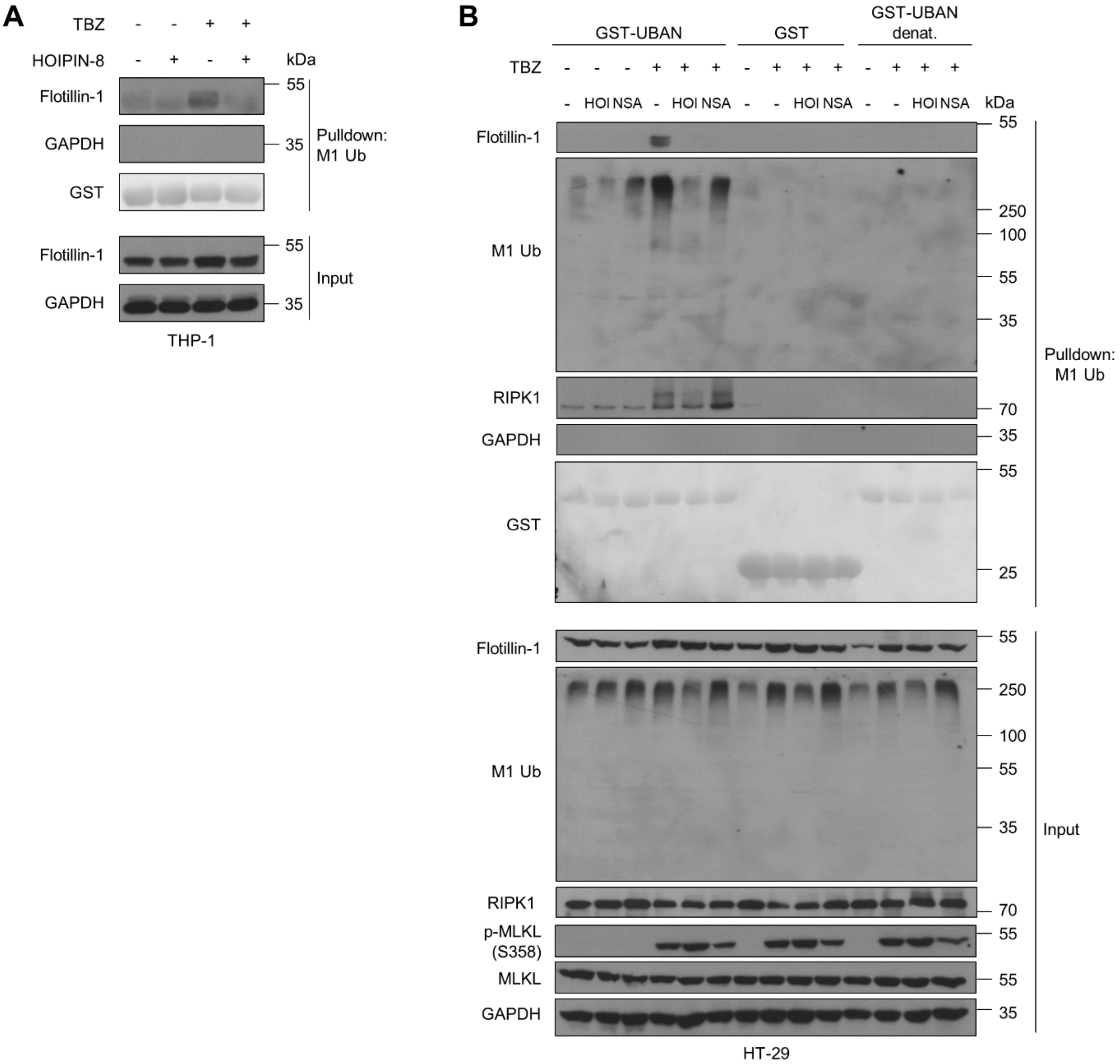

A
TBZ - - + +
HOIPIN-8 - + - +
kDa
Flotillin-1
55
GAPDH
35
GST
Pulldown: M1 Ub
Flotillin-1
55
GAPDH
35
Input
THP-1
B
GST-UBAN
GST
GST-UBAN denat.
TBZ - - - + + + - + + + - + + +
- HOI NSA - HOI NSA - - HOI NSA - - HOI NSA
kDa
Flotillin-1
55
M1 Ub
250
100
55
35
RIPK1
70
GAPDH
35
GST
55
25
Pulldown: M1 Ub
Flotillin-1
55
M1 Ub
250
100
55
35
RIPK1
70
p-MLKL (S358)
55
MLKL
55
GAPDH
35
Input
HT-29

C

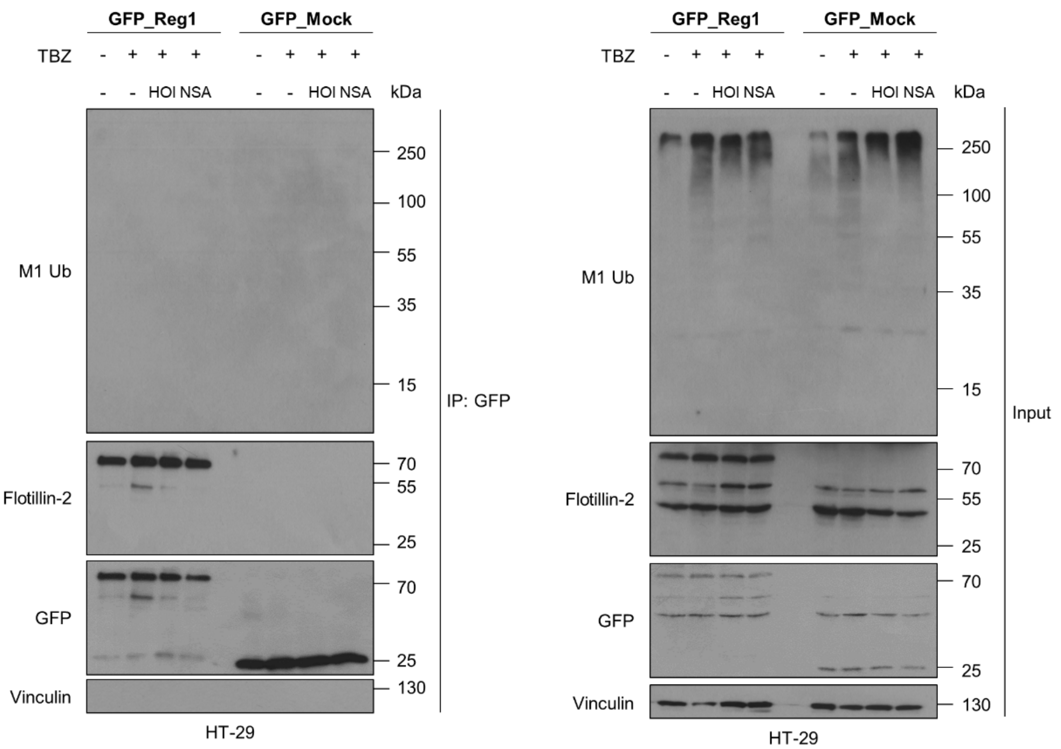

D

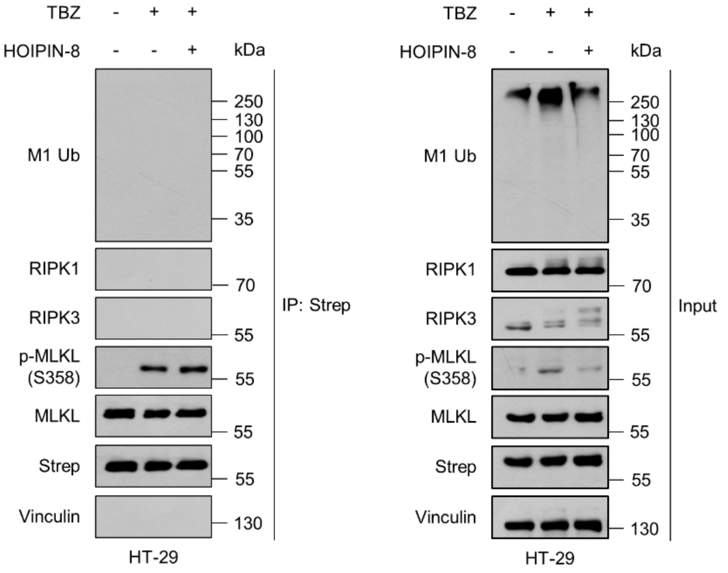

Figure S5. Related to Figure 5. A. GST-UBAN-mediated pulldown of M1 ubiquitinated proteins in control or HOIPIN-8 (30 μM)-pre-treated THP-1 cells treated with TBZ (10 ng/mL TNFα, 1 μM BV6, 20 μM zVAD.fmk) for 4 h. GST and GAPDH were used as loading controls. Representative blots of at least two independent experiments are shown. **B.** Pulldown of M1 ubiquitinated proteins in control, HOIPIN-8 (30 μM) or NSA (10 μM)-pre-treated HT-29 cells treated with TBZ (10 ng/mL TNFα, 1 μM BV6, 20 μM zVAD.fmk) for 4 h using GST-UBAN or control (GST) beads. For the denaturing conditions, lysates were boiled at 96°C for 15 min prior to the pulldown with GST-UBAN

beads. Ponceau and GAPDH were used as loading controls. Representative blots of at least two independent experiments are shown. **C.** Immunoprecipitation of GFP-tagged Flotillin-2 (GFP_Reg1) in control, HOIPIN-8 (30 μM) or NSA (10 μM)-pre-treated GFP-Reg1 and GFP-Mock transfected HT-29 cells treated with TBZ (10 ng/mL TNFα, 1 μM BV6, 20 μM zVAD.fmk) for 4 h. GFP and Vinculin were used as loading controls. Representative blots of at least two independent experiments are shown. **D.** Immunoprecipitation of Strep-MLKL from control and HOIPIN-8 (30 μM)-pre-treated MLKL KO HT-29 cells re-expressing PAM-mutated Dox-inducible Strep-tagged MLKL WT after treatment with TBZ (10 ng/mL TNFα, 1 μM BV6, 20 μM zVAD.fmk) for 4 h. Cells were incubated for 3 h with 0.1 μg/mL Dox prior to treatment to induce expression of Strep-MLKL. Vinculin was used as loading control. Representative blots of at least two independent experiments are shown.

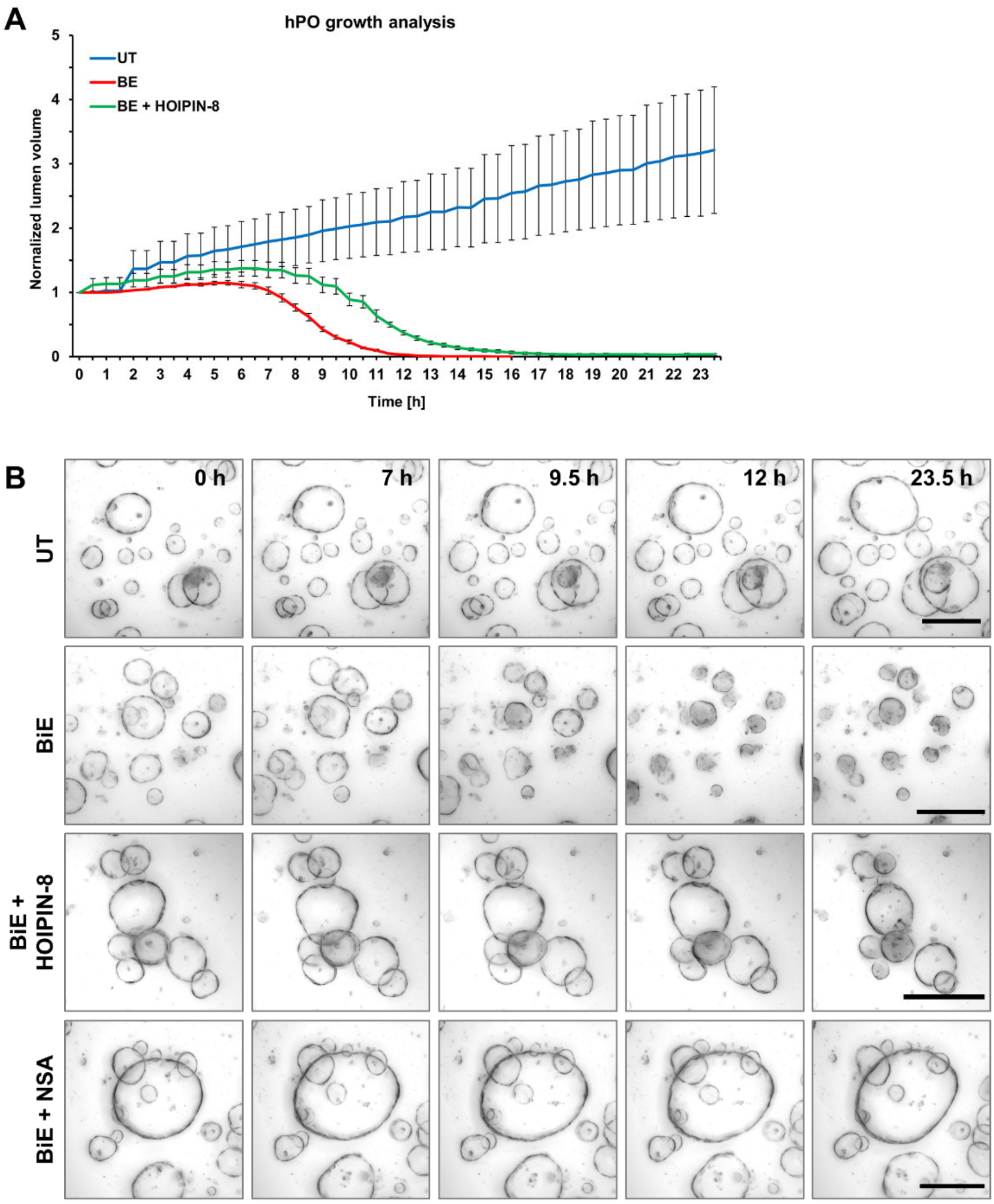
A
hPO growth analysis
UT
BE
BE + HOIPIN-8
Normalized lumen volume
0
1
2
3
4
5
Time [h]
0 1 2 3 4 5 6 7 8 9 10 11 12 13 14 15 16 17 18 19 20 21 22 23
B
0 h
7 h
9.5 h
12 h
23.5 h
UT
BiE
BiE + HOIPIN-8
BiE + NSA

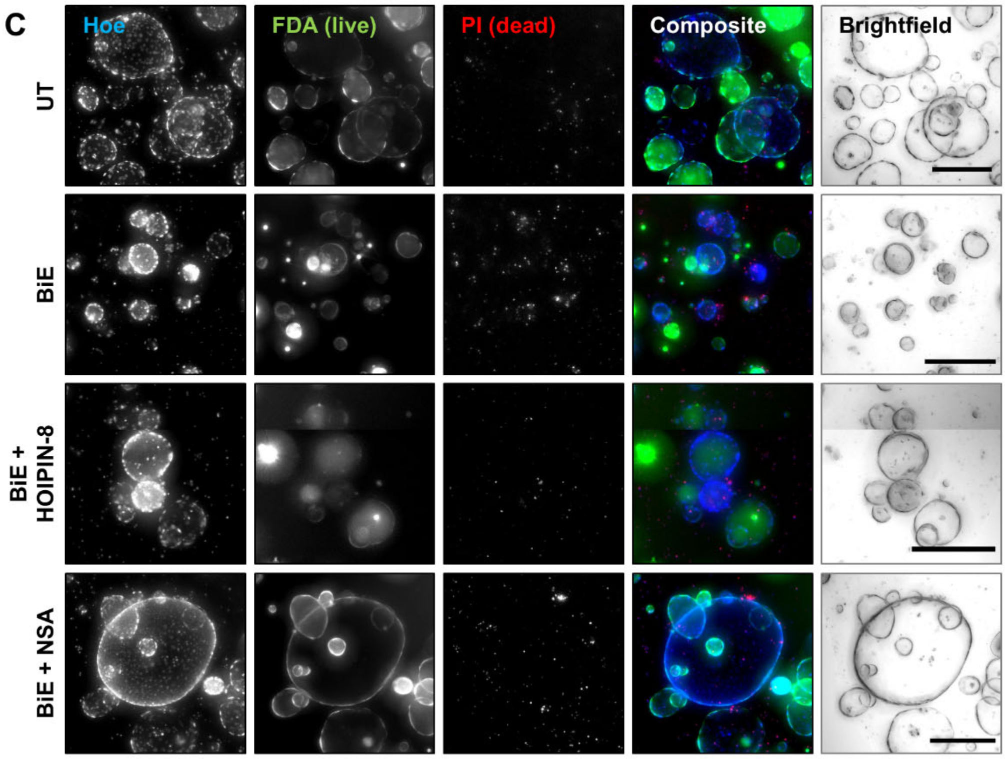

Figure S6. Related to Figure 6. A. Quantification of growth of untreated (UT), BV6 (1 µM) and Emricasan (10 µM) (BE)-treated and HOIPIN-8 (30 µM)-pre-treated and BE (BE+HOIPIN-8)-treated primary hPOs for a total period of 23.5 h. Data are related to Figure 6 and show the normalized mean lumen volume and SEM. **B.** Representative images of time-lapse videos of untreated (UT), Birinapant (20 µM) and Emricasan (10 µM) (BiE)-treated, HOIPIN-8 (30 µM)-pre-treated and BiE-treated (BiE+HOIPIN-8) and NSA (10 µM)-pre-treated and BiE-treated (BiE+NSA) primary hPOs for a total period of 23.5 h. Scalebars: 250 µm. **C.** *Idem* as B., but treated primary hPOs were stained with Hoechst33342 (Hoe) (blue), FDA (live; green) and PI (dead; red) after 24 h treatment with BiE, BiE+HOIPIN-8 or BiE+NSA prior to imaging. Representative images of at least two independent experiments are shown (**B**, **C**). Scalebars: 250 µm.

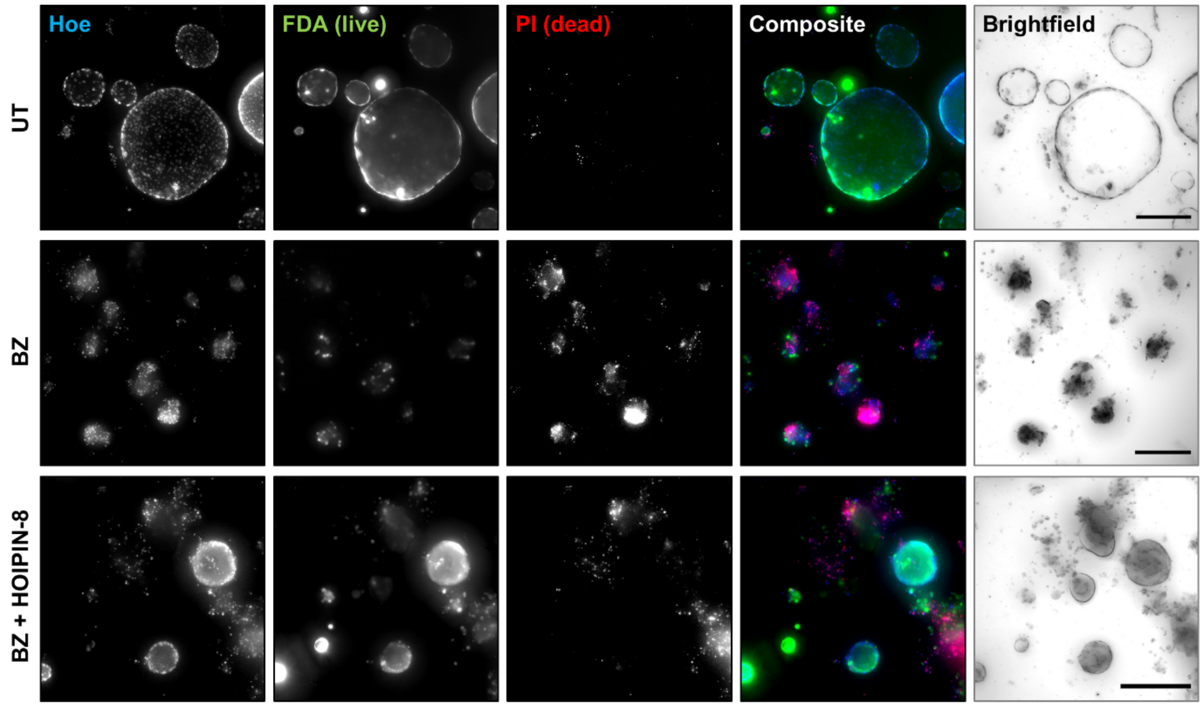

Figure S7. Related to Figure 6. Representative images of untreated (UT), BV6 (1 µM) and zVAD.fmk (20 µM) (BZ)-treated or HOIPIN-8 (30 µM)-pre-treated and BZ (1 µM BV6, 20 µM zVAD.fmk)-treated (BZ+HOIPIN-8) primary hPOs that were stained with Hoechst33342 (Hoe) (blue), FDA (live; green) and PI (dead; red) after 24 h treatment prior to imaging. Representative images of at least two independent experiments are shown. Scalebars: 250 µm.

Movie 1. Related to Figure 6. A. Time-lapse videos of untreated (UT), BV6 (1 µM) and Emricasan (10 µM) (BE)-treated or HOIPIN-8 (30 µM)-pre-treated and BE (BE+HOIPIN-8)-treated primary hPOs for a total period of 23.5 h. Scalebars: 250 µm.

Movie 2. Related to Figure S6. A. Time-lapse videos of untreated (UT), Birinapant (20 µM) and Emricasan (10 µM) (BiE)-treated, HOIPIN-8 (30 µM)-pre-treated and BiE (BiE+HOIPIN-8)-treated or NSA (10 µM)-pre-treated and BiE (BiE+NSA)-treated primary hPOs for a total period of 23.5 h. Scalebars: 250 µm.
