# Supplementary material for: LUBAC-mediated M1 Ub regulates necroptosis by segregating the cellular distribution of active MLKL
Source: Cell Death Dis. 2024 Jan 20;15(1):77. doi: 10.1038/s41419-024-06447-6 (PMC10799905; doi:10.1038/s41419-024-06447-6)
Supplement: Supplementary file 2 — Original Data File [file 41419_2024_6447_MOESM2_ESM.pdf]

D

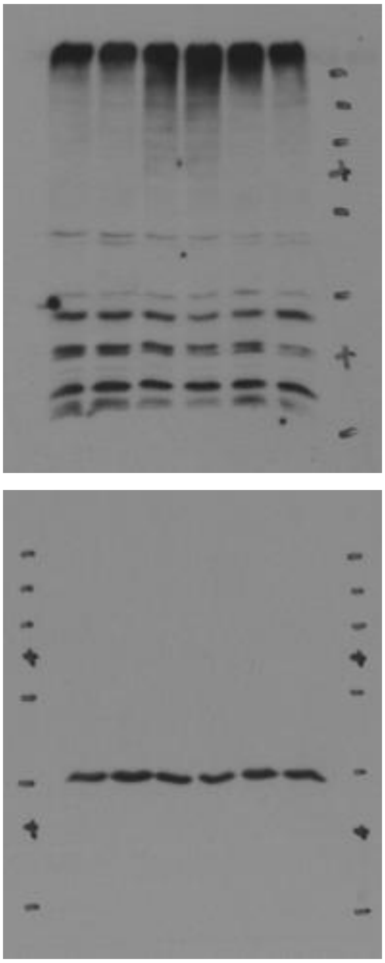

E

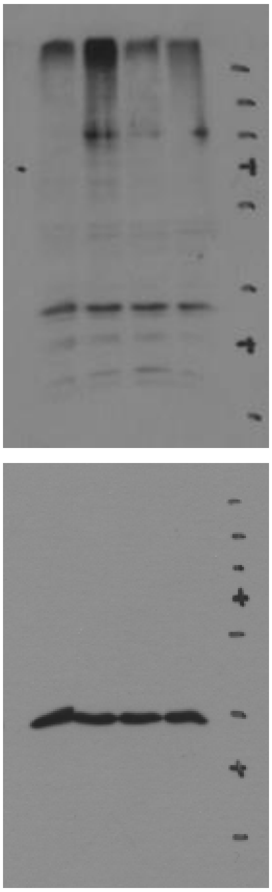

Figure 1

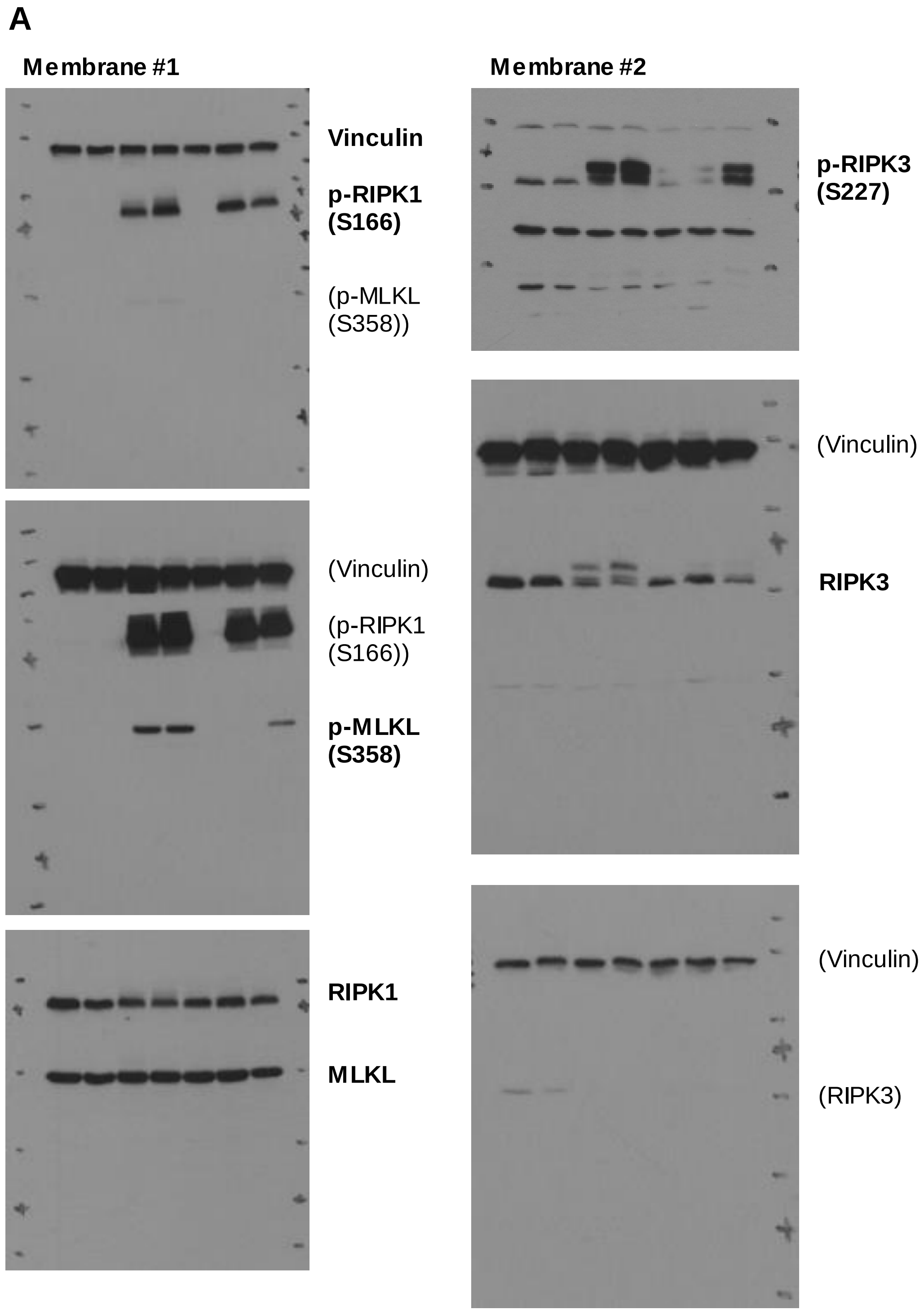
A
Membrane #1
Membrane #2
Vinculin
p-RIPK1
(S166)
(p-MLKL
(S358))
p-RIPK3
(S227)
(Vinculin)
(p-RIPK1
(S166))
p-MLKL
(S358)
RIPK3
RIPK1
MLKL
(Vinculin)
(RIPK3)

Figure 2

B

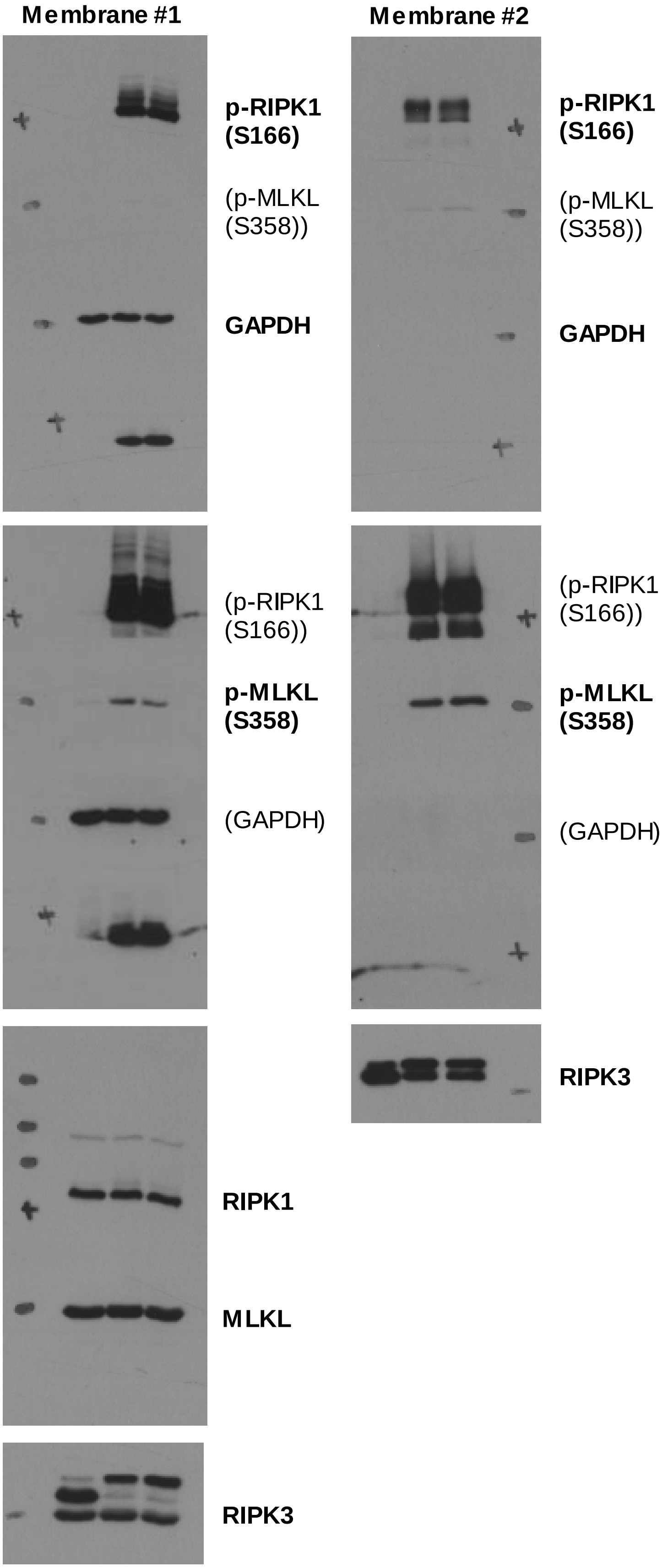

Membrane #1
Membrane #2
p-RIPK1 (S166)
(p-MLKL (S358))
GAPDH
(p-RIPK1 (S166))
p-MLKL (S358)
(GAPDH)
RIPK1
MLKL
RIPK3

Figure 2

C

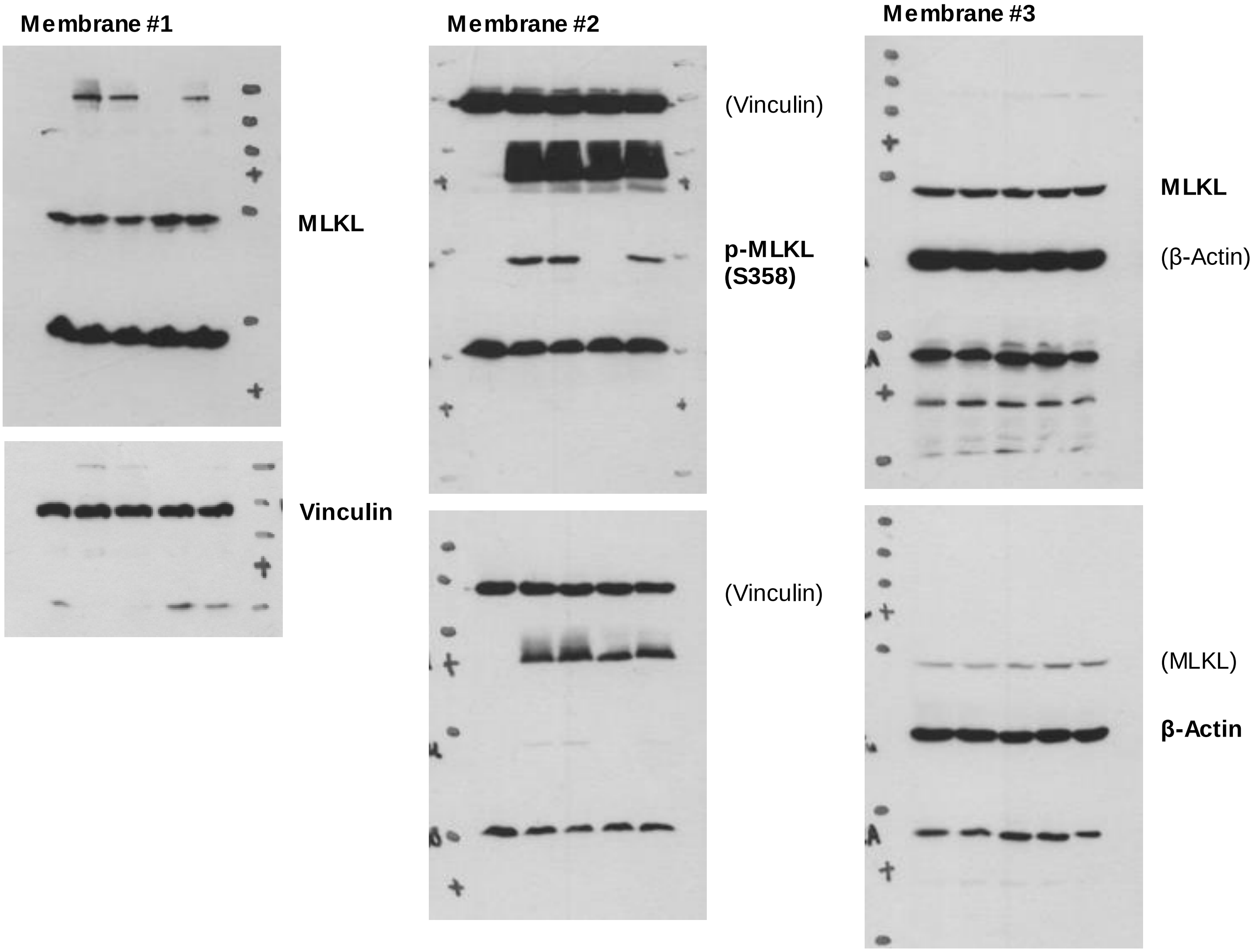
Membrane #1
MLKL
Vinculin
Membrane #2
(Vinculin)
p-MLKL
(S358)
(Vinculin)
Membrane #3
MLKL
(β-Actin)
(MLKL)
β-Actin

Figure 2

D

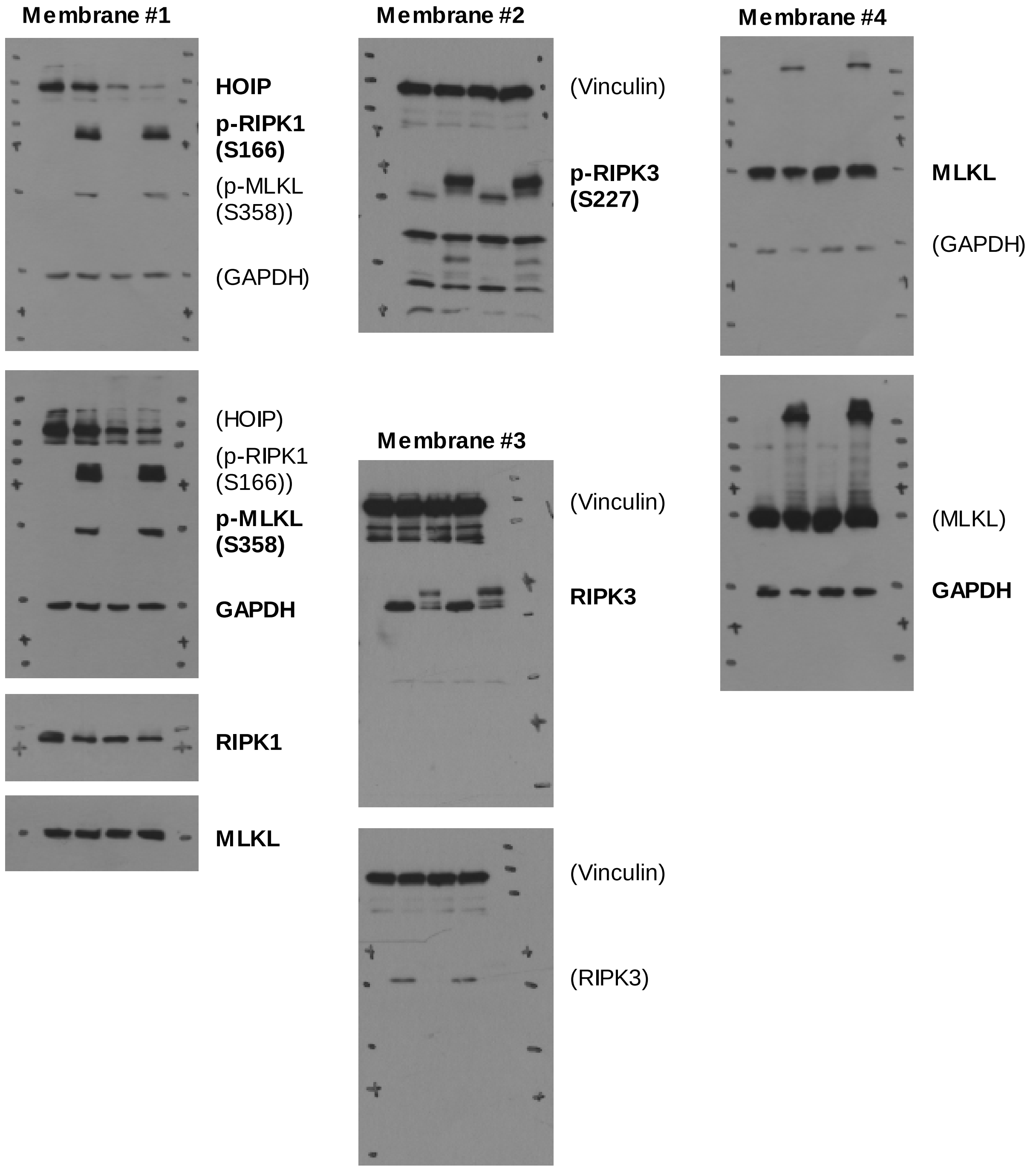
Membrane #1
HOIP
p-RIPK1
(S166)
(p-MLKL
(S358))
(GAPDH)
(HOIP)
(p-RIPK1
(S166))
p-MLKL
(S358)
GAPDH
RIPK1
MLKL
Membrane #2
(Vinculin)
p-RIPK3
(S227)
Membrane #3
(Vinculin)
RIPK3
(Vinculin)
(RIPK3)
Membrane #4
MLKL
(GAPDH)
(MLKL)
GAPDH

Figure 2

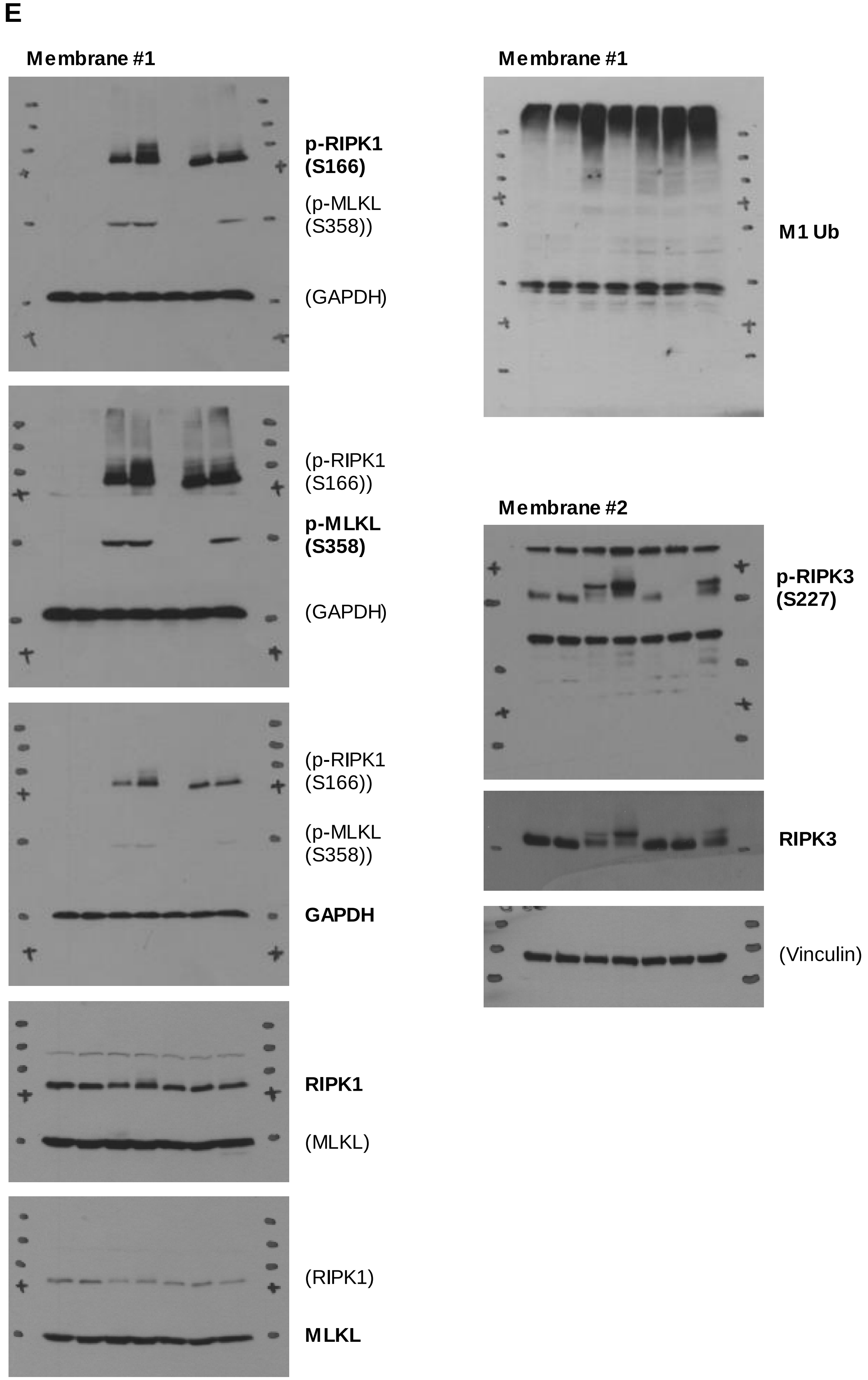
E
Membrane #1
p-RIPK1 (S166)
(p-MLKL (S358))
(GAPDH)
(p-RIPK1 (S166))
p-MLKL (S358)
(GAPDH)
(p-RIPK1 (S166))
(p-MLKL (S358))
GAPDH
RIPK1
(MLKL)
(RIPK1)
MLKL
Membrane #1
M1 Ub
Membrane #2
p-RIPK3 (S227)
RIPK3
(Vinculin)

Figure 2

A

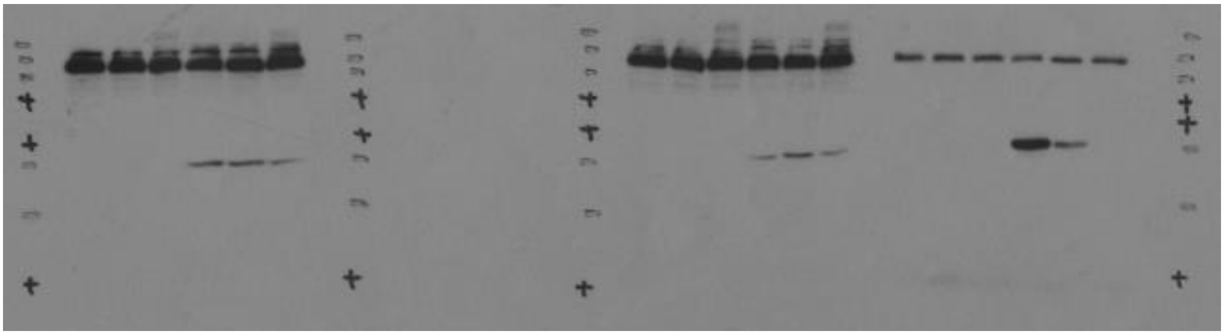

p-MLKL (S358)

(CD9)

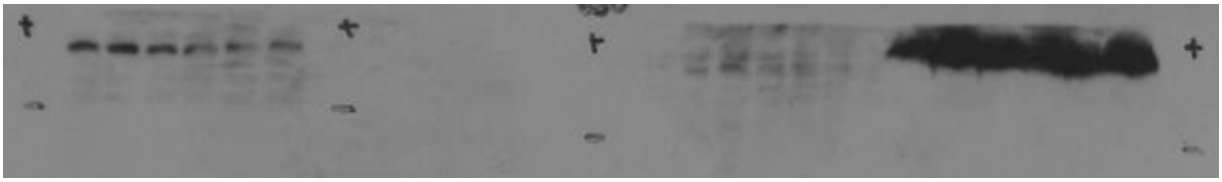

CD9

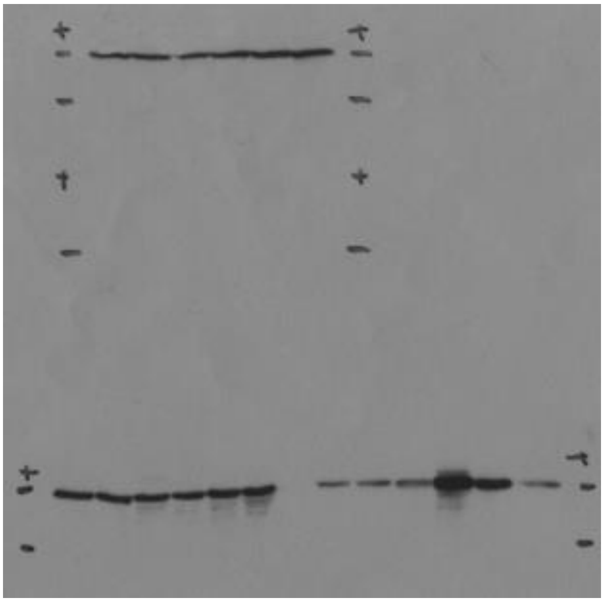

MLKL

MLKL

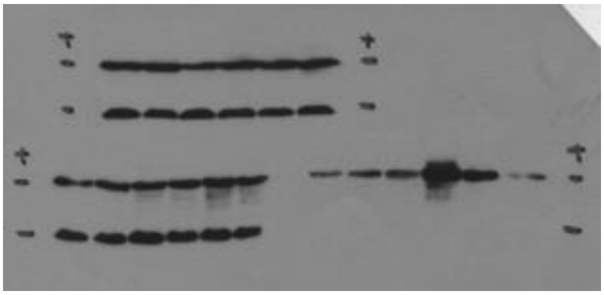

(MLKL)

GAPDH

(MLKL)

GAPDH

Figure 4

C

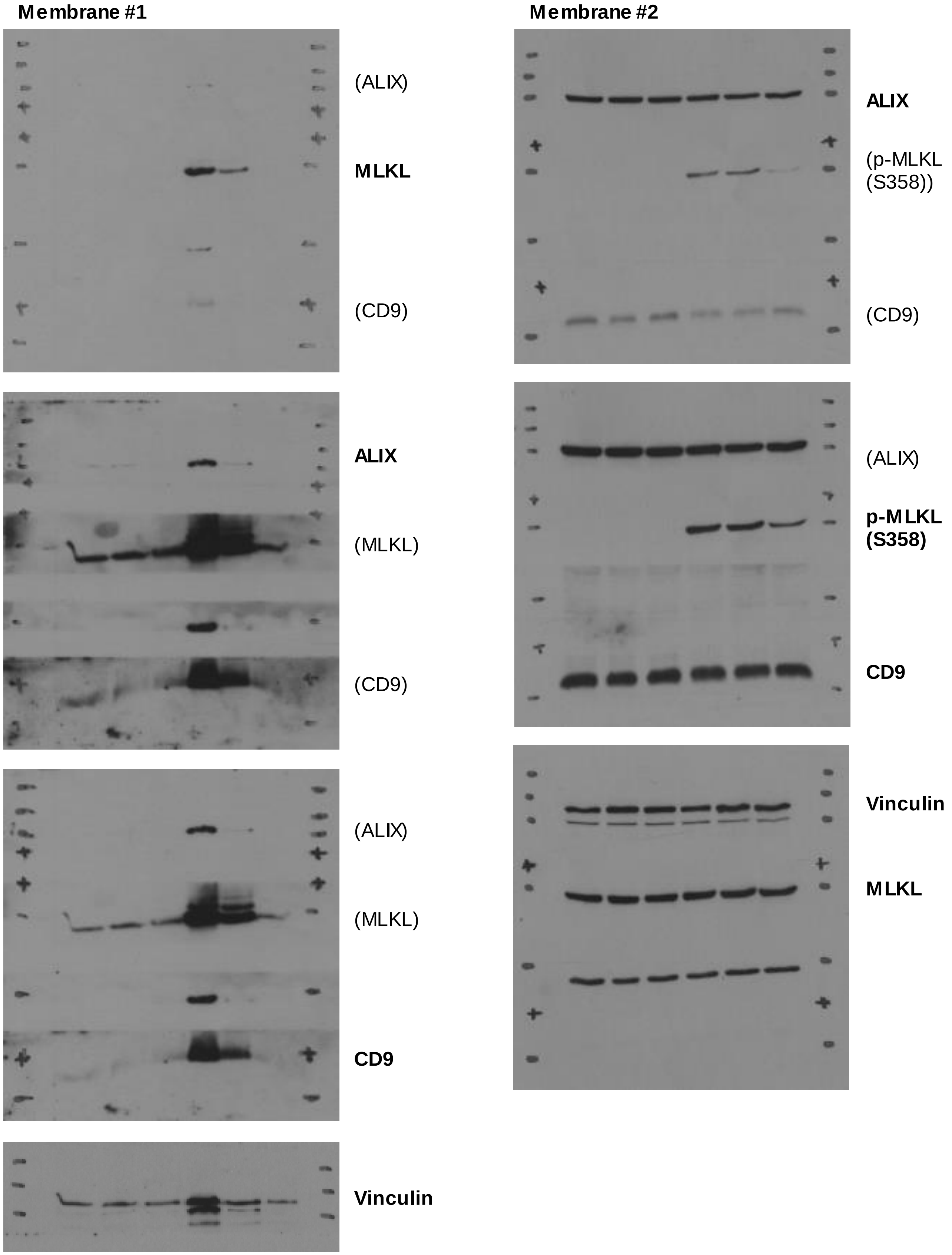
Membrane #1
Membrane #2
(ALIX)
MLKL
(CD9)
ALIX
(p-MLKL
(S358))
(CD9)
ALIX
(MLKL)
(CD9)
(ALIX)
p-MLKL
(S358)
CD9
(ALIX)
(MLKL)
CD9
Vinculin
MLKL
Vinculin

Figure 4

D

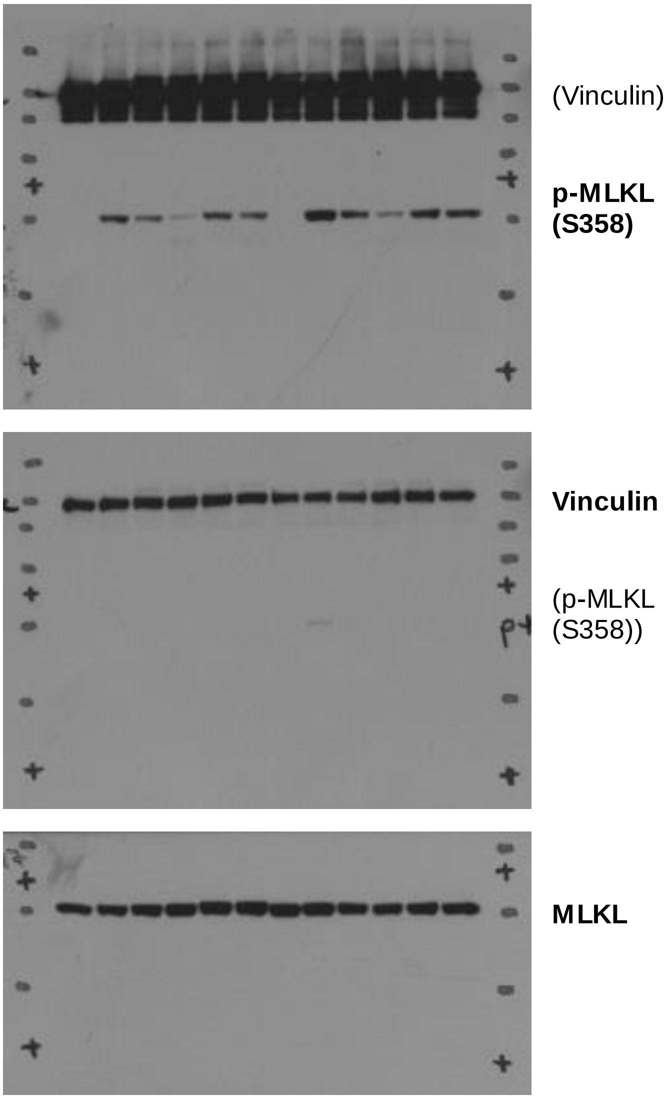

Figure 4

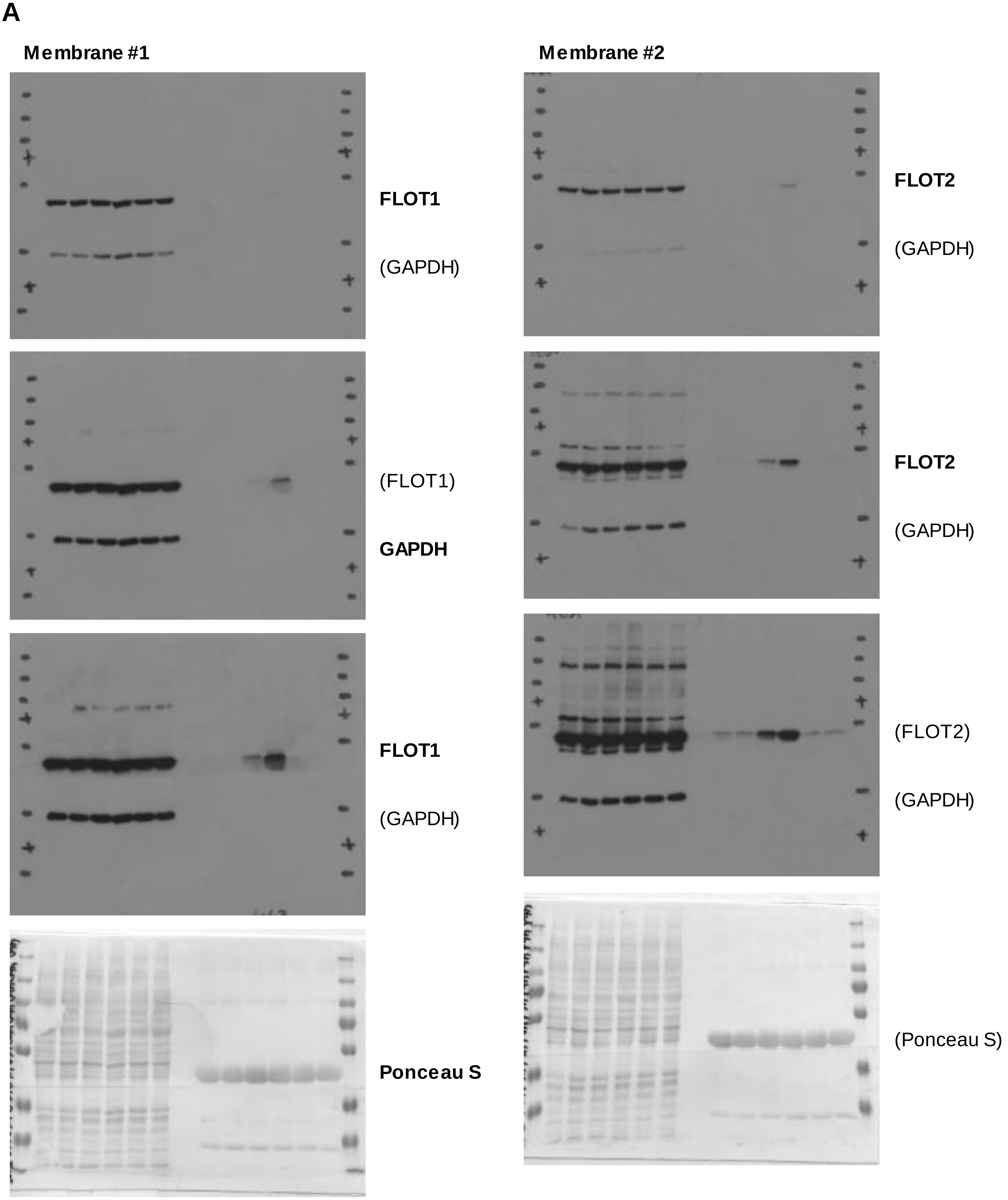
A
Membrane #1
FLOT1
(GAPDH)
(FLOT1)
GAPDH
FLOT1
(GAPDH)
Ponceau S
Membrane #2
FLOT2
(GAPDH)
FLOT2
(GAPDH)
(FLOT2)
(GAPDH)
(Ponceau S)

Figure 5

A

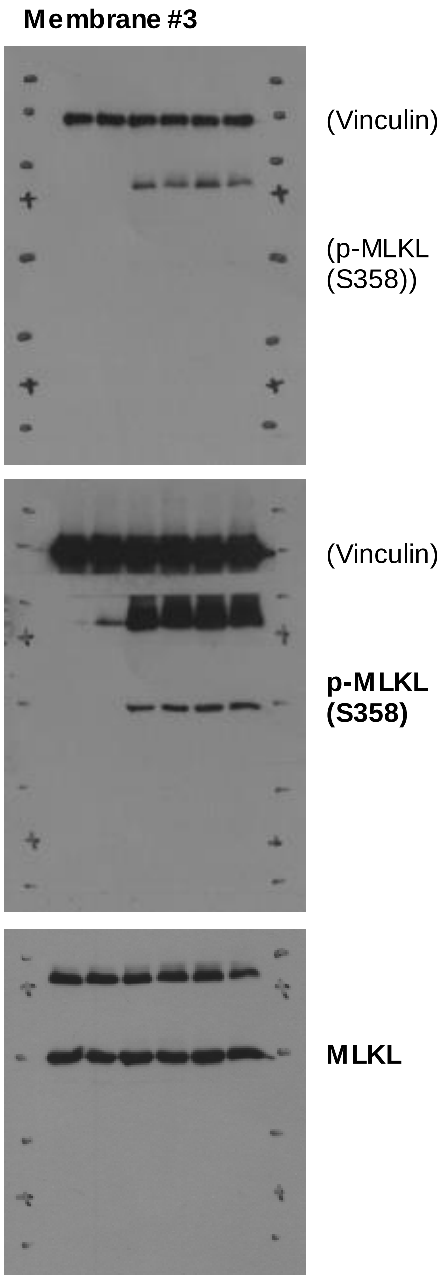

Figure 5

B

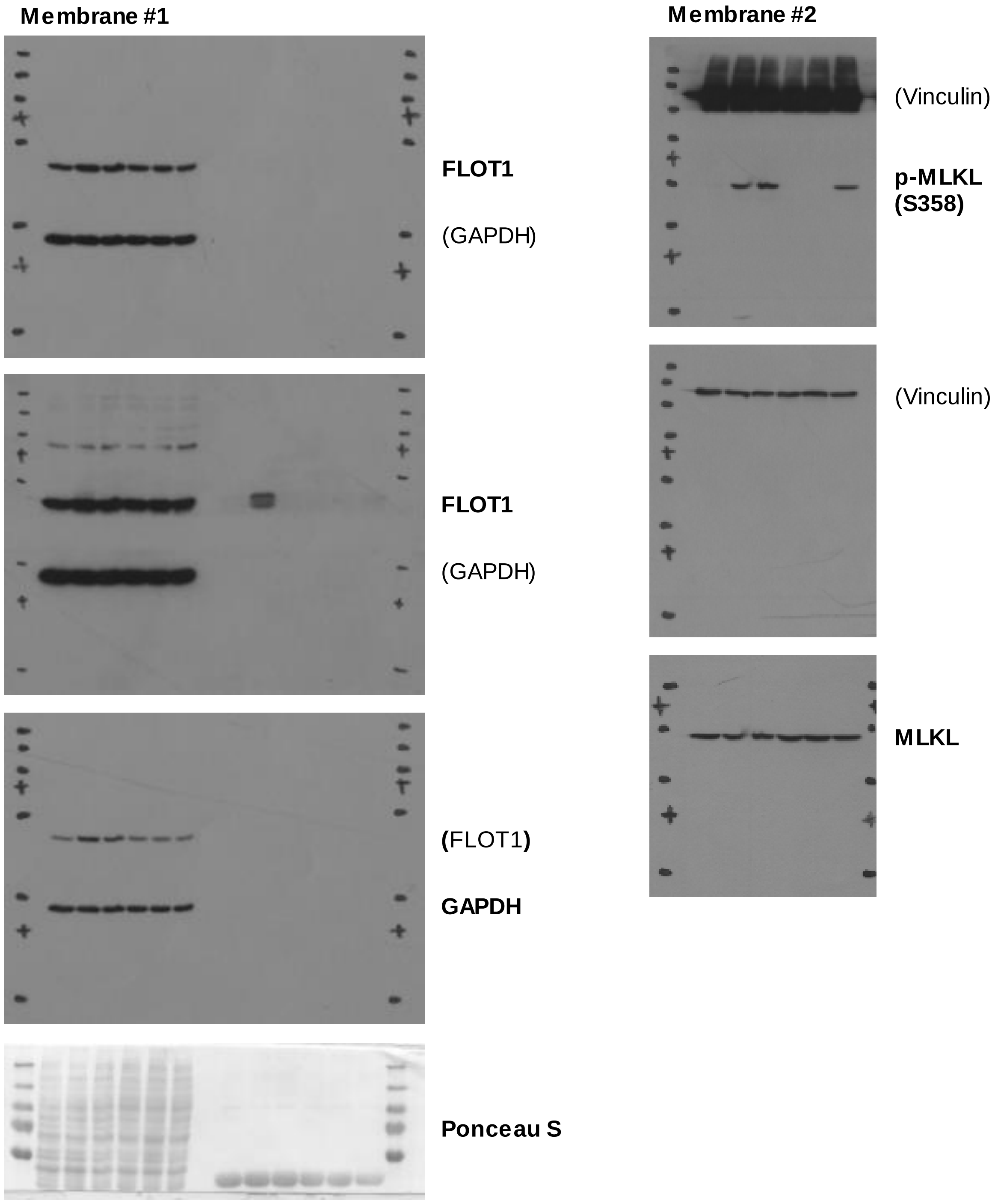

Membrane #1
FLOT1
(GAPDH)
FLOT1
(GAPDH)
(FLOT1)
GAPDH
Ponceau S
Membrane #2
(Vinculin)
p-MLKL
(S358)
(Vinculin)
MLKL

Figure 5

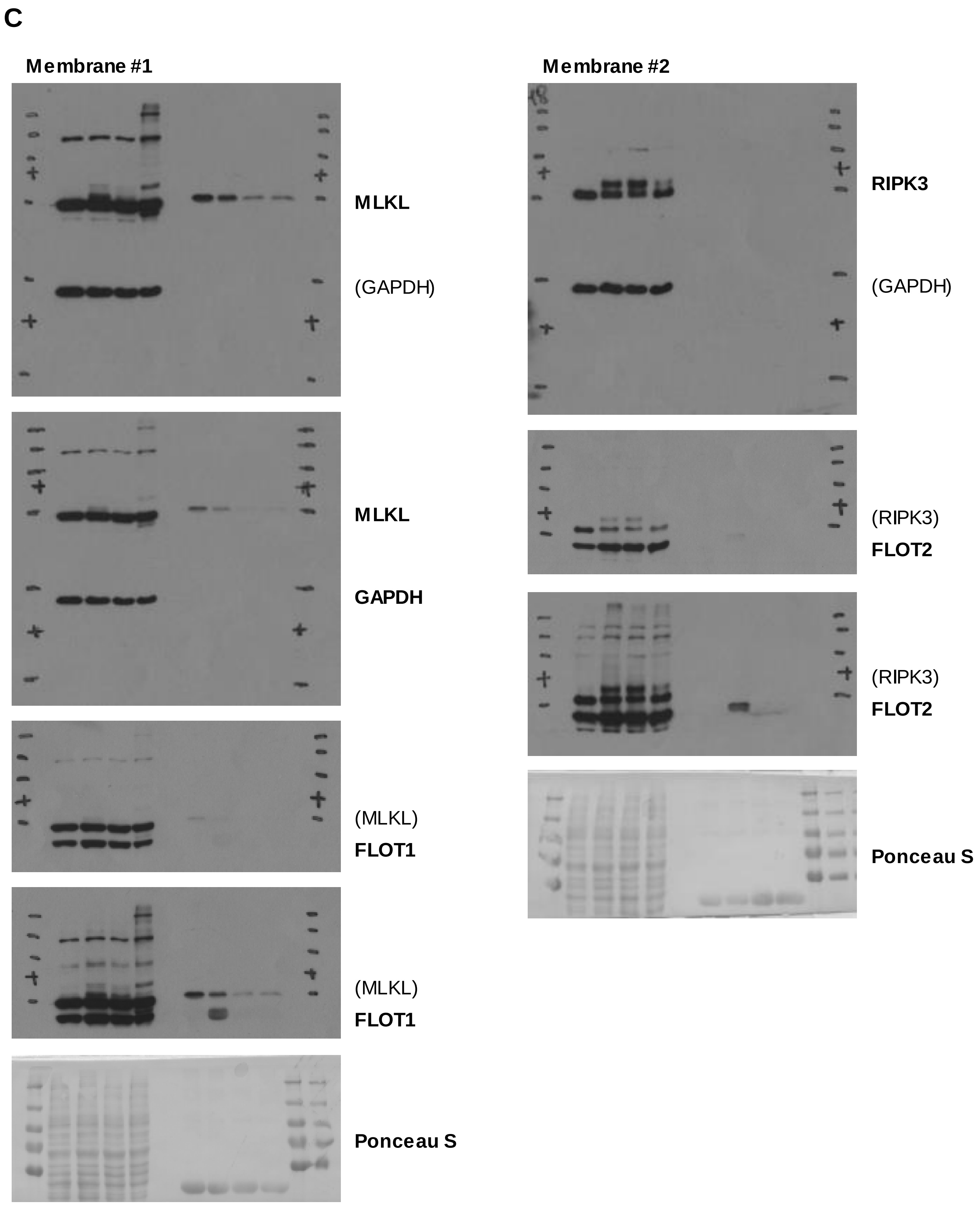
C
Membrane #1
MLKL
(GAPDH)
MLKL
GAPDH
(MLKL)
FLOT1
(MLKL)
FLOT1
Ponceau S
Membrane #2
RIPK3
(GAPDH)
(RIPK3)
FLOT2
(RIPK3)
FLOT2
Ponceau S

Figure 5

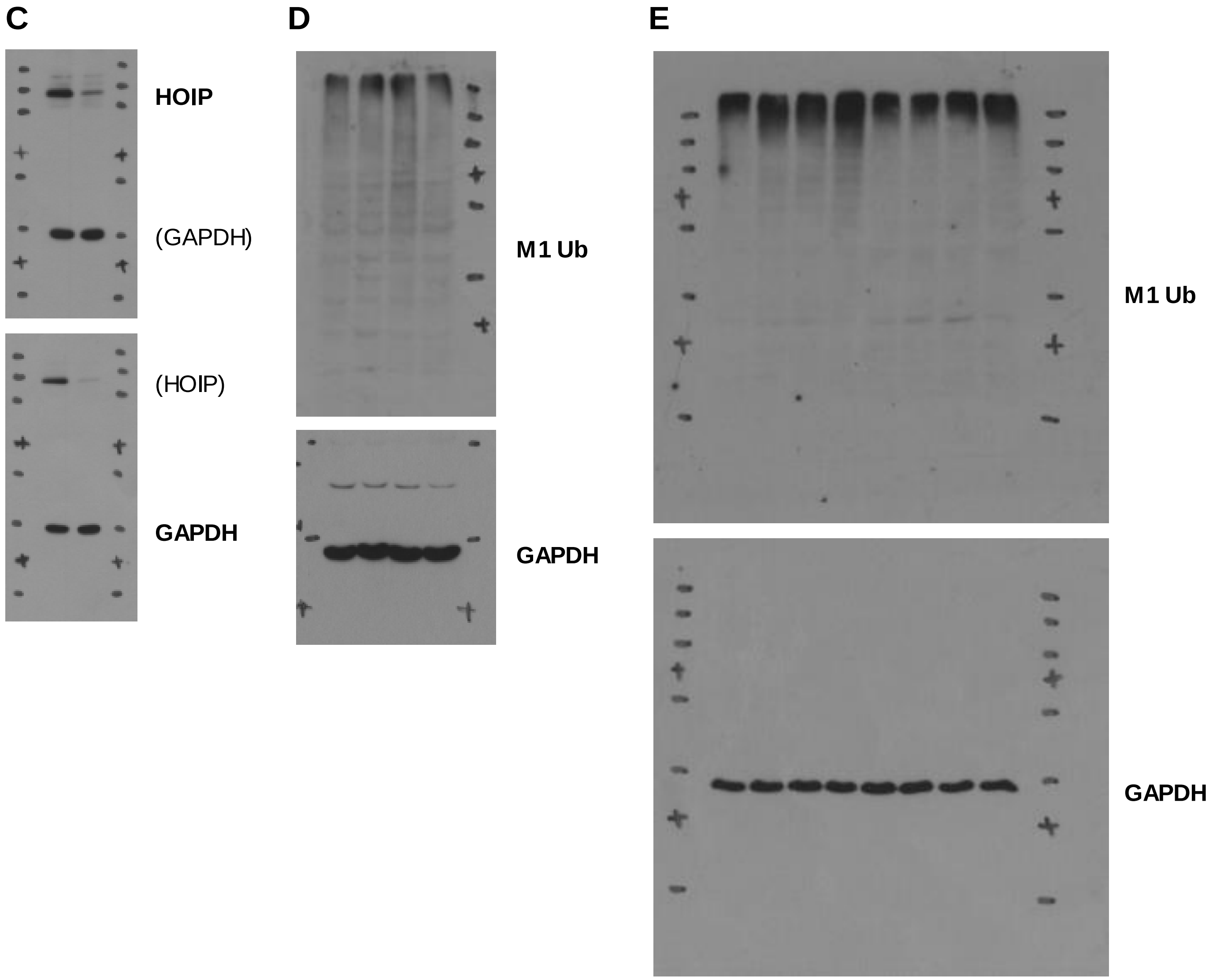

Figure S1

F

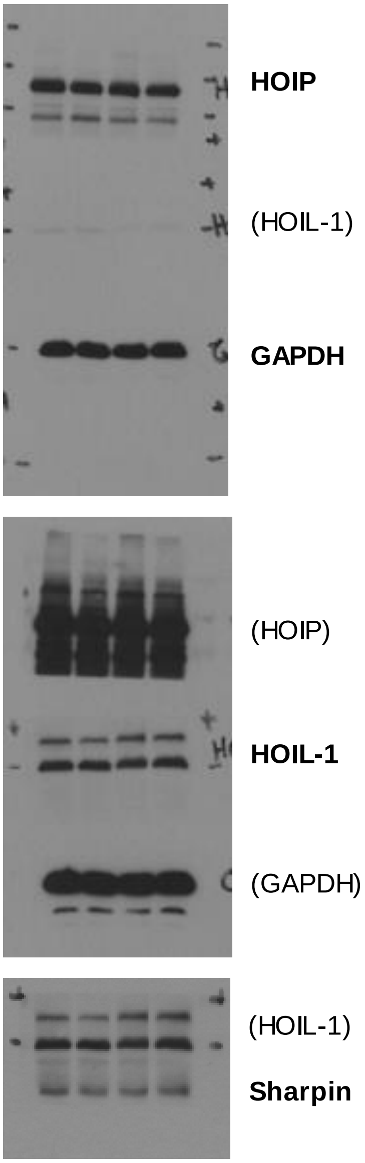
HOIP
(HOIL-1)
GAPDH
(HOIP)
HOIL-1
(GAPDH)
(HOIL-1)
Sharpin

G

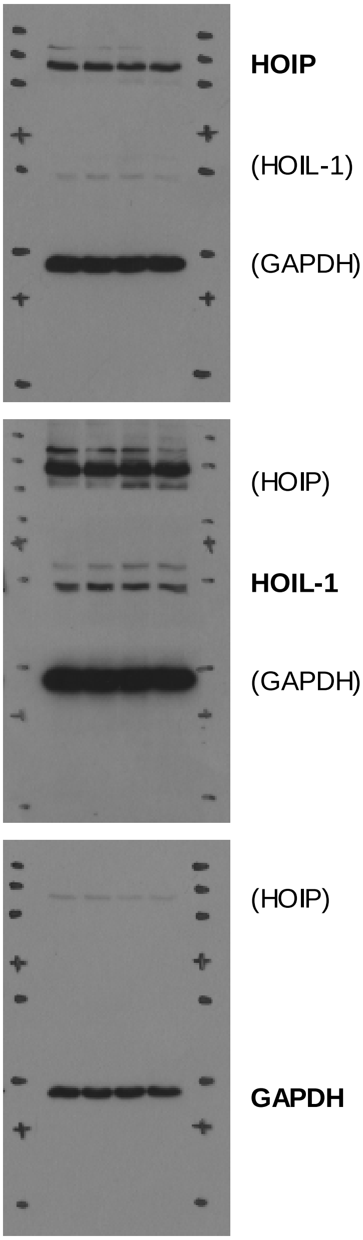
HOIP
(HOIL-1)
(GAPDH)
(HOIP)
HOIL-1
(GAPDH)
(HOIP)
GAPDH

Figure S1

I

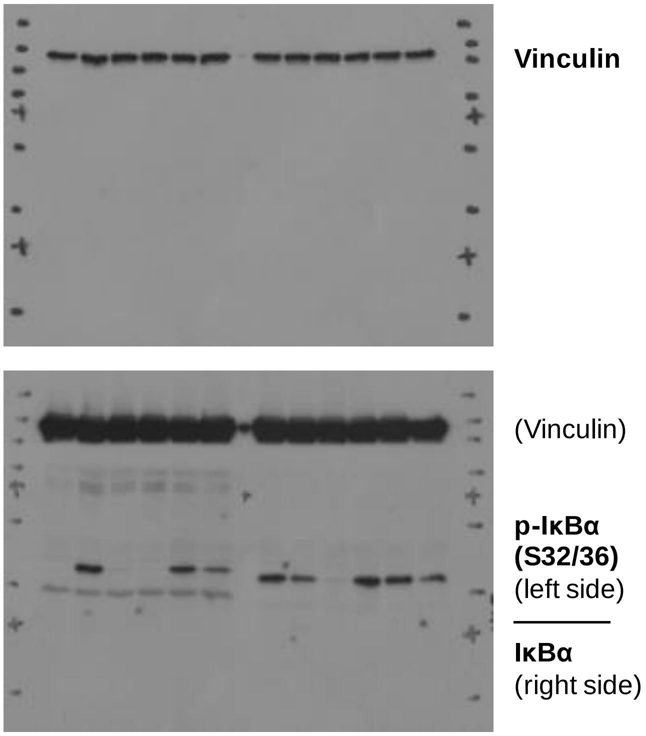

J

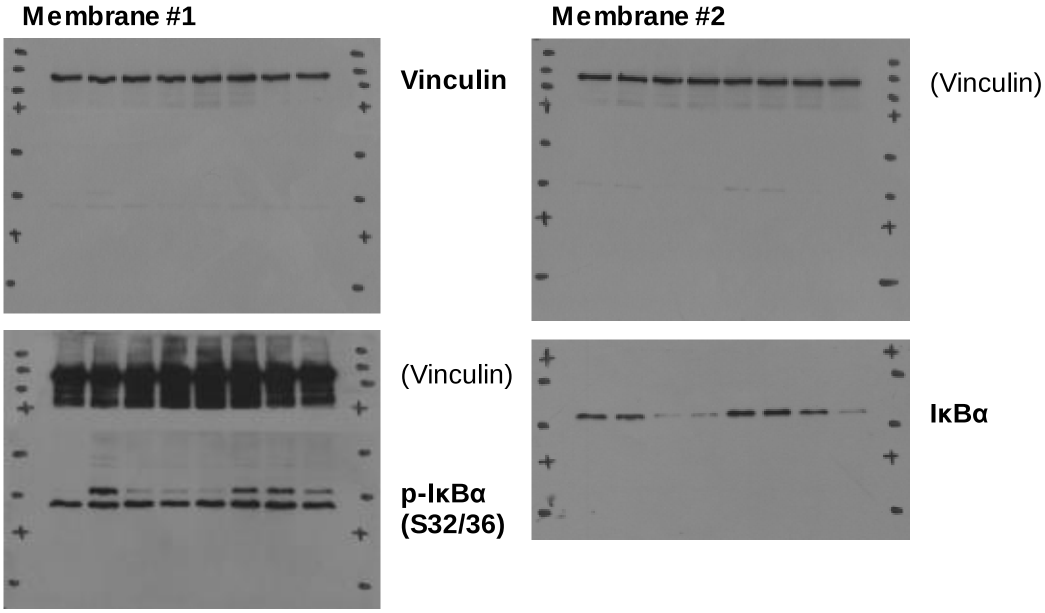

N

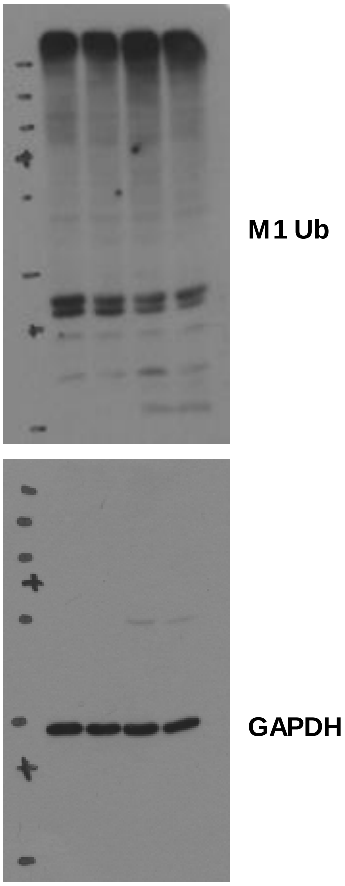

Figure S1

A

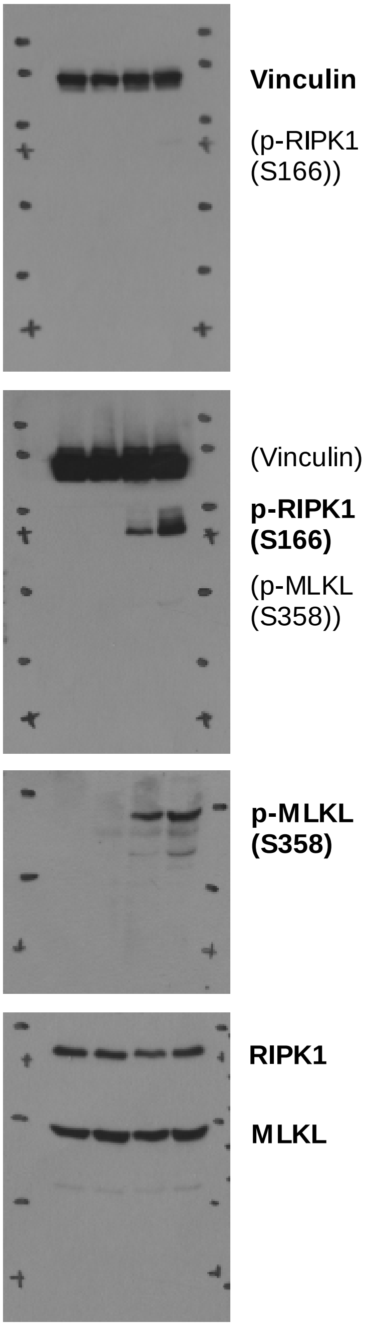
Vinculin
(p-RIPK1
(S166))
(Vinculin)
p-RIPK1
(S166)
(p-MLKL
(S358))
p-MLKL
(S358)
RIPK1
MLKL

Figure S2

B

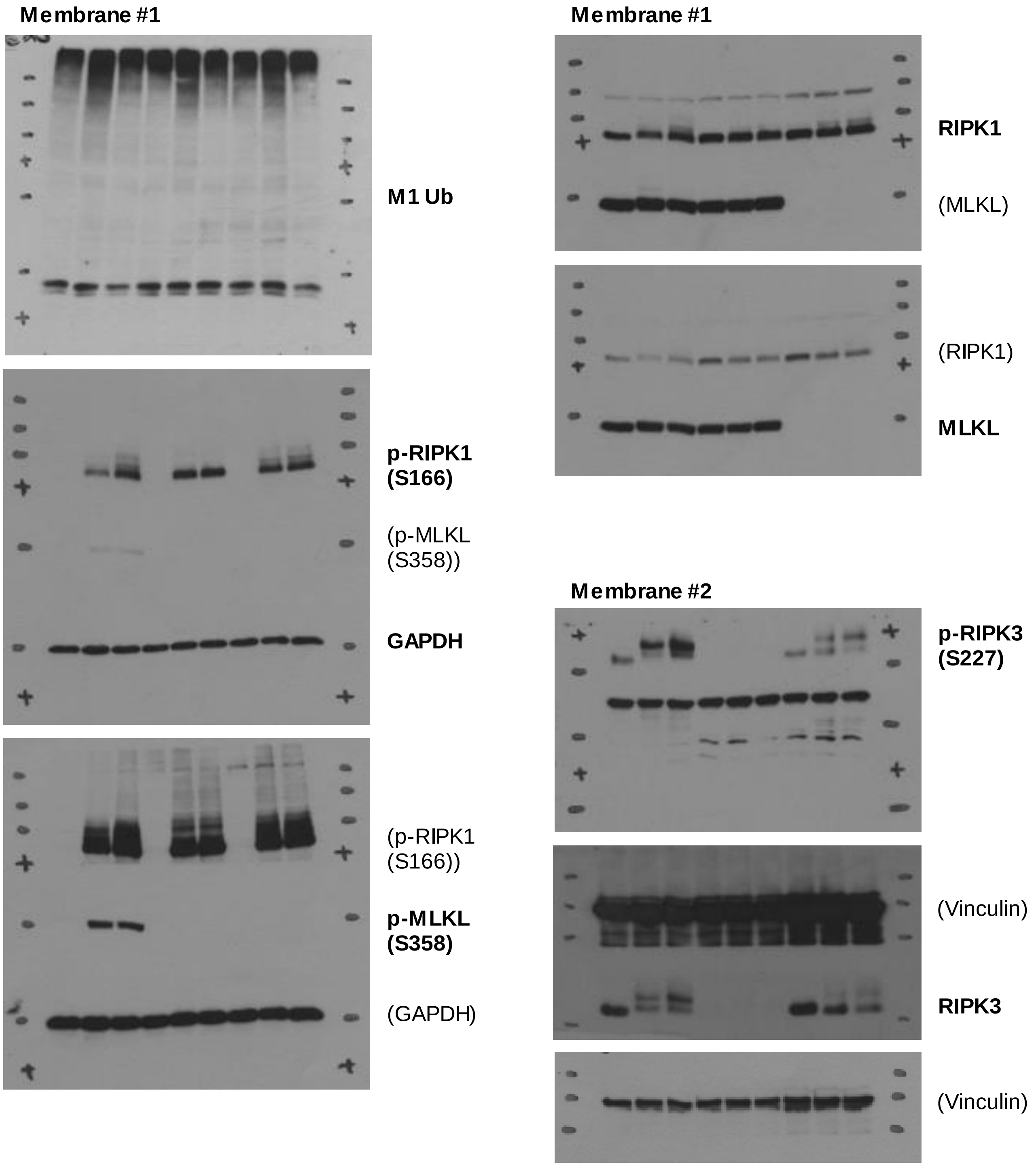
Membrane #1
M1 Ub
p-RIPK1
(S166)
(p-MLKL
(S358))
GAPDH
(p-RIPK1
(S166))
p-MLKL
(S358)
(GAPDH)
Membrane #1
RIPK1
(MLKL)
(RIPK1)
MLKL
Membrane #2
p-RIPK3
(S227)
(Vinculin)
RIPK3
(Vinculin)

Figure S2

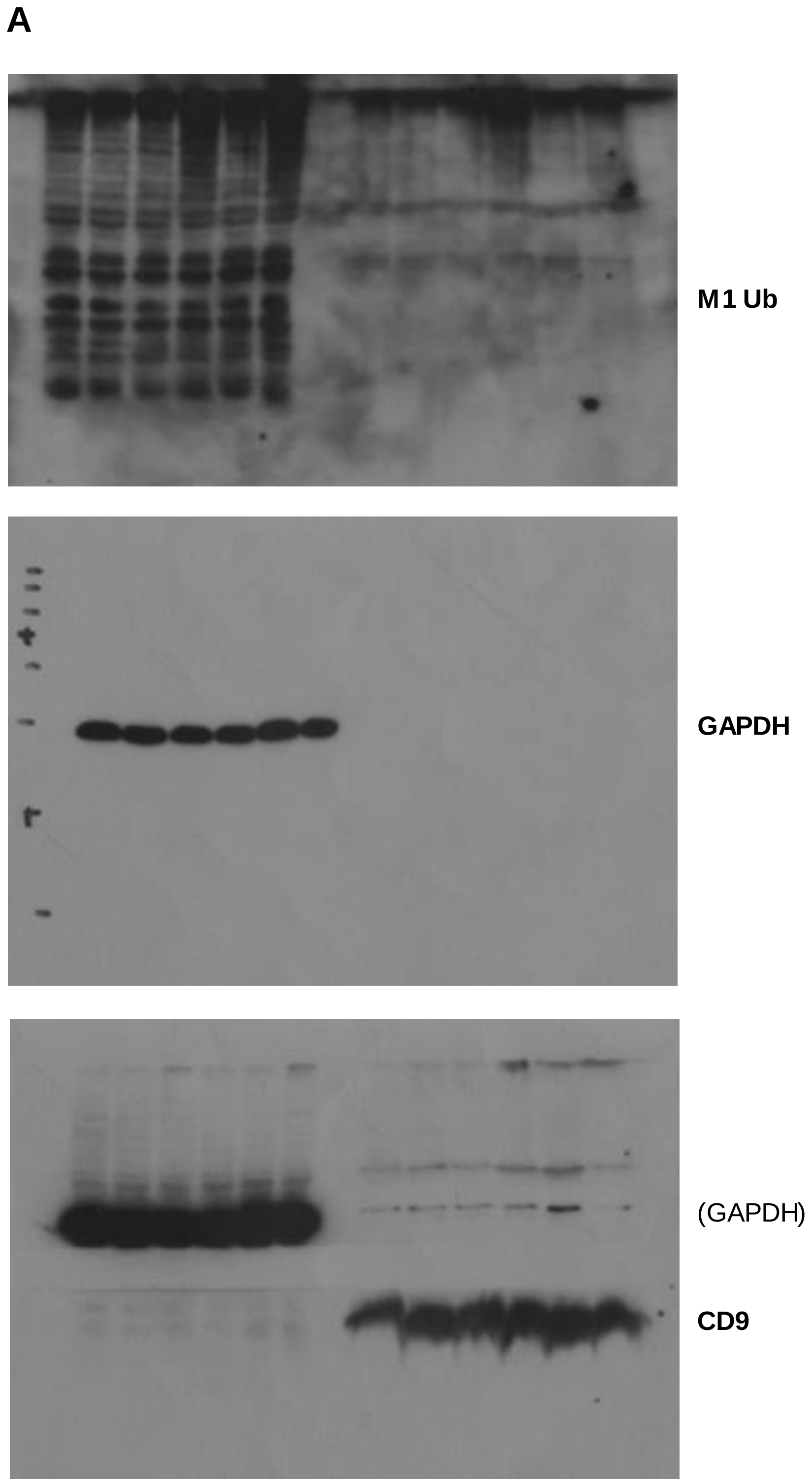

Figure S4

A

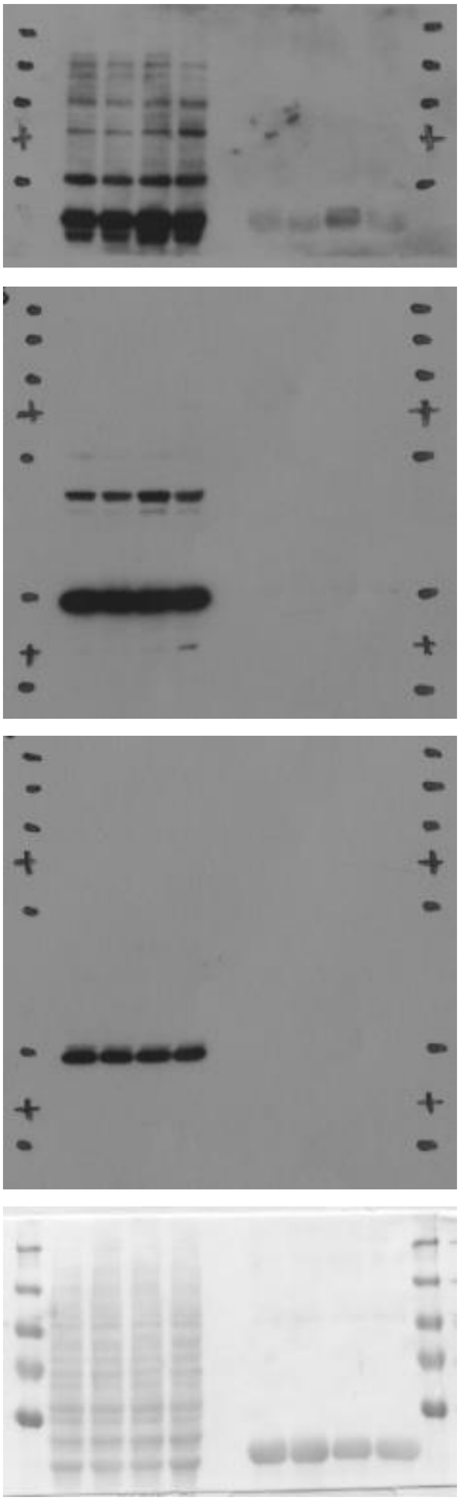

Figure S5

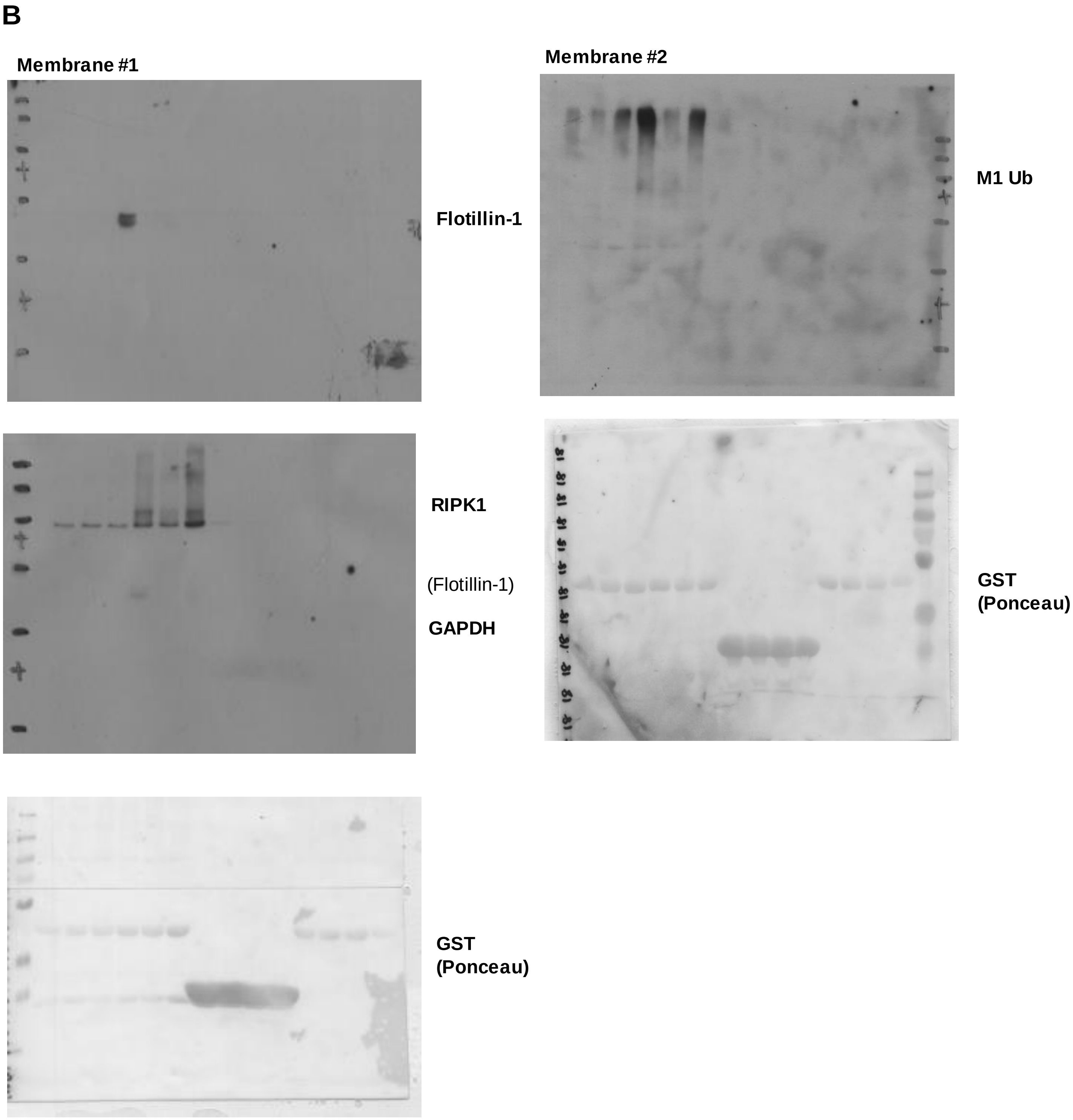
B
Membrane #1
Flotillin-1
RIPK1
(Flotillin-1)
GAPDH
GST
(Ponceau)
Membrane #2
M1 Ub
GST
(Ponceau)

Figure S5

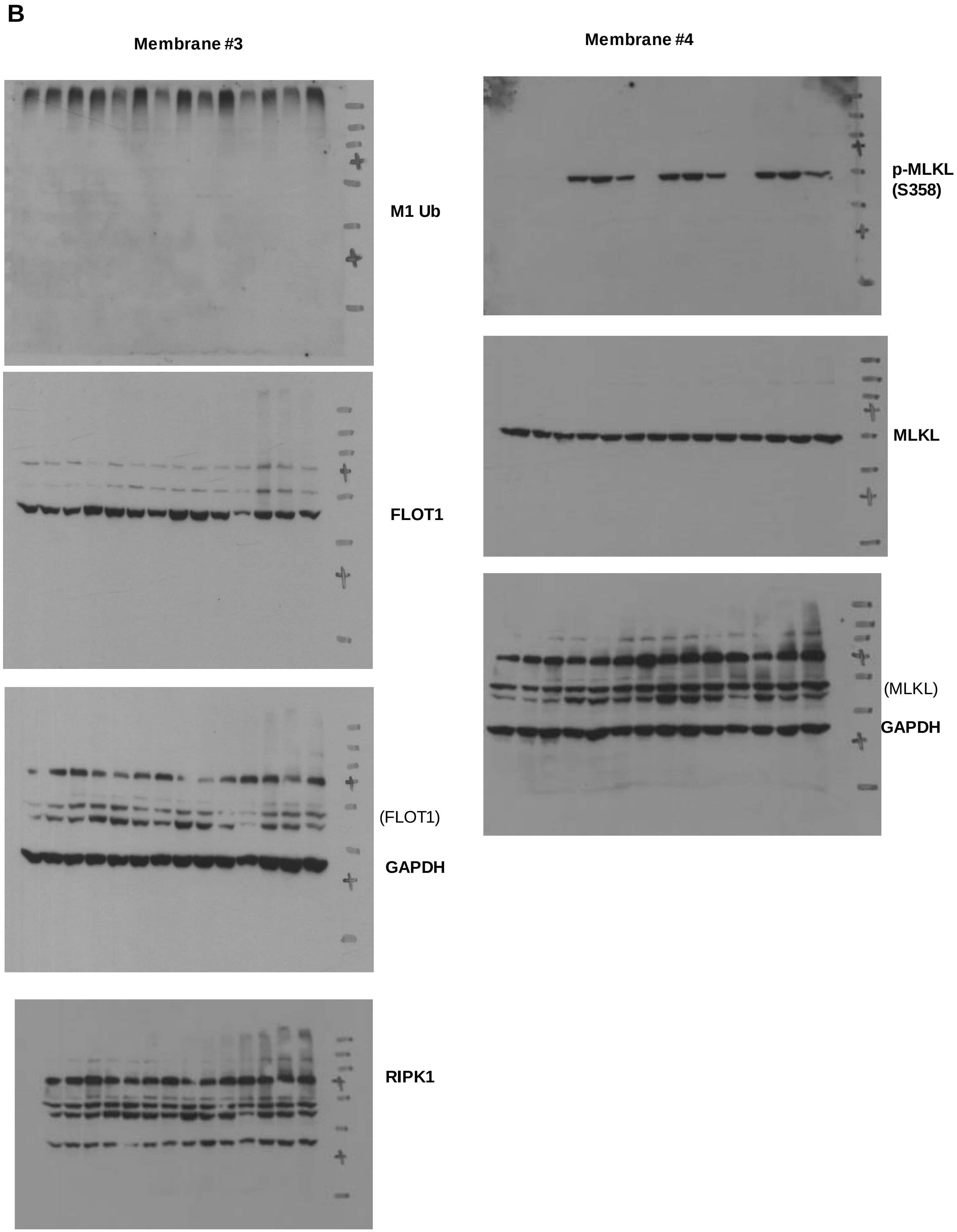

B
Membrane #3
Membrane #4
M1 Ub
FLOT1
(FLOT1)
GAPDH
RIPK1
p-MLKL
(S358)
MLKL
(MLKL)
GAPDH

Figure S5

C **Membrane #1**

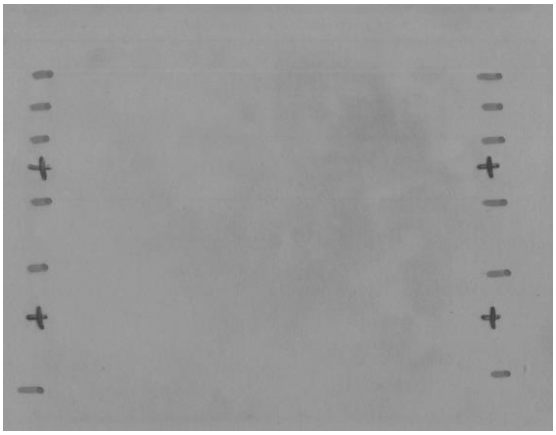

M1 Ub

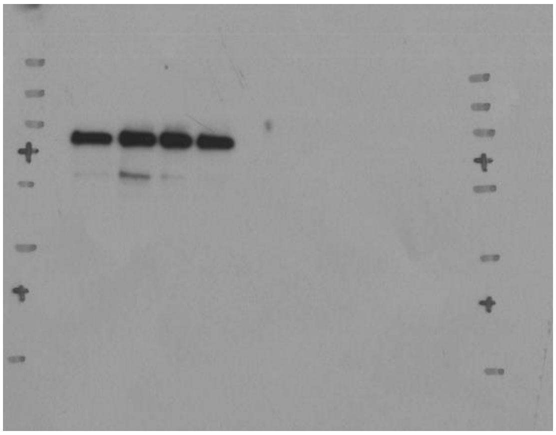

Vinculin

Flotillin-2
(GFP_Flottilin-2)

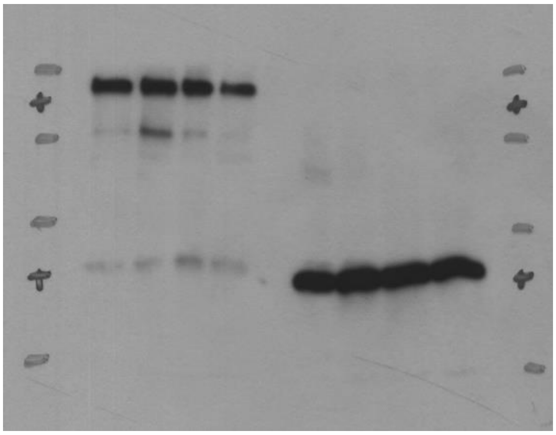

GFP(GFP_Flottilin-2)

GFP

Figure S5

C

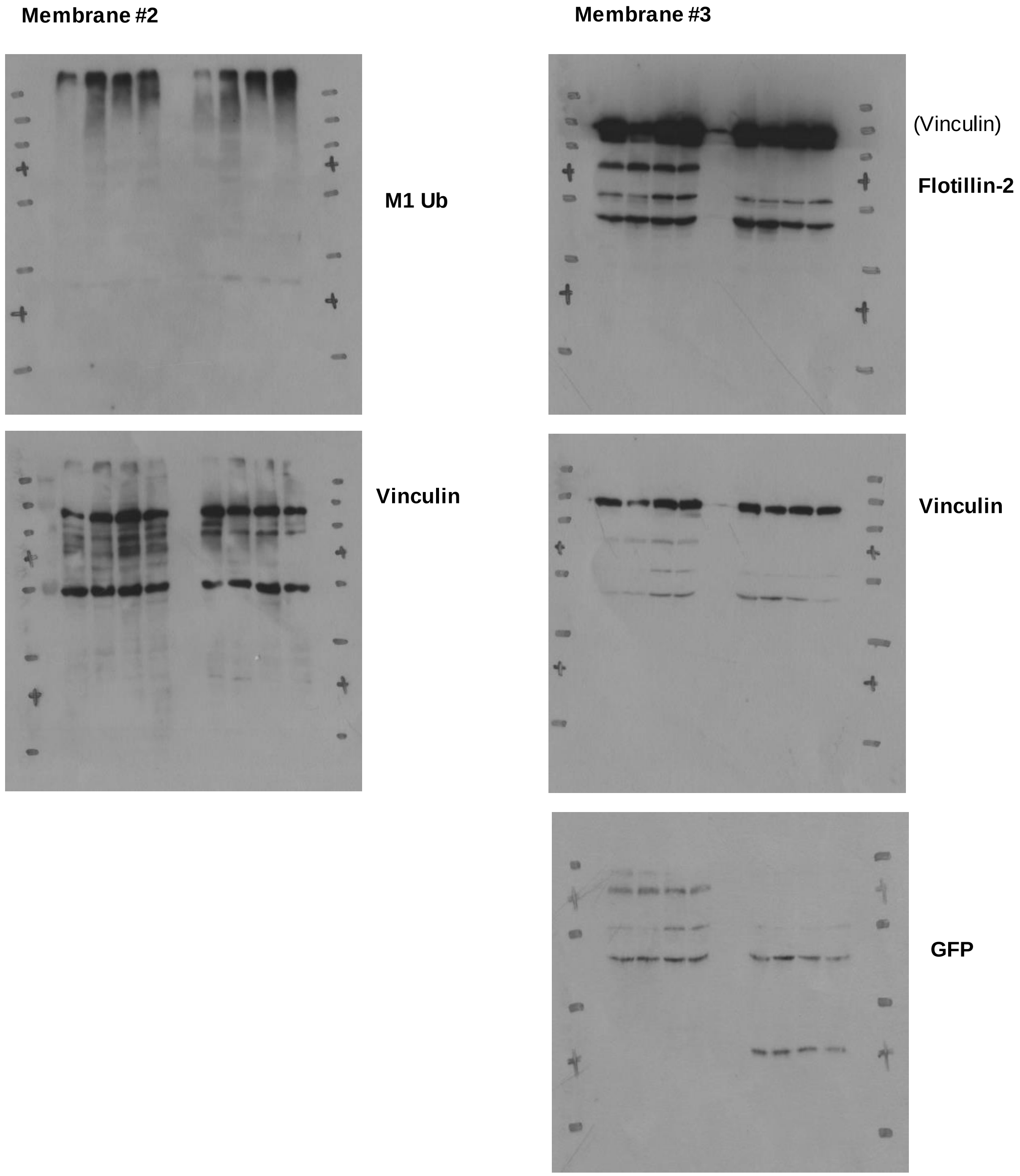
Membrane #2
Membrane #3
M1 Ub
Vinculin
(Vinculin)
Flotillin-2
Vinculin
GFP

Figure S5

D

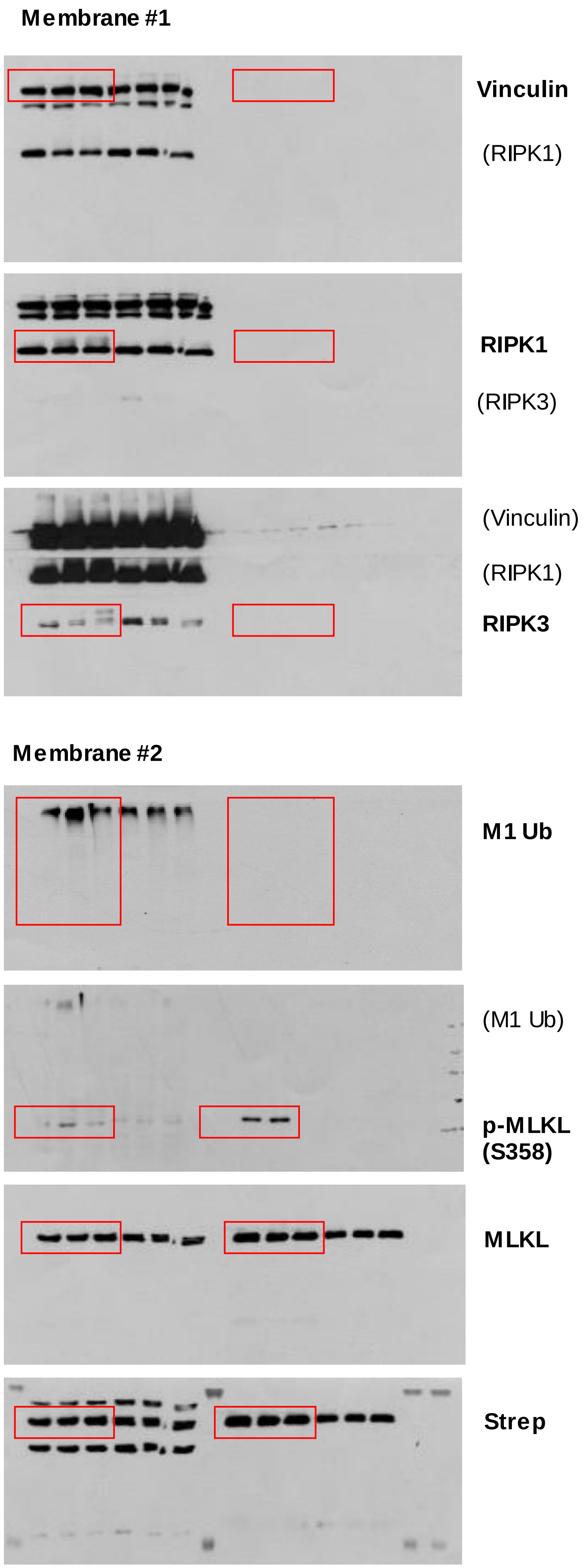
Membrane #1
Vinculin
(RIPK1)
RIPK1
(RIPK3)
(Vinculin)
(RIPK1)
RIPK3
Membrane #2
M1 Ub
(M1 Ub)
p-MLKL
(S358)
MLKL
Strep

Figure S5
